# Low-mass zinc pools in *Escherichia coli*: Micromolar concentrations, diverse compositions, and Zn-glutathione dominating under Zn-replete conditions

**DOI:** 10.1016/j.jbc.2025.110362

**Published:** 2025-07-29

**Authors:** Alexia C. Kreinbrink, Nicholas Romano, Justin D. Hierholzer, Paul A. Lindahl

**Affiliations:** 1 Department of Biochemistry and Biophysics, Texas A&M University, College Station, Texas, USA; 2 Department of Chemical Engineering, Texas A&M University, College Station, Texas, USA; 3 Department of Chemistry, Texas A&M University, College Station, Texas, USA

**Keywords:** cytoplasm, chelatable, chromatography, ESI-MS, “free” zinc, glutathione, ICP-MS, labile zinc pool, Mössbauer spectroscopy, sulfur

## Abstract

Most zinc in cells is bound to proteins, but a tiny portion is bound to non-proteinaceous Zn complexes coordinated by unidentified metabolites. Such low-mass pools likely play a central role in Zn homeostasis in cells, yet their chemical compositions and concentrations are unestablished. Previous investigations suggest that the collective Zn concentration of so-called “free” or “labile” pools is femtomolar to picomolar. Here the low-mass Zn pool in *Escherichia coli* was investigated by isolating cytoplasm from cells grown in media supplemented with increasing concentrations of Zn(acetate)_2_. Gentle cell lysis was demonstrated using Mössbauer spectroscopy. Corresponding isolated cytoplasm contained increasing concentrations of Zn. Equivalent samples were subjected to liquid chromatography with inline ICP-MS detection. Many Zn and S peaks were detected, due to both proteins and the low-mass pool. Zn complexes were largely stable over several days. Cytoplasm had a large binding capacity for aqueous Zn, implying that cells are essentially devoid of aqueous Zn. Other samples were treated with the chelator N,N,N'N'-tetrakis[(pyridin-2-yl)methyl]ethane-1,2-diamine (TPEN) to evaluate the binding strength of the detected species. TPEN removed Zn from both proteins and pool complexes with about equal propensity. Low-mass zinc complexes were present at μM collective concentrations, orders of magnitude higher than previous reports for “free” or “labile” pools. Two Zn-glutathione (GSH) complexes were identified by electrospray ionization mass spectrometry (ESI-MS) as dominant members of the pool under Zn-replete conditions. Cells do not significantly increase GSH concentrations when exposed to high levels of media Zn; rather, excess imported Zn is sequestered by available abundant GSH.

Zinc is an essential metal in biology, with approximately 10% of eukaryotic genes and 5% of prokaryotic genes encoding zinc-dependent proteins (1, 2, 3). Zn^2+^ ions (to be called Zn) are excellent Lewis acids. This redox-inactive metal is installed into the active site of numerous enzymes, including proteases, nucleases, phosphatases, to serve in this capacity*.* Zn binding can also enforce essential structural features in proteins, including in thousands of Zn-finger proteins that facilitate DNA binding and control transcription. Cellular Zn concentrations typically range from 100 to 1000 μM. *Escherichia coli* cells grown in either metal-deficient minimal media or metal-replete LB media contain 200 to 600 μM Zn (4, 5, 6).

Conversely, Zn ions can be toxic to cells, often due to mismetallating apo-proteins that require other metals for activity (7, 8). Thus, Zn levels in cells must be tightly regulated, but how this is done on the molecular level remains incompletely understood. In *E. coli*, intracellular Zn levels are largely controlled by transcription-factor proteins Zur and ZntR (9, 10). Under Zn-deficient conditions, Zur promotes the expression of a Zn importer which then imports additional Zn to alleviate the deficiency. Under Zn-excess conditions, Zn-bound ZntR activates transcription of ZntA, a P-type ATPase that effluxes excess Zn from the cytoplasm.

Cellular pools of nonproteinaceous *low-molecular-mass* metal coordination complexes likely function in metal ion homeostasis (11). Such pools receive metal ions from the growth media and distribute them for installation into various client apo-proteins. They have been known for decades, but their sizes (*i.e.* collective concentrations), chemical compositions, and associated trafficking reaction networks have been difficult to establish largely because of the inherent kinetic lability and modest thermodynamic stability of member complexes.

In 2001, Outten and O’Halloran investigated the size of “free or loosely-bound” Zn in *E. coli* using DNA footprinting to monitor the binding of Zur and ZntR (4). These titrations used ZnSO_4_ which generates aqueous Zn ions in solution. The binding was so tight that they also included the chelator TPEN which has a known thermodynamic Zn binding constant. The resulting more gradually changing titration curves could then be used to calculate the constants associated with the dissociation of aqueous Zn from those transcription factors. Midway activation of ZntR (*i.e.* when [apo-ZntR] = [holo-ZntR]) occurred when [Zn-TPEN] was 16.6 μM, which allowed them to calculate a dissociation constant *K*_*D*_ for ZntR of 1.15 × 10^-15^ M. That *K*_*D*_ also equals the aqueous Zn concentration under the same midway condition. A similar analysis yielded *K*_*D*_ = 9.6 × 10^-17^ M for Zur binding. They concluded that cells homeostatically regulate free or loosely bound Zn between those concentration limits. The volume of an *E. coli* cell (1.8 × 10^-15^ L) implies that cells contain, on average, seven orders of magnitude less than 1 “free” Zn ion per cell. This suggested that under normal growth conditions, “there is no persistent pool of free Zn(II) ions in the cytoplasm”; rationalized as being due to an abundance of Lewis basic sites in the cytoplasm for binding aqueous Zn. The authors suggested that Zn apo-proteins might be metallated using Zn metallochaperones, analogous to those used in copper trafficking. However, only one Zn metallochaperone family, Zngl GTPases, has been identified (12). In *E. coli*, a member of this family, YjiA, helps mobilize Zn under Zn-deficient conditions.

Numerous subsequent studies using fluorescence-based chelator probes reported picomolar concentrations of “free” or “labile” Zn in the cytoplasm of various cells, 3 to 5 orders of magnitude higher than reported by Outten and O’Halloran. Wang *et al.* fused carbonic anhydrase with a red fluorescent protein to create genetically-encoded FRET-based zinc sensors (13). After calibrating these sensors *in vitro*, they installed them into *E coli* cells. From the resulting FRET fluorescence, they calculated an intracellular “*exchangeable”* zinc concentration of 20 pM. Similar concentrations have been obtained by other investigators using a variety of cells. Krezel and Maret reported that erythrocytes, neuroblastoma cells, adenocarcinoma cells, and cardiomyocytes contain 20 to 1000 pM of “free” Zn (14), later defined as the “pool of non-protein bound zinc” (15). Much of the large Zn-binding capacity observed in their cell extracts was due to apo-metallothioneins that sequestered Zn. Using genetically encoded FRET sensors, Vinkenborg *et al.* found that cytoplasmic “free” Zn was buffered at 400 pM, high enough to metallate Zn client proteins which typically have *K*_*D*_ values between 1 to 10 pM (16). Aper *et al.* used bioluminescence Resonance Energy Transfer probes to quantify the “free” Zn in the cytoplasm of HeLa cells, obtaining a concentration of 92 to 281 pM (17). Palmer and coworkers used FRET sensors to determine that the labile Zn pool concentrations in a variety of cells were in the 100s of pM range (18, 19, 20). These concentrations, when contrasted with the six or seven orders-of-magnitude higher concentrations of cellular Zn, imply that cells contain extraordinary Zn homeostatic regulatory mechanisms that allow high concentrations of Zn to be imported but also earmark virtually *all* such metal ions for installation into proteins.

We are developing chromatographic methods to examine low-mass metal pools in cell extracts. Towards this end, we have installed a liquid chromatograph in an anaerobic refrigerated glove box and interfaced it to an inline ICP-MS for real-time metal detection of column eluate. Using this LC-ICP-MS system, low-mass Ni, Fe, Cu, and Zn pools have been investigated (21, 22, 23, 24, 25). The two approaches are complementary. Chelator probes can be added to undisrupted intact cells whereas chromatography-based investigations require cell lysis, raising the possibility of generating artifacts. On the other hand, chelator probes destroy the metal complexes of interest during detection and they perturb cellular metal ion homeostasis. Chromatography-based methods offer the *potential* to isolate and identify low-mass complexes by downstream bioanalytical methods such as ESI-MS. We emphasize *potential* because there are many challenges, mostly stemming from the inherent lability of such complexes.

In a preliminary study of the low-mass pool in *E. coli*, Brawley and Lindahl isolated cytoplasm from cells grown on 0 and 100 μM Zn and then passed them through a 3-kDa cutoff membrane to generate *flow-through solutions* (FTSs) (25). Unsupplemented cells contained 400 ± 200 μM Zn, isolated cytoplasm contained 200 ± 100 μM Zn, and FTS contained 13 ± 3 μM Zn. Their LC-ICP-MS chromatograms indicated 4 to 6 Zn peaks in the low-mass region. However, none were identified.

In this article, we more systematically characterized the low-mass Zn pool of *E. coli*, which ends up being different from the *labile* Zn pool. We reproducibly detected several low-mass zinc complexes in the cytoplasm of these cells at collective concentrations that were 3 to 5 orders of magnitude higher than reported pM concentrations, and 7 to 9 orders of magnitude higher than reported fM concentrations. ESI-MS was used to identify two Zn-glutathione complexes as dominant members of the pool under Zn-replete conditions.

## Results

### Zinc in isolated cytoplasm and FTSs

The goal of this study was to better characterize the endogenous low-mass Zn pool in lysates of *E. coli* cells grown in minimal media. Zinc toxicity in these cells is associated with the disruption of Fe_4_S_4_ clusters and the inhibition of iron-sulfur-cluster biogenesis (7, 8). Previous studies suggest that some bound Zn is released during cell lysis, so efforts were taken to minimize this problem using a gentle lysis procedure (25). The strain used then and now contains holin and endolysin genes. The expressed proteins generate pores in the outer membrane which cause lysis. These genes are expressed merely by thawing frozen cells, which allows unusually gentle lysis. Although the effect of Zn lability cannot be assessed directly by Mössbauer spectroscopy, we used it to minimize Zn (and iron-sulfur cluster) degradation upon cell lysis (Fig. S1), which was improved relative to earlier approaches (22).

Using this procedure for all batches investigated, the cytoplasm was isolated from cells grown in minimal medium supplemented with 0, 10, and 100 μM Zn(acetate)_2_. The medium without supplementation contained 0.2 μM Zn, so “0” supplementation does not mean that the medium was devoid of Zn. Some samples were passed through a 0.2 μm filter, such that both proteins and low-mass Zn complexes were present in filtrates. These are referred to as *isolated cytoplasm* and abbreviated *Cyt0*, *Cyt10*, and *Cyt100*. In other experiments, samples were passed through a 3 kDa-cutoff filter to generate flow-through solutions called *FTS0*, *FTS10*, and *FTS100*.

The Zn concentrations detected using whole cells (Table 1) varied from 400 μM in cells grown on unsupplemented media to 8500 μM in cells grown on media supplemented with 100 μM Zn(acetate)_2_. The latter concentration was higher than expected, but similar concentrations were obtained in three independent batches. In such samples, we suspected that most of the detected Zn was non-specifically bound to the outer membrane of the cells. EDTA, which is typically used to wash excess metals from cells during harvesting, was not included as it could bind endogenous metal pools (25) which could have confused the analysis. Zn concentrations in Cyt100 and FTS100 solutions were also higher than expected. The large difference in Zn concentrations between whole cells and isolated cytoplasm suggested that much of the Zn that had adhered to whole cells was lost in membrane fractions during cytoplasm isolation. The 5- to 300-fold decline in Zn when isolated cytoplasm was 3 kDa filtered to generate FTSs implies that 85% - 99% of the Zn species in isolated cytoplasm are Zn-binding proteins. We confirm those percentages below.

**Table 1:** Average zinc concentrations of whole cells, isolated cytoplasm, and FTSs

Fraction →Media suppl. ↓	Whole Cell (μM)	Isolated cytoplasm (μM)	FTS (μM)
0 μM Zn	400 ± 100	100 ± 60	0.6 ± 0.1
10 μM Zn	480 ± 280	170 ± 10	10 ± 6
60 μM Zn	4100 ± 600	660 ± 120	ND
100 μM Zn	8500 ± 680	1200 ± 600	300 ± 50
100 μM Zn; EDTA wash	1300 ± 500	ND	ND

Total Zn concentrations obtained by ICP-MS as described in Experimental Procedures. Values are 1.35× higher than those for samples used for LC-ICP-MS. Each entry is the averages of n = 3 to 5 independent replicates per sample. ND, not determined.

Prompted by a reviewer comment, we isolated Cyt100 from two batches of cells that had been washed twice with 1 mM EDTA followed twice with high purity, trace-metal-free, double distilled water (HPW). The whole-cell Zn concentration of these cells was substantially diminished (Table 1), supporting our explanation. Still to be explained are the 6 to 11 orders-of-magnitude higher concentrations of Zn determined for our FTSs compared to previous estimates of “free” or “labile” Zn. To address this issue, we performed LC-ICP-MS.

### LC-ICP-MS of cytoplasm

Solutions of cytoplasm, FTS, and various standards were injected onto a low-mass-resolving size-exclusion column. Eluates were monitored at 280 nm using a diode-array detector and for Zn and S by ICP-MS. Peaks in resulting chromatograms (a.k.a. *traces*) were fitted in terms of elution volume, intensity (counts), and full width at half-maximum (FWHM). Peaks were named according to the element detected and the elution volume; *e.g.* Zn16.8 indicates a Zn peak that eluted at 16.8 ml. Peaks with elution volumes that differed by more than ± 0.1 ml of another peak (*e.g.* Zn16.7 or Zn16.9) were assumed to arise from the same species.

Aqueous Zn ions, as found in solutions of Zn(acetate)_2_ and coordinated exclusively by rapidly exchanging waters and hydroxide ions, did not elute from the column but were adsorbed onto it (Fig. S2). The extent of adsorption was likely determined by an equilibrium involving aqueous Zn ions and unknown Lewis-basic sites on the column. A similar though less severe problem occurred with non-aqueous Zn coordination complexes. In these cases, the extent of adsorption varied inversely with the stability of the complex, with more stable complexes, such as Zn bound to chelators, adsorbing less. We could not eliminate this adsorption problem entirely but quantified it by dividing the overall Zn intensity in the chromatogram by that obtained when the same sample was injected onto a “ghost column” composed of Peek tubing in place of the actual column. Between 10% and 90% of the Zn in our *E. coli* samples eluted.

A related problem was that a small portion of the adsorbed metal ions *desorbed* upon subsequent injection of solutions containing Lewis-basic groups. We minimized this by cleaning the column with a chelator solution between runs and by using large sample volumes and high concentrations. These practices along with the reproducibility obtained by repeat injections provided confidence that observed Zn peaks reflected endogenous Zn species in the cytoplasm of *E coli* cells.

**Figure 1:**
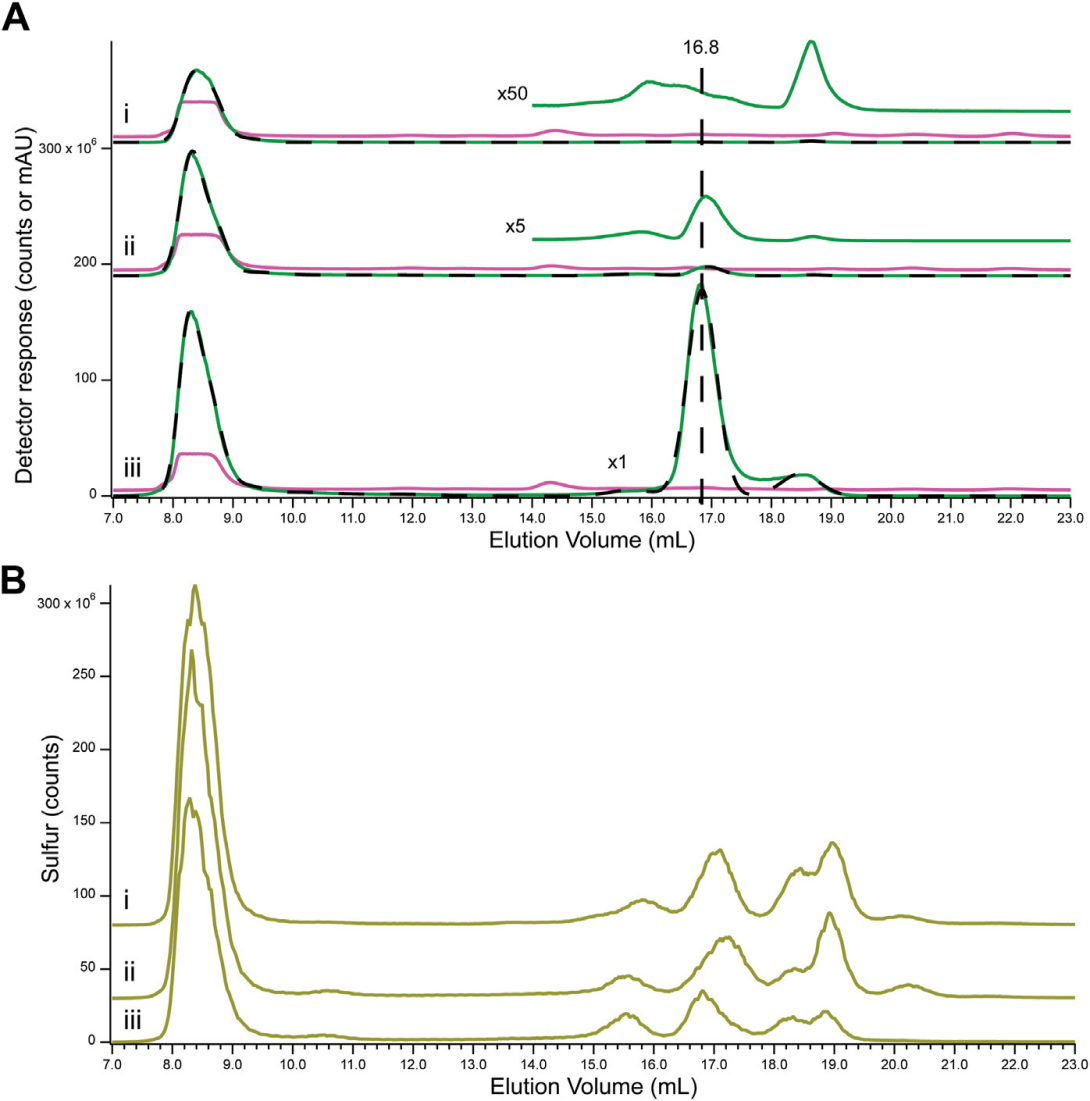
**LC-ICP-MS chromatograms of cytoplasm isolated from cells grown with different levels of zinc supplementations.***A*, Zn (*green*), A280 (*pink*), and simulations (*black dashed*). *B*, S (*yellow*). (*i*), Cyt0; (*ii*), Cyt10; (*iii*), Cyt100. Each chromatogram is the average of three independent batches. Multiplication factors: (*i*), Zn 1 × , zoomed 50 × , ^32^S 1 × ; A280, 100,00 × ; (*ii*), Zn 1 × , zoomed Zn 5 × ; ^32^S 1 × , A280 10,000 × ; (*iii*), Zn 1 × , ^32^S 1 × , A280 100,00 × . The same color coding was used for all figures.

Cyt0, Cyt10, and Cyt100 Zn traces (Fig. 1*A*, green lines *i*–*iii*) were fitted with nine peaks, including Zn8.2, Zn8.5, Zn9.2, Zn10.2, Zn15.7, Zn16.2, Zn16.8, Zn17.1, and Zn18.6. The first four peaks absorbed at 280 nm (Fig. 1*A*, pink lines). This along with the absence of those peaks in corresponding FTS traces indicated that they arose from Zn-bound proteins. The number of Zn proteins contributing to these peaks could not be established, as proteins were poorly resolved on the column used. The remaining peaks in the series (Zn15.7 – Zn18.6), were collectively defined as the *low-mass Zn pool* which we initially presumed to be identical to the *labile* Zn pool.

Once each peak was fitted, the total intensity associated with Zn in the trace was calculated by summing individual peak intensities. Relative to that of Cyt0, total Zn intensities of Cyt10 and Cyt100 were 1.7- and 5.3-fold higher (Fig. 1*A*, *i*–*iii*). These fold-increases should be compared to the measured Zn concentrations in Cyt10 and Cyt100, relative to Cyt0 in Table 1. The ratio of total chromatograph Zn intensities to corresponding Zn concentrations afforded a calibration factor (counts per μM Zn) (Table S1).

About 97% of Zn in Cyt0 cytoplasm was bound to proteins while the remainder was low-mass nonproteinaceous Zn coordination complexes. Thus, the vast majority of Zn that enters Zn-limited cells is ultimately trafficked to Zn apo-proteins rather than remain in the low-mass pool. However, as cells became increasingly Zn-sufficient (Cyt10 → Cyt100), an increasing fraction coordinated to non-proteinaceous ligands. If *homeostasis* of Zn refers to the degree to which cellular Zn concentrations remain unchanged as cells are exposed to increasing concentrations of nutrient Zn, then *Zn homeostasis in E. coli cells is far less effective than we expected*; the concentration of cytoplasmic Zn increased ca. 5-fold as media Zn concentrations increased ca. 500 fold. For perfect homeostasis, the ratio 5/500 would equal zero.

We also collected and analyzed corresponding sulfur chromatograms, and used them to estimate injection reproducibility, intensity variations, and possible association with certain Zn peaks. A multitude of S peaks were detected (Fig. 1*B*) including one group between 8 to 10 ml which reflected S-bound proteins, and a second group between 15 to 24 ml which reflected low-mass sulfur species. The fractions of total S intensity due to these two groups were largely invariant across the series, with S-bound proteins representing 60% of total S and low-mass sulfur species representing 40%. The intensities and elution volumes of some low-mass peaks changed modestly, the most interesting being a shift of S17.2 → S16.7 across the series. We show below that these peaks arise from GSH or its derivatives.

The composition of the low-mass Zn pool also changed across the series Cyt0 → Cyt10 → Cyt100. Most dramatic was the development of the Zn16.8 peak (Fig. 1*A*, *iii*). This peak, shown below to arise from Zn-GSH, was absent in Cyt0 (trace *i*), modestly present in Cyt10 (trace *ii*), and dominant in Cyt100 (trace *iii*). In contrast, Zn15.7 and Zn18.6 intensities were similar across the series.

**Figure 2:**
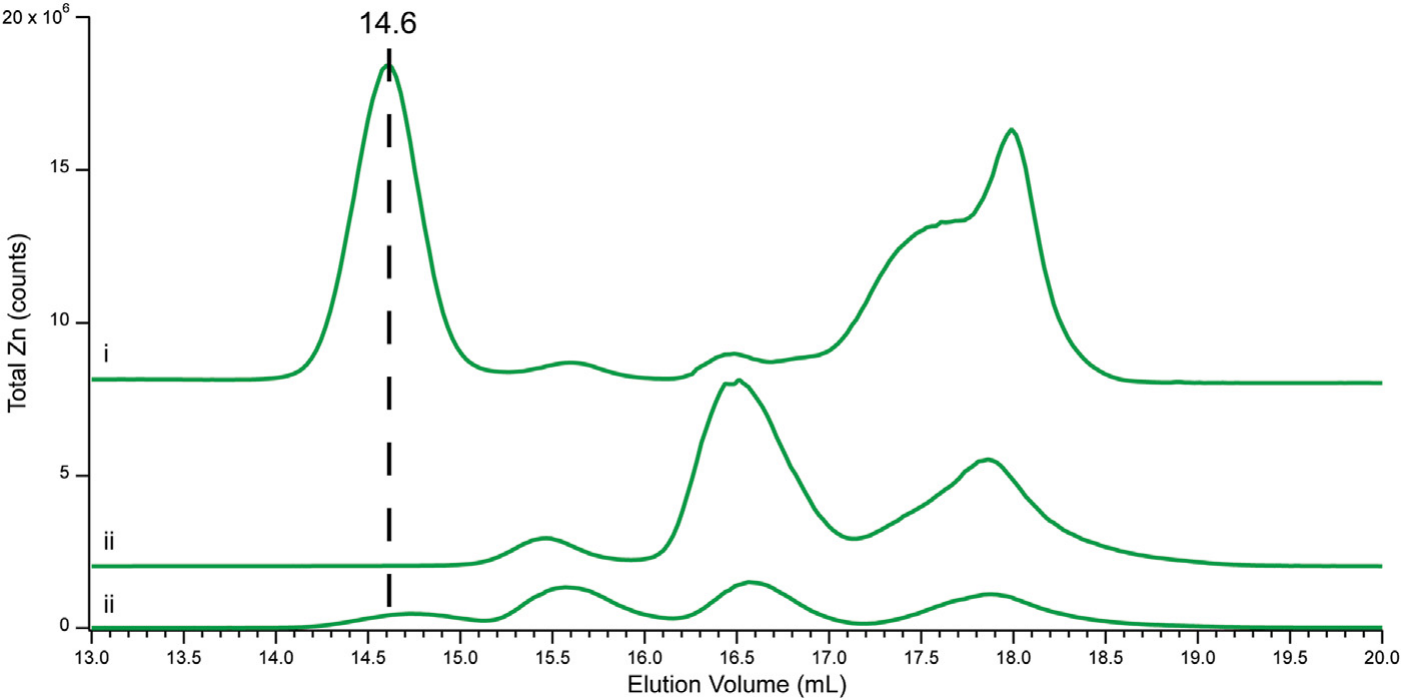
**Effects of EDTA washing and spiking.** Zn traces for (*i*) Cyt100 washed 2× with 1 mM EDTA solution prior to cell lysis; (*ii*), FTS0; (*iii*), FTS0 spiked with 5 mM EDTA.

We also examined the two Cyt100 samples from cells that had been EDTA-washed during harvesting (Fig. 2, *i*). About 82% of total Zn intensity in both traces was associated with proteins, 7% with a Zn-EDTA complex, and 11% with other low-mass Zn complexes. The elution volumes and relative concentrations of these complexes differed compared to Cyt100 from cells that were not washed with EDTA, especially the absence of Zn16.8 (compare Fig. 2*i* to Fig. 1, *iii*). Some EDTA must have remained despite subsequent washings with HPW, and the residual must have perturbed the low-mass pool, *e.g.* “ripping” Zn away from GSH. Clearly, the low-mass Zn species in the cytoplasm are *labile* to EDTA chelation.

### Stability of Zn proteins and complexes with time

We routinely ran cytoplasm samples immediately after isolating them, but the time for which samples were queued in our refrigerated N_2_-atmosphere glove box could be many hours. To probe the stability of Zn-bound proteins and complexes, aliquots of the same Cyt0 sample were injected daily into our ICP-MS system over a 72-h period. Most Zn species in isolated cytoplasm were stable, but a few (*e.g.* Zn17.1) were not (Fig. S3, *A* and *B*). We normalized the total Zn intensities in the four chromatograms to sulfur intensities. However, the latter also showed some day-to-day variations (7% coefficient of variation). A sulfur normalization factor was calculated as the total S intensity for each day divided by the average total S intensity for the series. The resulting normalized total Zn intensities declined just 1.7% per day on average. Intensities likely declined as aqueous Zn ions were released and then adsorbed onto the column. Seventy percent of the overall decline was due to Zn-bound proteins; and 30% to Zn complexes. However, since Zn complexes represented only ∼ 3% of the total Zn, these complexes appear to be an order of magnitude *less* stable than Zn proteins. These results were supported by a similar study conducted using a Zn-GSH standard for which no systematic decline in intensity was observed.

### TPEN titrations

**Figure 3:**
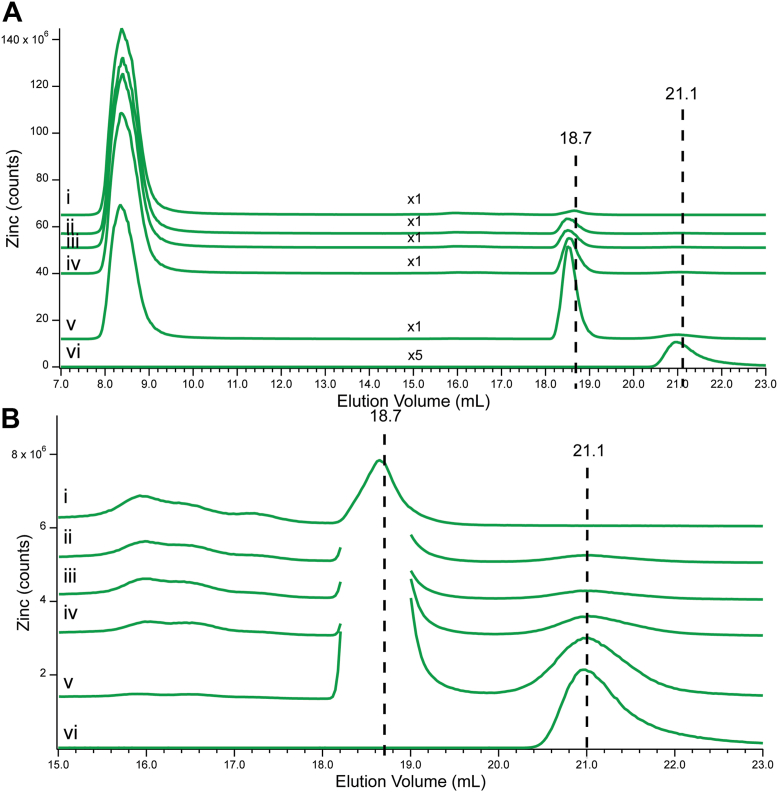
**Effect of spiking Cyt0 with TPEN.** Total Zn intensities were normalized to ^32^S intensities. *PanelA*: (*i*), Cyt0; (*ii*–*v*), same as (*i*) but with the following final concentrations of TPEN added and adjusted for dilution: (*ii*) 5; (*iii*) 10; (*iv*) 20; (*v*) 50 μM; (*vi*), 2 μM Zn(acetate)_2_ plus 5 μM TPEN in 20 mM ABC buffer pH 7.5. Multiplication factors as indicated. *PanelB*: Same as in *A* except that the low-mass region has been amplified. Multiplication factors are all ×1.

The lability of Zn species in Cyt0, Cyt10, and Cyt100 was further evaluated by adding 0, 5, 10, 20, and 50 μM (final concentrations) of TPEN, and analyzing changes in Zn intensities by LC-ICP-MS. TPEN was selected, as this chelator is commonly used to obtain the background signal in fluorescent probe studies. The total Zn intensity of Cyt0 declined 19% during the titration (Fig. 3*A*, *i*–*v*). Most individual Zn peaks also declined, but Zn18.7 and Zn21.1 increased. An expanded view is shown in Panel B. Zn21.1 developed with the first addition of TPEN, and its intensity increased with each subsequent addition. The Zn-TPEN standard (Fig. 3, *vi*) eluted at the same volume, and so we assigned Zn21.1 to the complex. On the other hand, Zn18.7 was observed in the absence of TPEN, and Zn-TPEN did not elute at this volume. Perhaps a different Zn-TPEN complex, formed in the cytoplasm but not in buffer, comigrated with the endogenous Zn18.7 species.

As mentioned earlier, the tendency of a Zn complex to adsorb onto the column varied inversely with its stability such that Zn bound to chelators did not adsorb significantly. Zn-TPEN fits into this category. Thus, we expected that the total Zn intensity for each chromatogram in the TPEN titration would remain unchanged because any Zn that was displaced from weaker-binding species would be replaced stoichiometrically with the Zn-TPEN complex. Thus, the observed overall decline in Zn intensity during the titration was unexpected. S traces were used to normalize the intensities of each Zn chromatogram. This reduced the overall Zn intensity decline to 3%, which we considered acceptable. Surprisingly, analysis of these normalized traces revealed that about 94% of the displaced Zn was from Zn-bound proteins; and only 6% from Zn-bound complexes. However, 96% of the total Zn intensity in the Cyt0 sample prior to adding TPEN was due to proteins; only 4% to Zn complexes. *We conclude that TPEN displaces Zn from both proteins and Zn complexes with about equal propensity*. It is certainly *not the case* that proteins are immune from TPEN chelation. These results confirm what has been suspected by many researchers in the field, namely that *the labile Z. pool is not identical to the low-mass pool.*

In the TPEN titration of Cyt10 (Figs. S4*A*), 83% of the Zn was removed from proteins. Three Zn peaks developed as TPEN was added, including Zn18.8, Zn19.3, and Zn21.6. The first and last of these likely reflected the same peaks as Zn18.7 and Zn21.1 in the Cyt0 titration. The results of the TPEN titration of Cyt100 (Fig. S4*B*) were similar, except that total Zn counts declined 14% after sulfur normalization. Half of the total Zn loss was due to Zn-bound proteins; the other half was due almost exclusively to the loss of Zn16.8.

### Spiking cytoplasm with aqueous ^67^Zn

**Figure 4:**
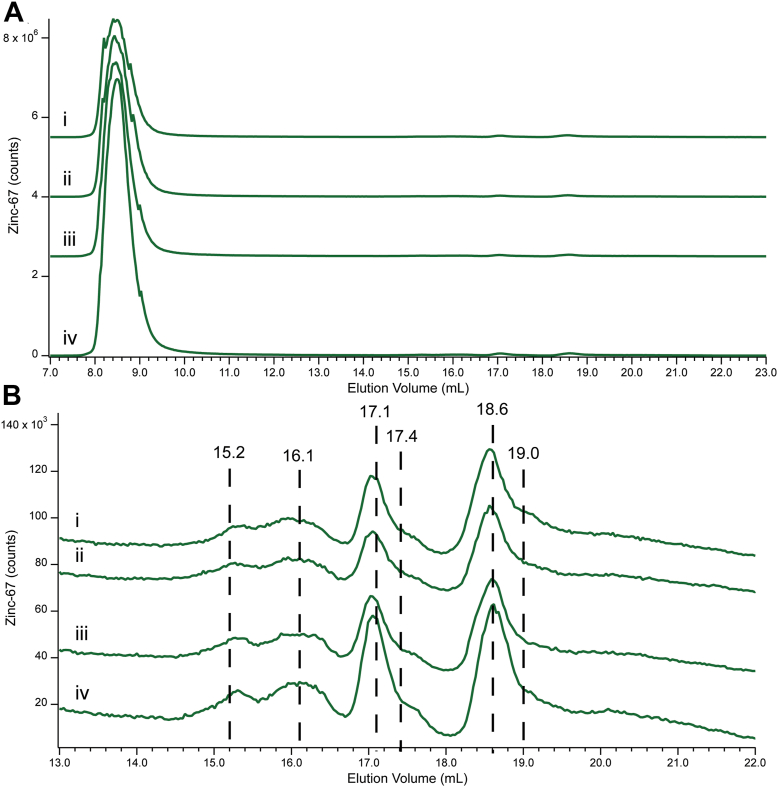
**Effects of spiking Cyt0 with aqueous ^67^Zn.***Panel A*: (*i*), Cytoplasm isolated from unsupplemented cells; (*ii*–*iv*), same as (*i*) but with the following final concentrations of ^67^Zn added, and adjusted for dilution: (*ii*), 2; (*iii*), 5; (*iv*), 10 μM. *Panel B*: Same as *Panel A* but highlighting the low-mass species. ^67^Zn counts were normalized to sulfur intensities.

We next evaluated the ability of cytoplasm to accommodate additional aqueous Zn. A Cyt0 sample was divided into four aliquots. Aliquots were treated with 0, 2, 5, or 10 μM ^67^Zn(acetate)_2_ (final concentrations), analyzed by LC-ICP-MS, and fitted as described above. Cyt0 was prepared from cells grown on natural-abundance Zn, so the use of a single isotope of aqueous Zn in the titration allowed the fate of the added Zn to be monitored. Non-^67^Zn isotopes served as a control. Overall ^67^Zn intensity increased approximately linearly with added ^67^Zn (Fig. 4), whereas the intensity of other isotopes didn’t increase (Appendix Spreadsheets). Prior to adding ^67^Zn, 94% of the total ^67^Zn intensity of the Cyt0 trace was due to Zn-bound proteins (Fig. 4*A*). Ninety-five percent of total growth arose from apo-proteins binding aqueous ^67^Zn; 2% from Zn-binding low-mass ligands. Thus, high concentrations of Zn-binding apo-proteins and unbound Zn-binding ligands must have been present in the Cyt0 sample. Both groups bound added aqueous ^67^Zn approximately in proportion to their relative amounts (of corresponding Zn-bound forms) before the addition.

### Low-mass Zn pools in FTSs

**Figure 5:**
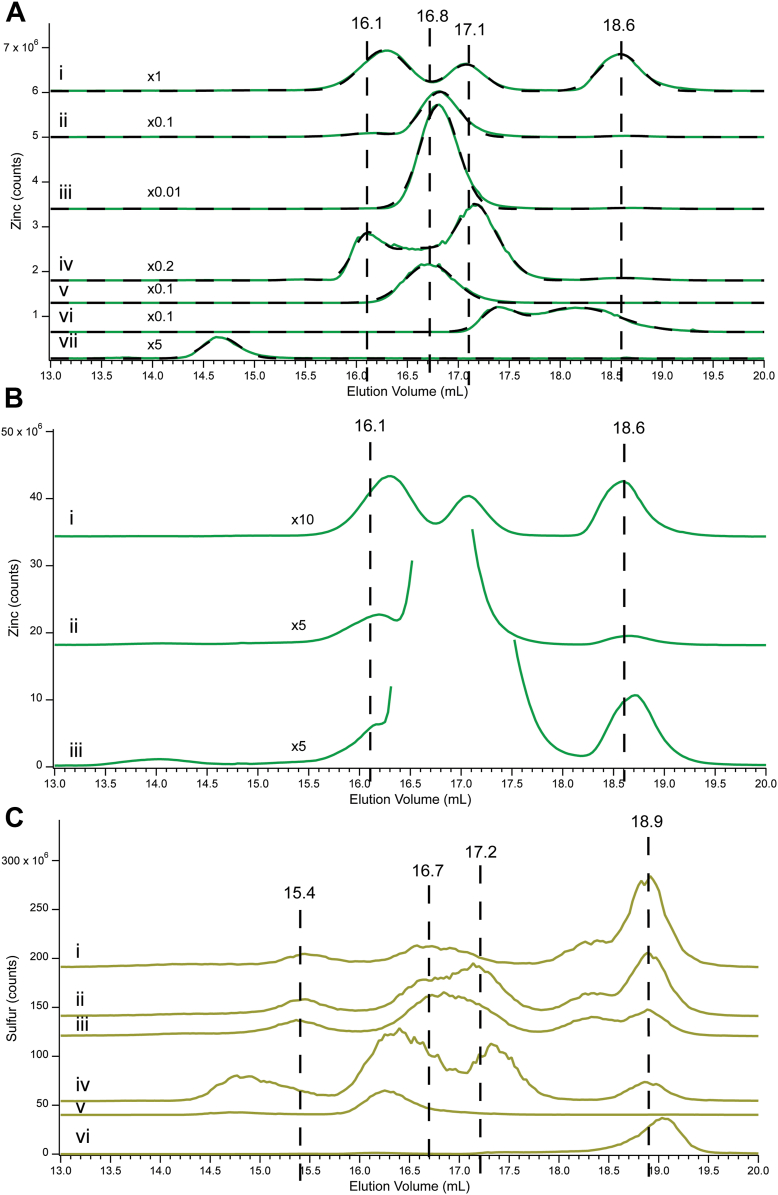
**Low-Mass Zn pools FTSs and Zn standards.***PanelsA*–*C*: (*i*), FTS0; (*ii*), FTS10; (*iii*), FTS100 (each average of n = 3). Panel *A*: (*iv*), pFTS with a final concentration of 2 μM Zn(acetate)_2_, 20 mM ABC pH 7.5; (*v*–*vii*), standards prepared in 20 mM ABC pH 7.5 with final concentrations of 2 μM Zn(acetate)_2_ and 1 mM of: (*v*), GSH; (*vi*), cysteine; (*vii*), citrate. Multiplication factors: (*i*), total Zn × 1; (*ii*), total Zn × 0.1; (*iii*), total Zn × 0.01; (*iv*), total Zn × 0.2; (*v* -*vi*), total Zn × 0.1; (*vii*), total Zn × 5. *PanelB*: (*i*) total Zn × 10; (*ii*–*iii*) total Zn × 5. Panel (*C*): (*v*–*vi*), standards prepared in 20 mM ABC pH 7.5 with final concentrations of 2 μM Zn(acetate)_2_ and 1 mM of: (*v*), GSH (*vi*), cysteine. Multiplication factors: (*i*–*vi*), ^32^S × 1.

We next focused on FTS solutions, which exclusively contained low-mass Zn complexes. FTS0, FTS10, and FTS100 were analyzed by LC-ICP-MS. In all cases, four major low-mass Zn peaks were observed. These included Zn16.1 (39% → 0%), Zn16.8 (0 → 93%), Zn17.1 (21% → 5%), and Zn18.6 (31% → 1%) (Fig. 5*A*, *i* → *iii*), where percentage changes in intensity across the series are indicated in the parentheses. In absolute terms, overall Zn intensities increased across the series, and all peak intensities increased except for Zn16.1. Relative to FTS0, the intensities of FTS10 and FTS100 were 1.4- and 15-fold greater.

In FTS0, two peaks (Zn16.1 and Zn18.6) accounted for 84% of total Zn intensity. In FTS10, Zn16.1 and Zn16.8 collectively accounted for 93% of the total, and in FTS100, Zn16.8 alone accounted for 97% of the total average intensity (Fig. 5, *A* and *B*).

Corresponding sulfur traces exhibited low-mass peaks S15.4, S16.7, S17.2, and S18.9 (Fig. 5*C*) similar to the peaks observed in Cyt0 → Cyt100 (Fig. 1*C*), including noted shifting between S17.2 and S16.7 across the series.

### Spiking FTSs with aqueous Zn

**Figure 6:**
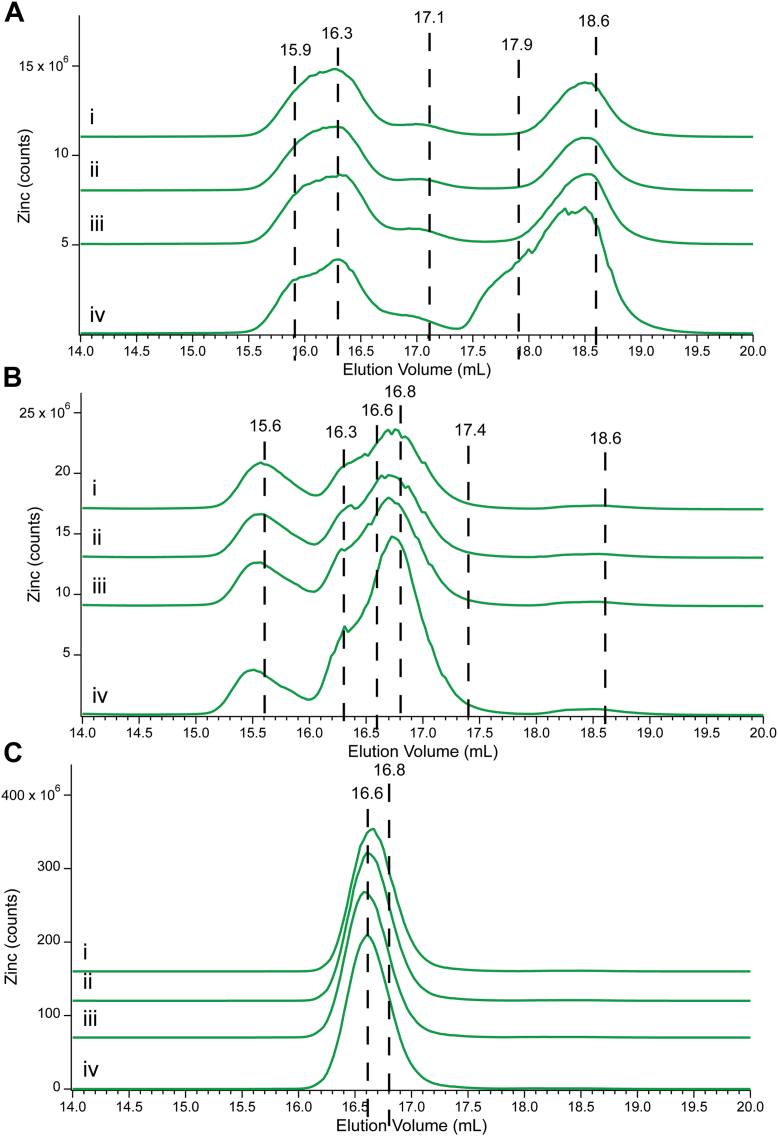
**Spiking of FTSs with Zn(acetate)_2_**. Total Zn intensities have been normalized to S intensities. *Panel A*: (*i*), FTS0; (*ii*–*iv*), same as (*i*) but with the following final concentrations of Zn(acetate)_2_ added and adjusted for dilution: (*ii*), 2; (*iii*), 5; (*iv*), 10 μM. Multiplication factors (*i* - *iv*): total Zn × 1. *Panel B*: (*i*), FTS10; (*ii*–*v*), same as (*i*) but with the following final concentrations of Zn(acetate)_2_ added and adjusted for dilution: (*ii*), 2; (*iii*), 5; (*iv*), 10 μM. Multiplication factors: (*i*–*iv*), total Zn × 1. *Panel C*: (*i*), FTS100; (*ii*–*v*), same as (*i*) but with the following final concentrations of Zn(acetate)_2_ added and adjusted for dilution: (*ii*), 2; (*iii*), 5; (*iv*), 10 μM. Multiplication factors: total Zn × 1.

We wanted to evaluate the capacity of FTSs to bind added aqueous Zn. To do this, we added 0, 2, 5, and 10 μM Zn(acetate)_2_ to freshly prepared FTS0, FTS10 and FTS100. Before this, FTS0 exhibited four major peaks, including Zn15.9 (16%), Zn16.3 (40%), Zn17.1 (7%) and Zn18.6 (36%) (Fig. 6*A*). Unexpectedly, none of these intensities increased until 5 and 10 μM Zn(acetate)_2_ were added. Then, Zn18.6 and a new peak at Zn17.9 mainly increased intensities.

FTS10 exhibited five major peaks (Fig. 6*B*), including the three observed previously (Zn15.6 (21%), Zn16.3 (12%), and Zn16.8 (60%)) and two minor peaks Zn17.4 (2%) and Zn18.6 (3%). All peaks increased as aqueous Zn was added, but Zn16.3 and Zn16.8 were collectively responsible for 91% of the growth.

Prior to the addition, FTS100 (Fig. 6*C*) exhibited two major peaks, Zn16.6 (61%), Zn16.8 (36%), and two minor ones, Zn17.4 (1%), and Zn18.2 (1%). Virtually all of the growth was with Zn16.6, unexpectedly countered by a *decrease* of Zn16.8. We can’t explain this, but it was as though aqueous Zn contributed to the growth of Zn16.6 as Zn16.8 also converted to Zn16.6.

We also evaluated the lability of the low-mass pool by adding 5 μM EDTA to two independent FTS0 samples. As shown in Figure 2 (*ii* before and *iii* after addition), this chelator perturbed the low-mass Zn pool, especially by diminishing the intensity of Zn16.5 and giving rise to Zn14.6 (due to Zn-EDTA). The corresponding sulfur peaks were unchanged, suggesting that EDTA removed Zn from GSH and other low-mass Zn complexes. Overall, 5 μM EDTA lowered the overall Zn intensity of the low-mass Zn pool by 65% (excluding the Zn-EDTA complex, which developed).

**Figure 7:**
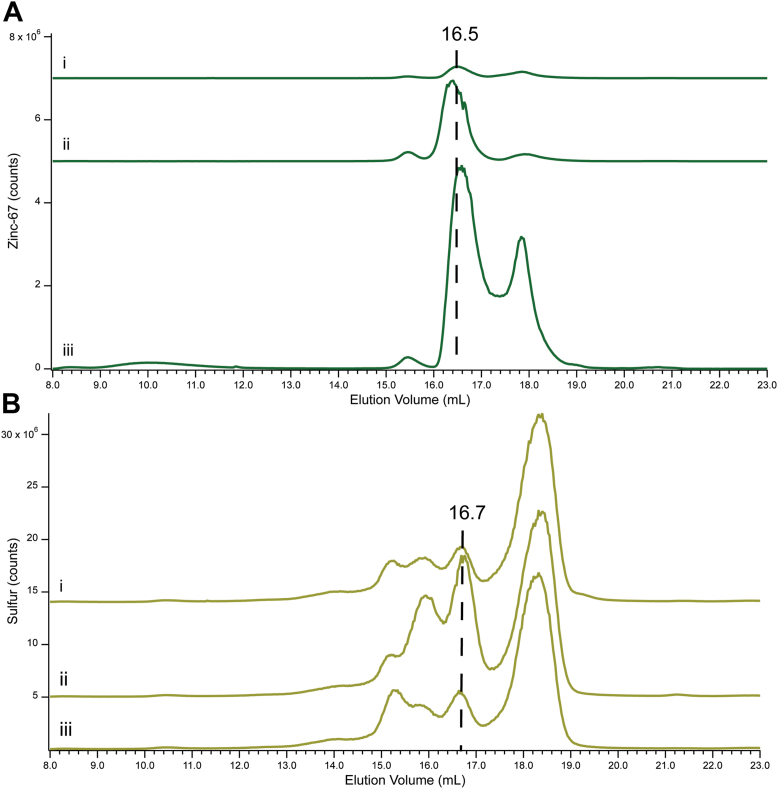
**LC-ICP-MS of FTS0 before and after spiking with aqueous Zn or GSH.***Panel A*, Zn detection: (i), FTS0; (ii), same as (i) but after adding 1 mM GSH (final concentration); (III), same as (i) but after adding 100 μM ^67^Zn(acetate)_2_. *Panel B*, same as *Panel A* but S detection.

To determine the effect on the Zn-GSH peak (Zn16.5), we spiked FTS0 with 1.0 mM GSH and separately with 100 μM of ^67^Zn(acetate)_2_. The addition of GSH (Fig. 7*A*, trace *i versus ii*) increased the intensity of Zn16.5 and the corresponding S16.7 (Fig. 7*B*, trace *ii* vs *i*). Likewise adding 100 μM ^67^Zn(acetate)_2_ increased the intensity of Zn16.5 (and Zn17.8) (Fig. 7*A*, trace *iii*). We conclude that there is a dynamic equilibrium with the approximate form {Zn + GSH ⇄ Zn(GSH)} in *E. coli* cytoplasm such that adding either reactant shifts the equilibrium towards complexation.

These Zn-spiking experiments demonstrate that cytoplasm devoid of proteins can bind at least 10 μM or more of added additional aqueous Zn. The ligands that coordinate the added Zn are diverse; once coordinated, they afforded 3 to 5 Zn complexes. The ligands used in Zn-limited cytoplasm differed from those used in Zn-replete cells, with Zn16.3 to Zn16.8 mostly affected. The presence of these Zn-binding ligands in *E. coli* cytoplasm confirms the previous conclusion of Outten and O'Halloran that cells are devoid of aqueous Zn.

### Zn calibration factor

We calculated a Zn calibration factor from the average of eight experiments performed in this study (Table S1). This factor had units of ICP-MS detector counts per μM Zn concentration present in the 100 μl samples injected into the ICP-MS. Counts were determined from integrated peak areas. Such factors are crude approximations because of daily variability in tuning the ICP-MS and variations in the proportion of Zn in a sample that is adsorbed onto the column. Of the 10 calculated factors considered, two were excluded (one high and one low) because they differed from the average of the other eight by more than 2 standard deviations. The resulting average calibration factor (600,000 ± 130,000 counts per μM Zn) was then used to estimate the concentration of total Zn in various samples or for individual Zn species in a sample (Table S2). These estimates have large uncertainties, but they suggest the following.

• Cytoplasm from cells that were not supplemented with Zn (Cyt0) contain 85 ± 15 μM Zn total, with proteins representing 80 ± 10 μM and the Low-mass Zn pool representing 5 ± 3 μM, with little due to Zn-GSH complexes.
• Cytoplasm from cells that were supplemented with 10 μM Zn (Cyt10) contains 130 ± 10 μM Zn total, with proteins representing 115 ± 10 μM and low-mass Zn pool representing 15 ± 5 μM, about evenly split between Zn-GSH and other Zn complexes.
• Cytoplasm from cells grown with 100 μM Zn supplementation (Cyt100) contains 400 ± 150 μM total Zn, with Zn proteins representing 200 ± 70 μM, Zn-GSH representing 170 ± 60 μM, and other low-mass Zn pool members representing 30 ± 15 μM Zn.

### Standard Zn coordination complexes

We could not identify all low-mass pool members but constrained possibilities by running various standard Zn complexes under similar conditions. The Zn-detected trace of the Zn-GSH standard eluted at 16.8 ml, which was within error of peaks in Cyt10, Cyt100, FTS10, and FTS100 samples with elution volumes ranging from 16.5 to 16.9 ml (Fig. 5*A*, *v*). From this, we tentatively assigned these peaks to Zn-GSH or its derivatives. The corresponding S-detected trace in the Zn-GSH standard eluted at 16.3 ml, 0.5 ml before the Zn peak. The Zn peak reflects the Zn-GSH complex (present at 2 μM), whereas the S peak reflects uncoordinated GSH (present at 1 mM). The Zn-detected trace of Zn-cysteine migrated as two partially resolved peaks with elution volumes of 17.4 ml and 18.2 ml, suggesting two forms (Fig. 5*A*, *vi*). The Zn-cysteine complex exhibited a single corresponding S19.0 peak. The S18.9 peak found in the FTS samples (Fig. 5*C*, *i–iii*) likely arose from cysteine. We tentatively assign Zn18.7 exhibited by all cytoplasm and FTSs to Zn-cysteine; however, further work is needed to establish this.

The Zn-detected trace of Zn-citrate exhibited a peak at 14.8 ml but it was unusually weak suggesting that the Zn-citrate complex is not as stable as Zn-cysteine or Zn-GSH (Fig. 5*A*, *vii*). Zn-citrate did not coelute with any of the observed species. Solutions of aqueous Zn mixed separately with ATP, histidine, aspartate, glutamate, and GSSG did not exhibit Zn peaks (Fig. S5), suggesting that these complexes are not formed under the conditions investigated.

Peaks from standards may not comigrate precisely with peaks from cytoplasm samples due to matrix differences in their environments (simple aqueous buffer vs a complex array of cytoplasmic species). Cytoplasmic Zn complexes may be composed of ligands that were not included in the standards tested, or they may include a combination of those ligands. To probe this, a solution meant to mimic the cytoplasm FTS in terms of potential ligands and concentration, termed the *pseudo-flow-through solution* (pFTS), was prepared and analyzed. This solution contained ATP, ADP, AMP, GSH, GSSG, His, Asp, Glu, Cys, phosphate and polyphosphate at concentrations similar (or anticipated to be similar) to those in *E. coli* cells (Fig. 5*A*, iv). The pFTS exhibited 6 Zn peaks with intensities > 2%, including at 16.1 ml (20%), 16.5 ml (21%), 16.9 ml (16%), 17.2 ml (28%), 17.4 ml (13%), and 18.6 ml (2.3%). These are in the same region as observed in the FTS. The corresponding S trace exhibited four major peaks, at 14.9 ml (18%), 16.4 ml (44%), 17.3 ml (30%), and 18.9 ml (8%)—even though only three sulfur-containing species were included in the pFTS, highlighting the possibility that some cytoplasm complexes might coordinate a combination of the considered ligands.

### ESI-MS of the Zn16.8 fraction collected from highly concentrated FTS

**Figure 8:**
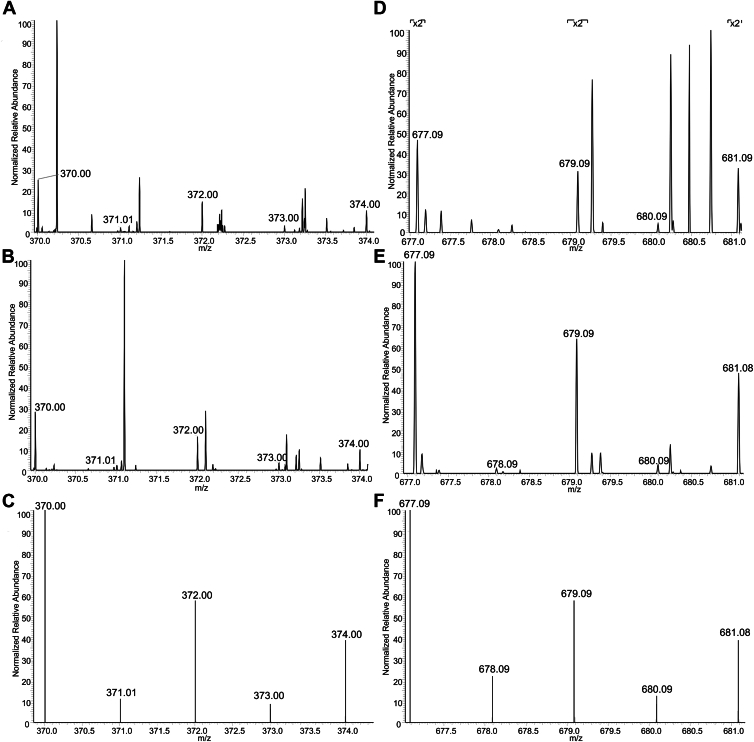
**ESI-MS of the fraction containing Zn16.8 from Cyt100**. *A*, positive mode ESI-MS singly charged spectrum corresponding to Zn(GSH)_1_ in the fraction from cytoplasm supplemented with 100 μM Zn. *B*, same as (*A*) but shows Zn(GSH)_1_ in the spectrum of the lyophilized fraction. *C*, predicted natural abundance isotopologue for Zn(GSH)_1_. *D*–*F*, same as (*A*–*C*), respectively, but corresponding to Zn(GSH)_2_.

Identifying low-mass metal pool members by ESI-MS is challenging due to the low concentration of the species of interest and the high concentrations of salts that suppress ESI-MS signals. Because the intensity of the tentatively assigned Zn-GSH peak at 16.8 ml was sufficiently large in FTS100 samples, this species held the greatest promise of being identified. To attempt this, 500 μl of FTS100 was injected into the column, and fractions containing this peak (16–17 ml) were collected. A small volume was used directly for ESI-MS while the remainder was lyophilized and resuspended in 50 μl of HPW. Both fresh and lyophilized samples were analyzed by ESI-MS. Both samples exhibited m/z peaks indicating zinc bound to 1 GSH molecule (Fig. 8, *A* and *B*). These included the full predicted isotopologue pattern at m/z = 370.00, 371.01, 372.00, 373.00, and 374.00 (Fig. 8*C*). As expected, peak intensities were higher for the lyophilized sample. Both samples also contained a species with 1 Zn bound to 2 GSH molecules (Fig. 8, *D* and *E*), exhibiting peaks at m/z = 677.09, 679.09, 680.09, and 681.08 to 681.09. The lyophilized sample again showed the full isotopic profile including m/z = 678.10.

**Figure 9:**
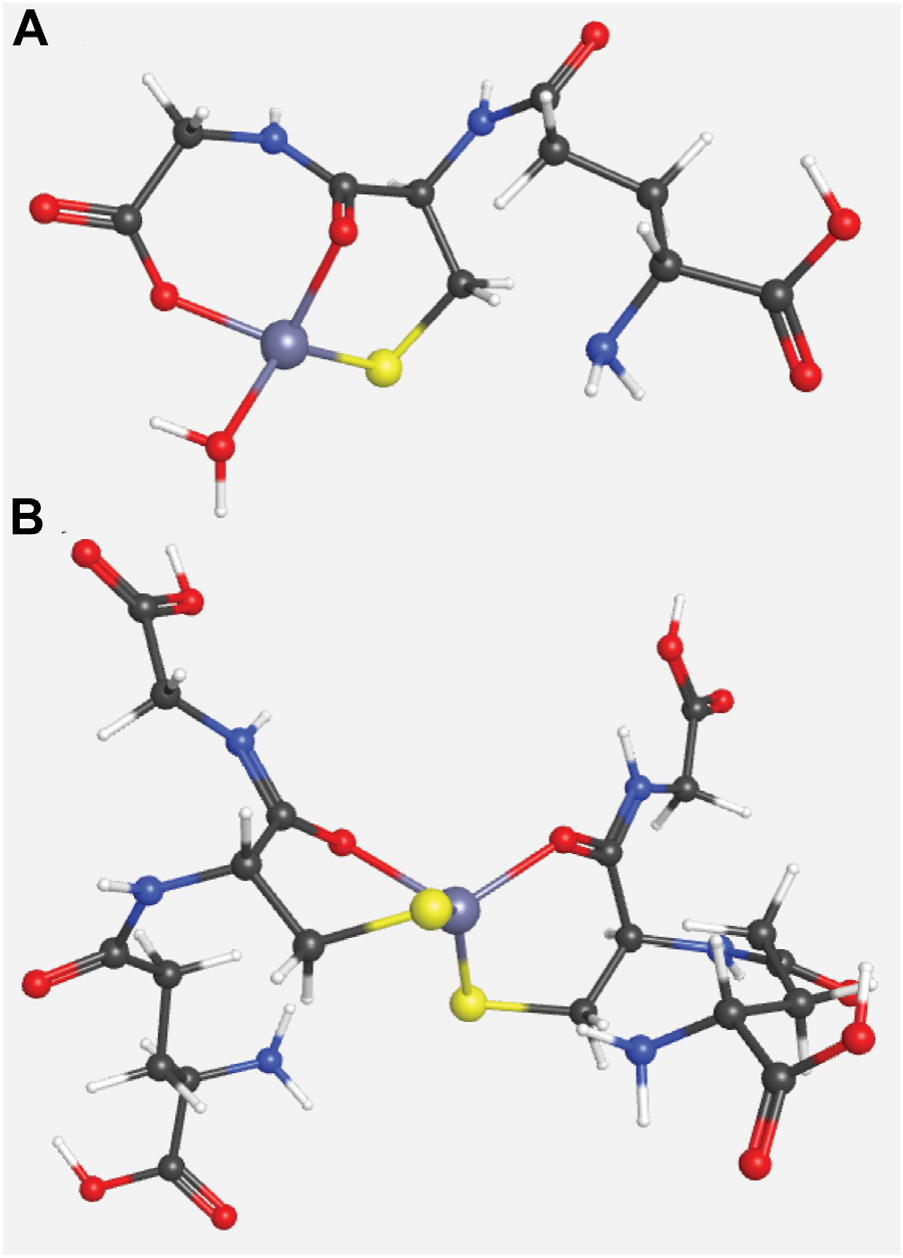
**Geometry-optimized structures for (*****A*****) Zn(GSH)_1_ and (*****B*****) Zn(GSH)_2_.** Density-functional-theory geometry optimizations in the gas phase were performed using Gaussian 16 (revision C.01) with the B3LYP functional and the 6-31G(d) basis set for all atoms. Structures based on those proposed (19).

The observed pattern indicated Zn:GSH molar ratios of 1:1 and 1:2, and no other ligands, implying that GSH chelates Zn as suggested by Krezel *et al.* (26). The DFT-optimized structure of Zn-GSH (Fig. 9*A*) includes 1 water. The water may be absent in the mass spectrum due to dehydration of the charged droplets during analysis. The corresponding DFT-optimized Zn(GSH)_2_ structure (Fig. 9*B*) is chelated by two GSH molecules using S and O donors.

### Growth in Zn-GSH LC-ICP-MS peak from FTS0 → FTS100 is mainly due to increased Zn concentration

We considered that *E. coli* cells increase the expression of genes involved for GSH biosynthesis when grown in media supplemented with high levels of Zn(acetate)_2_, but this does not seem to be the case. Using primers for *gshA*, one of the two genes required in GSH biosynthesis, we performed quantitative PCR on cells grown with increasing Zn in the media. However, the expression of that gene did not increase (Fig. S6). This is consistent with the minor shifts in the intensity of the S peak(s) assigned to GSH as Zn levels in the media increased (Figs. 1*C* and 5*C*).

## Discussion

### What are “free”, labile, and low-mass metal pools?

Such pools have been known to exist in cells for a half-century, but their molecular-level characterization remains uncertain, largely due to the inherent kinetic lability and limited thermodynamic stability of its members. Contributing to these difficulties have been the many ambiguous descriptors and synonyms for these pools, including *free*, *chelatable*, *labile*, and *exchangeable*. Capdevila *et al.* defined “free” Zn as “that fraction of total Zn not tightly bound to protein and in rapid chemical exchange among small molecules” (3) and that view seems fairly popular in the field. Here we define *free* exclusively as aqueous Zn and *low-mass* as non-proteinaceous Zn complexes coordinated to at least one non-aqueous ligand. The terms *labile, chelatable, exchangeable*, and *loosely bound* are operational definitions that might include aqueous Zn, low-mass Zn, and/or Zn-bound proteins, depending on the particular chelator and conditions used. As a result, assuming the composition of such operationally-defined pools is risky.

Our results confirm the major conclusion of many previous investigations that there is no “free” (*i.e.* aqueous) zinc in cells. Regardless of growth conditions, *E. coli* cytoplasm has the capacity to coordinate *at least* 10 μM of added aqueous Zn^2+^ ions (perhaps ten times that). Our results with TPEN and EDTA indicate that the *labile pools* associated with these chelators are primarily due to loss of Zn from proteins rather than from low-mass complexes, opposite of what is often assumed.

### Composition of the low-mass Zn pool in *E. coli*

The low-mass Zn pool in *E. coli* cytoplasm consists of about a half-dozen Zn coordination complexes. Since most of these complexes remain unidentified, we have named them according to their elution volumes: Zn15.7, Zn16.2, Zn16.8, Zn17.1, and Zn18.7. Some peaks partially overlap each other, and there is some variability in volumes (estimated at ± 0.1 ml). Of these, Zn16.8 showed the most dynamic behavior, largely absent in the cytoplasm from unsupplemented cells, and dominating in the cytoplasm of cells grown on media supplemented with 100 μM Zn(acetate)_2_. Under those latter conditions, we were able to isolate that peak and identify by ESI-MS that it contains two Zn-GSH complexes, namely Zn coordinated to one and to 2 GSH molecules. We were unable to identify other members of the low-mass Zn pool by ESI-MS because concentrations were too low and/or salt concentrations were too high. However, Zn forms a stable complex with cysteine, which migrates near the Zn18.7 peak. As such, we tentatively assign that peak to a Zn-cysteine complex.

### Size of the low-mass Zn pool in *E. coli*

Our results suggest collective concentrations for the low-mass Zn pool of *E. coli* in the 5 to 200 μM range, 4 to 11 orders of magnitude higher than previous reports for “free,” “loosely bound,” or “labile” Zn. One explanation for this discrepancy is that we have *overestimated* the size of the low-mass Zn pools, despite our efforts to lyse cells gently. Alternatively, previous studies may have *underestimated* the size of these pools, a possibility that we now consider.

Most or all of the fluorescent-based chelators that have been used to detect labile Zn pools have been designed to be *selective* for low-mass pools. However, our results show that TPEN removes Zn from Zn-bound proteins about as effectively as it removes Zn from low-mass complexes. Other designer chelators, including Zinquin and Fluo-Zin-3 also remove some Zn from proteins (20). However, the selectivity required to detect pM or fM pool concentrations in a background of 100 μM of Zn-bound to proteins would be extraordinary. Even if a chelator were 99.99% selective for low-mass Zn complexes, the 0.01% of Zn removed from 100 μM of Zn proteins would correspond to 10^−8^ M Zn, orders-of-magnitude higher than needed to detect a pM or fM pool. Given the dominance of Zn-bound proteins in the cell, it is reasonable to consider that some of the Zn detected by chelators in previous in-cell studies was removed from proteins. Interestingly, correcting for this possibility would *lower* the size estimate of the low-mass pools, making the discrepancy with previous chelator studies more severe.

Femtomolar or pM concentrations for “free” Zn in cells have been justified by arguing that pool size is controlled by the position of the metal within the Irving-Williams series (3, 27, 28), Mn^2+^ < Fe^2+^ < Co^2+^ < Ni^2+^ < Cu^2+^ > Zn^2+^ and we agree with that for aqueous metal ions. In this series, divalent first-row d-block transition metals are ranked according to the stability of the complexes formed with classical ligand donors (L = O, N, S), *relative to aqueous metal ions*. Consider the simple dissociation reaction in which a Zn complex dissociates into aqueous Zn ions and ligand L, Equation 1.

(1) $$ZnL\overset{K_{DL}}{⇄}Zn+L;\;K_{DL}=\frac{\left[Zn\right]\left[L\right]}{\left[ZnL\right]}\text{;}$$

$$K_{DL}=\left[Zn\right]\;\text{when}\;\left[ZnL\right]=\left[L\right]$$

When the concentration of the complex and L are equal ([ZnL] = [L]), the aqueous Zn concentration equals the dissociation constant *K*_*DL*_. The more stable the complex, the lower the concentration for aqueous Zn. Zn complexes are among the most stable in the series, and so the concentration of aqueous Zn in the cell should be among the lowest. For example, *K*_*D*_ for the Zn-GSH complex is 1.8 × 10^−15^ M (24). If [GSH] = 1 mM and [Zn-GSH] = 100 μM, the concentration of aqueous Zn in such solutions would be 10^-16^ M, consistent with previous estimates.

Since aqueous Zn binds so strongly to many apo-proteins, binding titrations cannot be performed using aqueous Zn directly. Rather a stable Zn coordination complex (ZnL) whose *K*_*DL*_ is well-known (obtained from titrations using aqueous Zn as the titrant) is used. The actual titration reaction can then be represented by Equation 2.

(2) $$ZnL+apoP\overset{K_{eq}}{⇄}L+ZnP;\;K_{eq}=\frac{\left[ZnP\right]\left[L\right]}{\left[ZnL\right]\left[apoP\right]}$$

Since the apo-protein (apo-P) presumably binds Zn tighter than does L, *K*_*eq*_ > 1. Then (Equation 3)

(3) $$ZnP\overset{K_{DP}}{⇄}Zn+apoP;\;K_{DP}=\frac{\left[ZnP\right]}{\left[Zn\right]\left[apoP\right]}\text{;}$$

$$K_{DP}=K_{DL}/K_{eq}$$

can be applied to obtain the desired constant for binding aqueous Zn to the apo-protein (KDP). For example, if *K*_*DL*_ = 10^−15^ and *K*_*eq*_ = 10^3^, then *K*_*DP*_ would equal 10^−18^ M, comparable to values obtained for Zur and ZntR.

However, since cells are devoid of aqueous Zn, Zn from the low-mass pool is presumably being transferred directly to apo-proteins. Thus, the relevant reaction in estimating the size of the pool should be Equation 2 where ZnL represents the *low-mass* pool. In that case, the expected concentration of the pool, when [ZnP] = [apoP], would equal [L]/*K*_*eq*_. If [L] = 10^−3^ M, as expected for L = GSH, and if *K*_*eq*_ = 10^3^, the low-mass Zn pool concentration would be 10^-6^ M, similar to what we observed. Thus, the *concentration of low-mass Zn. pool complexes within cells need not adhere to the Irving-Williams series rule*.

### Zn trafficking, metallation, and homeostatic in *E. coli*

Aqueous Zn is commonly assumed to play a central role in Zn homeostasis. For example, “free” Zn within the cell was presumed to bind transcription factors Zur and ZntR. Consistent with that, *all* in-vitro Zn binding titrations of these and other proteins have used aqueous Zn as the titrant, yielding pM - fM *K*_*D*_ values. Marszalek *et al.* (29) recognized the problem of calibrating fluorescent Zn probes against aqueous Zn ions – namely that doing so will yield what they called “false free zinc” concentrations. We have no doubt that all such numbers were calculated accurately. But if cells are devoid of aqueous Zn, such Zn ions cannot be involved in Zn homeostasis. Instead, we propose that the low-mass Zn pool (or conceivably the broader *labile* pool) serves this function. In that case, *K*_*D*_’s that have been determined by titrating apo-proteins with aqueous Zn (rather than with low-mass Zn complexes) would be orders of magnitude lower (tighter binding) than are relevant for understanding homeostasis. In brief, the relevant equilibrium reaction involved in Zn homeostasis would look more like Equation 2 than Equation 1 or Equation 3.

**Figure 10:**
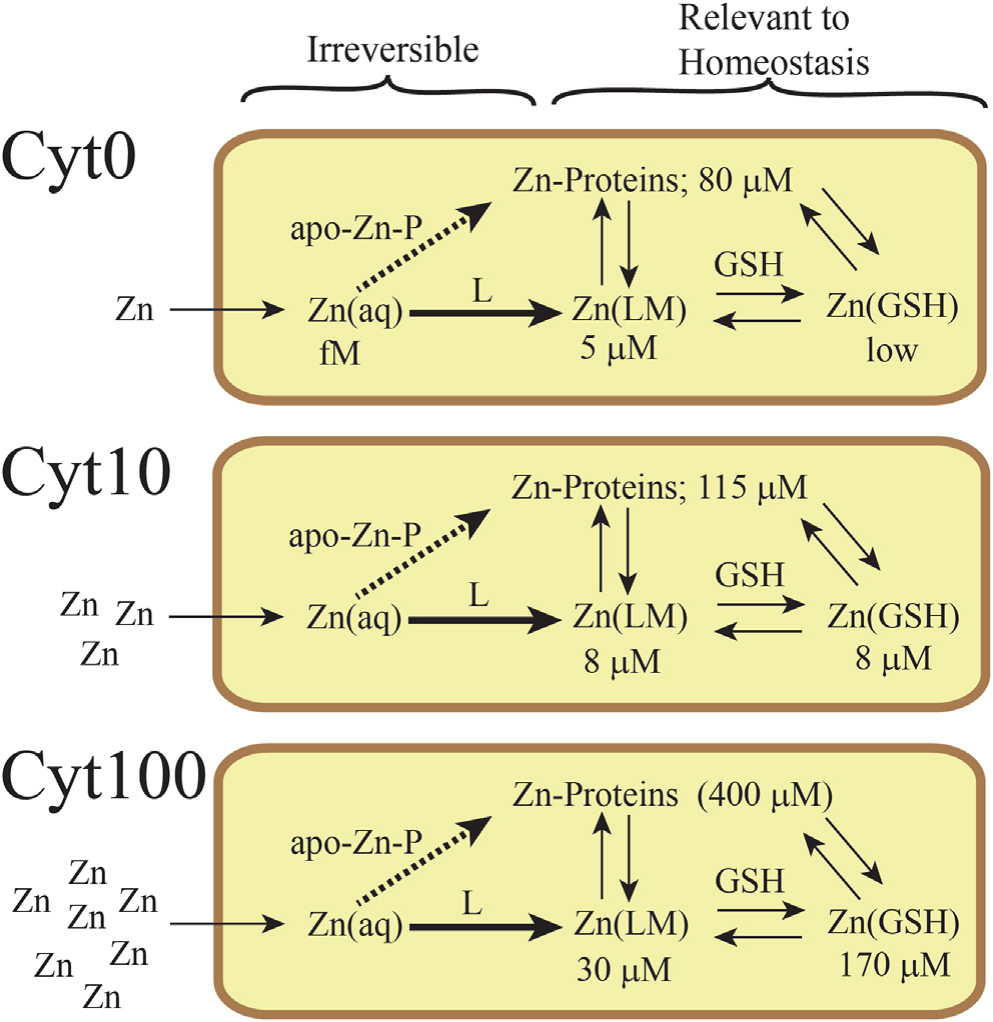
**Zn trafficking model.** Zn enters cells as aqueous Zn^2+^ ions that are rapidly, quantitatively, and irreversibly bound to various ligands L to generate the low-mass Zn pool and no (fM) aqueous Zn. A portion of aqueous Zn may directly metallate apo-proteins. Under Zn-limited conditions (0 μM Zn supplementation), the low-mass Zn pool represents a tiny fraction of the total Zn in the cytoplasm, and little if any Zn(GSH)_1_ or Zn(GSH)_2_ is present. Under Zn-sufficient conditions (Cyt10), the concentrations of both the Zn-bound proteins and the labile zinc pool are greater. Moderate levels of Zn-GSH are evident due to the increase in low-mass Zn levels, not [GSH]. Much of the low-mass Zn in Zn-replete cells (Cyt100) coordinates to GSH, perhaps to minimize toxic effects of excess low-mass Zn.

The insights offered by our results regarding Zn trafficking, metallation, and homeostatic regulation in *E. coli* are summarized by the working model in Figure 10 where intracellular Zn is divided into aqueous Zn, low-mass Zn, and Zn-bound proteins. Nutrient Zn enters the cell as aqueous Zn, which is rapidly, quantitatively, and irreversibly coordinated to various non-proteinaceous ligands that, once coordinated, constitute the low-mass pool. We were impressed by the quantity of apo-proteins that become metallated when aqueous Zn ions are added to the cytoplasm. Although there may be undiscovered Zn-binding chaperones that mediate that process, some apo-proteins may bind aqueous Zn directly. Apo-proteins are also likely metallated by low-mass pools. Regarding homeostasis, our results indicate that Zn concentrations in isolated cytoplasm and FTSs increase significantly as the concentration of Zn in the growth media increases. We do not regard this as “tight” regulation in that these cells do not strictly limit the entry of excess Zn under high media metal concentrations. Rather, much of the excess Zn that enters cells binds GSH, thereby mitigating the toxic effects of excess intracellular Zn.

## Experimental procedures

### Cell growths

Forty-two batches of MG1655-pZa31mycR cells were grown aerobically in 1 L of M9 media containing 0.4% (w/v) glucose, 1 mM chloramphenicol (Sigma-Aldrich), and 0, 10, or 100 μM Zn(acetate)_2_. 50 mL precultures were inoculated with cells and allowed to grow for 24 h at 37 °C with 250 rpm shaking. Cultures were then transferred to 1 L of M9 media and allowed to grow to a final OD_600_ of 0.8 to 1.2. Cells were spun at 4000× *g* for 15 min and then resuspended with HPW in centrifuge tubes that were acid washed to remove Zn contamination prior. Resuspended cells were spun at 4000× *g* for 10 min. This process was repeated. The wash solution was discarded, and the mass of the pellet was recorded. The cell pellet was then placed in a −80 °C freezer until processed.

For three batches of 100 μM Zn(acetate)_2_ supplemented cells, the cells were washed 2× with 1 mM EDTA solution in HPW before washing 2× with HPW. The lysis step and subsequent analysis by LC-ICPMS and ICP-MS were performed repeated as described above.

### Cytoplasm isolations

Cells were thawed in an anaerobic, refrigerated glovebox (MBraun Labmaster 120, 1–10 ppm O_2_, 4 °C) for 1 h. The cell lysate was removed from the box in a sealed centrifuge tube (previously acid washed) and incubated at 37 °C, 250 rpm shaking with 4 μl/g of pellet of 1.12 mg/ml DNase (Sigma-Aldrich) for 1 h. Lysate was spun at 100,000× *g* for 1 h with a Beckman Coulter SW32Ti rotor in an Optima L-90K Ultracentrifuge. The resulting supernatant was then filtered with either a Titan 0.2 μm filter (Thermo Scientific) or an Amicon Ultra 3 kDa cutoff membrane centrifugal filter (Millipore Sigma). Filtered samples were defined as cytoplasm (Cyt) or flow-through solutions (FTSs), respectively.

### Metal analyses

A set of five ICP-MS standards was prepared *via* 10-fold serial dilution of TEXSAM-15REV3 (Inorganic Ventures). The standards had a final concentration of 5% trace-metal-grade (TMG) HNO_3_ (Fisher Scientific) or 5% HNO_3_ with 2.5% H_2_O_2_ (Acros Organics). Two blanks of HPW with 5% HNO_3_ or 5% HNO_3_ with 2.5% H_2_O_2_ were also prepared. For elemental analysis of the samples, three aliquots of the resulting Cyt or FTS, ranging from 50 to 150 μl, were transferred to previously acid washed 15 ml polypropylene tubes (Agilent). To each tube was added 150 μL of TMG HNO_3_. Tubes were capped, sealed with electrical tape, and incubated at 80 °C for 24 h. Between 0.10 and 0.25 g of whole-cell wet-pellet samples were suspended in 2-fold diluted or concentrated TMG HNO_3_ and transferred to 15 ml polypropylene tubes and digested similarly. If any resulting sample remained undissolved, 75 μl of H_2_O_2_ was added, and the sample was resealed and digested for an additional 24 to 72 h. Samples were then aliquoted into three separate tubes and diluted with HPW to obtain final concentrations of 5% HNO_3_ or 5% HNO_3_ with 2.5% H_2_O_2_. Analyses were performed on an Agilent 8900 inductively coupled mass spectrometer (ICP-MS) with reaction cell gases set to 2.0 ml/min H_2_ 30% O_2_. An internal standard IV-ICPMS-71D (Inorganic Ventures) was prepared in 5% HNO_3_. Cells were presumed to be 72% of the pellet mass, with a density of 1.10 g/ml.

### LC-ICP-MS experiments

A standard metal stock solution of 1 mM Zn(acetate)_2_ dihydrate (Acros Organics) was prepared in HPW. 10 millimolar ligand stock solutions of L-aspartate (MP Biomedicals), monosodium glutamate monohydrate (Acros Organics), L-histidine (MP Biomedicals), L-methionine (MP Biomedicals), sodium citrate (Fisher Scientific), reduced glutathione (GSH) (Sigma Aldrich), oxidized glutathione (GSSG) (Sigma Aldrich), and L-cysteine (Sigma Aldrich) were prepared in HPW. Zn-ligand standards were diluted to final concentrations of 2 μM Zn and 1 mM of the given ligand in 20 mM ammonium bicarbonate (ABC) (Sigma Aldrich) pH 7.5. To match the concentrations of ^23^Na, ^24^Mg, and ^39^K in the isolated Cyt samples, metal-ligand standards were spiked with 100 mM stocks of NaCl (Sigma Aldrich), MgCl_2_ hexahydrate (Thermo Scientific), and KCl (EMD) to obtain final concentrations of 5 mM, 2 mM, and 30 mM, respectively. A standard containing a variety of potential zinc-binding ligands, termed a pseudo-flow-through solution (pFTS) was prepared according to Brawley *et al.* (22). One hundred microliters of standards, Cyt, or FTSs were injected onto a Superdex 30 *Increase* column (Cytiva) installed in an Agilent 1260 liquid chromatography (LC) system with a bio-inert quaternary pump (G5611A), multisampler (G5688A), diode array (G4212B), and fraction collector (G5664A), all housed within the glovebox. The LC was connected in tandem to the ICP-MS (external to the box) to collect elemental data for ^32^S, ^64^Zn, ^66^Zn, ^67^Zn, and ^68^Zn. The counts for the zinc isotopes were summed to obtain total zinc counts. The mobile phase was 20 mM ABC pH 7.5 that had been filtered with a 0.22 μm polyethersulfone stericup filter (Corning) and then degassed on a Schlenk line. Flow rate was 0.6 ml/min and data were collected for 1 h per sample. Between sample injections, the column was cleaned by injecting 100 μl of a chelator cocktail consisting of 500 μM each of ethylenediaminetetraacetic acid (EDTA) (Sigma Aldrich), ethylene glycol-bis(β-aminoethyl ether)N,N,N′,N′-tetraacetic acid (EGTA) (Sigma-Aldrich), 1,10-phenanthroline (phen) (Acros Organics), 2,2-bipyridine (BPY) (Alfa Aesar), bathocuproinedisulfonic acid (Sigma Aldrich), deferoxamine (EMD Millipore), TPEN (Sigma-Aldrich), and 10 mM ascorbic acid (Acros Organics). This was followed by injecting 100 μl of HPW to wash the column of the chelator cocktail. This column washing procedure was repeated between sample injections. Analyses were performed with reaction cell gases as described above. Peaks were fitted using OriginPro (originlab.com) using Analysis, Peaks and Baselines, Multiple Peak fit, and Gaussian lineshapes.

### Zn spiking

A stock solution of 100 μM Zn(acetate)_2_ was prepared in HPW. This solution was added to 150 μl of Cyt or FTS samples to obtain a final concentration of 2, 5, or 10 μM added Zn, and this solution was allowed to react in the glovebox for at least 3 h. Counts for total Zn and ^32^S were adjusted to account for dilution. For Cyt0, the isolated cytoplasm was spiked with a stock solution of 100 μM ^67^Zn to obtain final concentrations of 2, 5, or 10 μM ^67^Zn. For FTS0, the isolated FTS was spiked with a stock solution of 1.25 mM ^67^Zn to obtain a final concentration of 100 μM ^67^Zn or with a stock concentration of 10 mM GSH to obtain a final concentration of 1 mM GSH. Counts for ^67^Zn and ^32^S were adjusted to those expected for cytoplasmic concentrations by accounting for dilution.

### TPEN titration

A stock solution of 500 μM TPEN was prepared in HPW. This solution was added to 150 μL of Cyt samples to obtain final concentrations of 5, 10, 20, or 50 μM TPEN. Each solution was incubated in the glovebox for ≥ 3 h. Counts for total zinc and ^32^S were back-calculated to account for dilution.

### ESI-MS experiment

Five hundred microliters of Cyt obtained from 100 μM Zn supplemented cells were injected into the LC-ICPMS for fraction collection. Three 500 μl fractions from those with elution volumes of 16 to 17.5 ml were collected. A 25 μl aliquot of each fraction was diluted 1:1 in LC-MS grade methanol (Fisher Scientific) for direct injection by electrospray ionization mass spectrometry (ESI-MS). The remainder was transferred anaerobically to a Labconco FreeZone 2.5 Plus lyophilizer and allowed to lyophilize for ∼12 h to dryness. The samples were transferred anaerobically to the glovebox where they were resuspended in 25 μl HPW. The resulting solutions were diluted 1:1 in methanol for direct injection analysis by ESI-MS. A standard of 10 μM aqueous Zn plus 1 mM GSH was prepared in 20 mM ABC pH 7.5 from stocks of 1 mM Zn(acetate)_2_, 10 mM GSH, and 100 mM ABC pH 7.5. The standard was diluted 1:1 in methanol for analysis by ESI-MS. ESI-MS analysis was performed on a Thermo Scientific Q Exactive Focus instrument. The samples were injected into a 10 μl loop flowing with a methanol mobile phase at 600 μl/min. The Q Exactive Focus HESI was operated in positive mode and collected data from 300 to 800 m/z. The conditions of the ESI-MS include: spray voltage, 3.5 kV; sheath gas, 40 AU; auxiliary gas, 10 AU; transfer capillary, 270 °C; S-lens RF level, 50V; and auxiliary gas heater, 250 °C. Data were collected and analyzed using Exactive Series 2.11/Xcalibur 4.2 software. Collected masses were accurate to within ± 2 ppm.

### qPCR experiment for gshA expression

Five mL of minimal media containing either 0, 10, or 100 μM Zn(acetate)_2_ was transferred to three 15 ml Falcon tubes. The media was inoculated with a colony of MG1655 pZa31mycR cells, which were agitated using a spinning wheel at 37 °C. This was repeated in triplicate for all three conditions. RNA was extracted using an Aurum Total RNA Mini Kit. The RNA was converted to cDNA using the Applied Biosystems High-Capacity RNA-to-cDNA Kit on a Bio-Rad T100 Thermal Cycler. 10 nanograms of cDNA was then used for qPCR. qPCR data for *gshA* expression were collected using the CFX96 Real-Time System on a C100 Touch Thermo Cycler. The data were analyzed using CFX Maestro. The average Cq values for n = 3 for each condition are reported along with individual data points and were plotted using Origin-Pro. Error bars represent ± SD.

## Data availability

All data are contained within the manuscript and Supporting Information.

## Supporting information

This article contains supporting information (22).

## Conflict of interest

The authors declare that they have no conflicts of interest with the contents of this article.

## Supporting information

Supporting information

Data and Analysis for Figures

Chromatograms and simulations
