# Supplementary material for: Low-mass zinc pools in Escherichia coli: Micromolar concentrations, diverse compositions, and Zn-glutathione dominating under Zn-replete conditions
Source: J Biol Chem. 2025 Jul 29;301(8):110362. doi: 10.1016/j.jbc.2025.110362 (PMC12329115; doi:10.1016/j.jbc.2025.110362)
Supplement: Supporting information [file mmc1.docx]

Supplemental Information for…

Low-mass zinc pools in *Escherichia coli*: micromolar concentrations, diverse compositions, and Zn-glutathione dominating under Zn replete conditions

Alexia C. Kreinbrink, Nicholas Romano, Justin D. Hierholzer, and Paul A. Lindahl

Table of Contents:

Figure S1: Mössbauer spectra of *E. coli* cells before (A) and after (B) lysis.

Figure S2. LC-ICP-MS Chromatogram of Zn(acetate)_2_.

Table S1. Zn Calibration factors.

Figure S3: Stability of Zn proteins and complexes with time.

Figure S4. Effects of spiking Cyt10 and Cyt100 with TPEN.

Table S2: Estimated Zn concentrations of cytosol and FTS samples, divided into proteins and labile Zn pool fractions.

Figure S5. Weakly binding Zn complexes.

Figure S6. RT-qPCR of gene expression of *gshA* for cells grown on media supplemented with 0, 10, or 100 μM Zn.

Separate Documents:

1. Chromatograms and Simulations

2. Data and analysis for figures

**Figure S1: 5 K, 0.05 T Mössbauer spectra of *E. coli* cells before and after lysis. Left panel:** A, intact cells; B, lyzed cells. Mössbauer (MB) spectra were collected on a MS4 WRC spectrometer (SEE Co, Edina MN) at ~5K and 0.05 T. An α-iron foil was used for room temperature calibration. The magnetic field was applied parallel to the gamma radiation. Cells were grown in 10 μM ^57^Fe(III)citrate in M9 media. At an OD_600_ of 0.8-1.2, they were spun and collected as described above. Cells were pelleted by centrifugation into a MB cup and then frozen in liquid N_2_. An initial MB spectrum was collected, and then the cells were thawed in the glovebox for 1 hr to ensure lysis. The lysate was refrozen in liquid N_2_ in the glovebox and a MB spectrum was collected. The right panel is of whole cells before (A) and after (B) being lyzed aerobically and at room temperature.


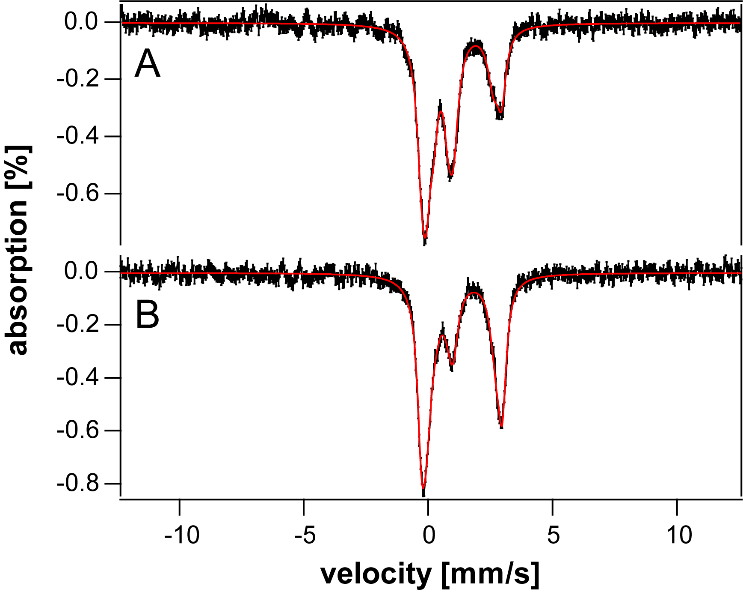

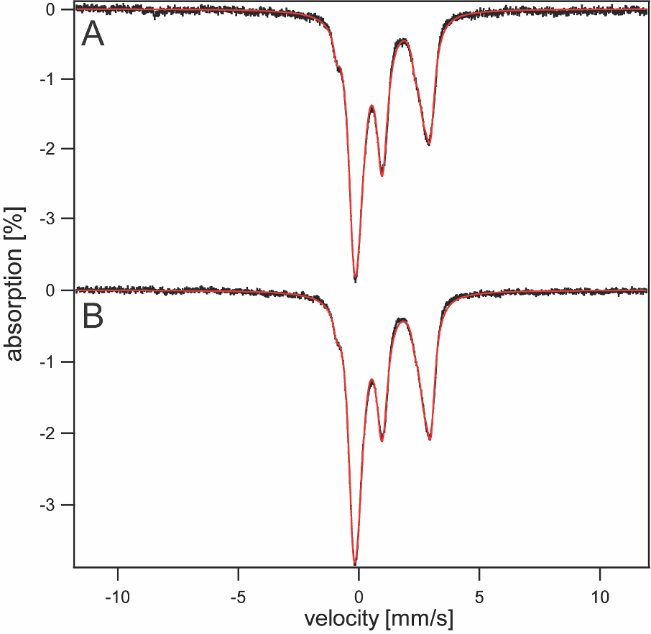


**Analysis:** Spectra were dominated by 2 quadrupole doublets, including one that arose primarily from S = 0 [Fe_4_S_4_]^2+^ clusters (δ = 0.42 mm/s; ∆E_Q_ = 1.09 mm/s; Γ = 0.56 mm/s) and the other from nonheme S = 2 Fe^II^ species (δ = 1.36 mm/s; ∆E_Q_ = 2.92 mm/s; Γ = 0.51 mm/s). Due to the gentle lysis, 3% - 5% of cellular iron was converted from iron-sulfur clusters into Fe^II^ (left panel) which was significantly less than obtained previously (20% of cellular iron converted into NHHS Fe^II^), shown on the right panel (adapted from (22)).

**Figure S2. LC-ICP-MS Chromatogram of Zn(acetate)_2_.** (A), 2 µM Zn(acetate)_2_ injected on the “ghost column” to obtain total zinc counts in the sample. (B), Same as (A) but injected on Superdex 30 Increase. Multiplication factors: Total Zn x 1.


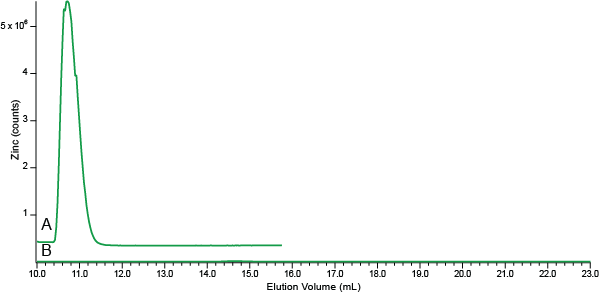


**Analysis:** The integrated Zn intensity in A serves as a control for trace B in which the same sample was injected onto the column. If none of the Zn adsorbed on the column, trace B would have shown a peak of the same integrated intensity. The absence of any peak indicates that aqueous Zn was quantitatively adsorbed by the column.

**Table S1. Zn Calibration factors:** Ten experiments provided an opportunity to calculate a Zn calibration factor, in units of counts detected per µM of Zn injected (using a 100 µL injection loop). Variations arise from daily differences in tuning the instrument and column condition. The proportion of Zn that adsorbed onto the column ranged from < 10% to more than 90%, depending on the stability of the Zn complex, with aqueous Zn ions adsorbing quantitatively and very stable Zn complexes such as Zn bound to chelates, adsorbing least. Labile Zn pool complexes are of intermediate stability. Relative to the cytosolic and FTS Zn concentrations listed in Table 1, the Zn concentration of samples injected into the LC-ICP-MS system had been diluted by a factor of 1.35. The calibration factors from 2 experiments were excluded in calculating the average value and standard deviation. Calibration factors obtained from standards were also excluded, due to the absence of salt effects which suppresses signals.

| Experiment | Calibration factor  (counts per uM Zn) |
| --- | --- |
| Cyt0 Total Zn intensities divided by average Zn Cyt0 concentration in Table 1 | 624856 |
| Cyt10 Total Zn intensities divided by average Zn Cyt10 concentration in Table 1 | 628324 |
| Cyt100 Total Zn intensities divided by average Zn Cyt100 concentration in Table 1 | 274044 |
| Slope of increases in Cyt0 total Zn intensities per uM ^67^Zn added. | 366832 |
| FTS0 total Zn intensities divided by average Zn FTS0 concentration in Table 1 | 2920373 |
| FTS10 total Zn intensities divided by average Zn FTS10 concentration in Table 1 | 783510 |
| FTS100 total Zn intensities divided by average Zn FTS100 concentration in Table 1 | 503632 |
| Slope of FTS0 total Zn intensity per uM Zn added | 493947 |
| Slope of FTS10 total Zn intensity per uM Zn added | 552172 |
| Slope of FTS100 total Zn intensity per uM Zn added | 763886 |
|  |  |
| Average (excluding Cyt100 and FTS0) | 589645 |
| Standard Deviation | 131476 |
| Coefficient of variation | 0.22 |
|  |  |
| Zn intensity divided by µM Zn in the Zn-cysteine standard | 3803000 |
| Zn intensity divided by µM Zn in the Zn-GSH standard | 2607000 |
| Zn intensity divided by µM Zn in the Zn-citrate standard | 20000 |
| Zn intensity divided by µM Zn in aqueous Zn (Zn(acetate)_2_ | 0 |

**Figure S3: Stability of Zn proteins and complexes with time.** Panel A: (*i*), Cytoplasm freshly isolated from cells grown in media without supplementation; (*ii* - *iii*), same as (*i*) but incubated in a refrigerated N_2_-atmosphere glovebox for: (*ii*), 1 day; (*iii*), 2 days; and (*iv*), 3 days. Panel B: Same as (Panel A) but highlighting the low-mass species. The gray trace is the difference chromatogram *i* - *iv*.


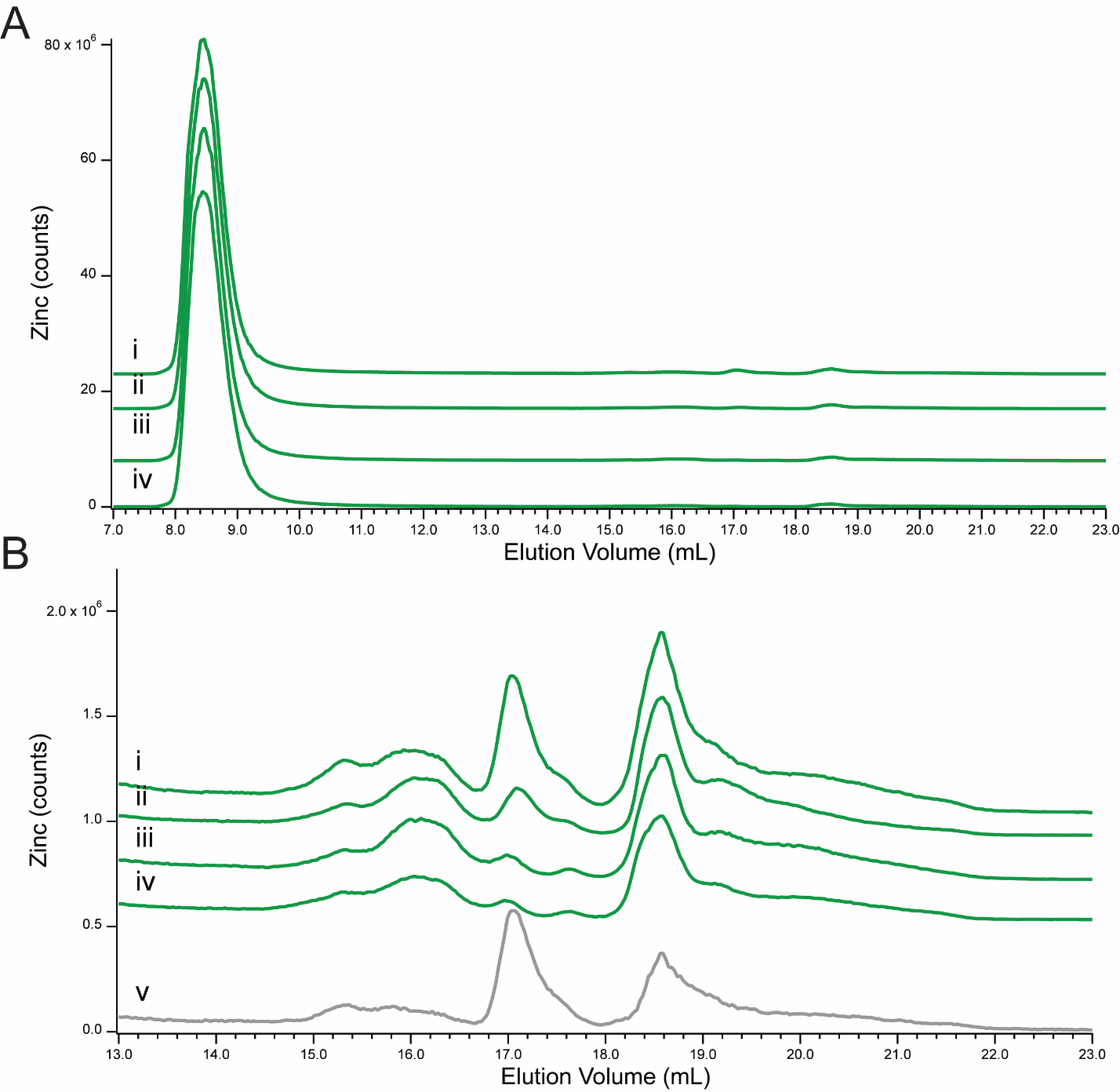


**Figure S4. Effects of spiking Cyt10 and Cyt100 with TPEN.** Panel A: (i) Cytosol from 10 µM Zn supplemented cells; (ii-iv) same as (i) but with the following (final µM) concentrations of TPEN added and adjusted for dilution: (ii) 5; (iii) 10; (iv) 20. Panel B: Same as Panel A but with cytosol from 100 µM Zn supplemented cells.


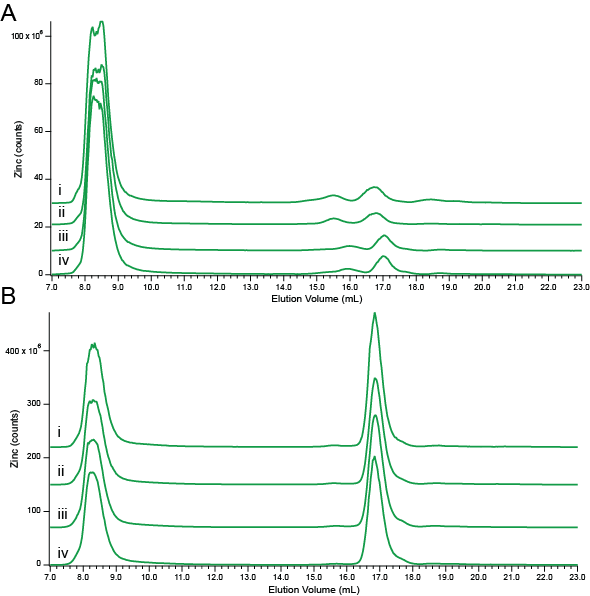


**Table S2: Estimated Zn concentrations of cytosol and FTS samples, divided into proteins and low-mass Zn pool fractions.** Proteins were obtained by summing all Zn peaks with elution volumes less than 10 mL (Zn8.2, Zn8.5, and Zn9.2). Low-mass Zn pool concentrations were obtained by summing all Zn peaks with elution volumes of 11 mL or greater. Zn(GSH) component of the Low-mass Zn pool was represented by the Zn16.7 – Zn16.9 peak. In all cases, intensities were divided by 589,645 counts/µM to obtained estimated concentrations, which have been rounded.

| Sample↓  Zinc concentration (µM)→ | [Zn]total (µM) | [Zn-Proteins] (µM) | [Low-mass Zn Pool] (µM) |
| --- | --- | --- | --- |

|  |  |  | Zn(GSH) | Other |
| --- | --- | --- | --- | --- |
| Cyt0 | 78 | 76 | 0 | 3 |
| Cyt10 | 130 | 120 | 8 | 3 |
| Cyt100 | 410 | 180 | 180 | 40 |
| FTS0 | 2 | 0 | 0 | 2 |
| FTS10 | 10 | 0.1 | 7 | 3 |
| FTS100 | 190 | 0.1 | 180 | 13 |
| Cyt0 (protein stability experiment) | 70 | 66 | 0 | 4 |
| Cyt0.0 ^67^Zn spiking before adding ^67^Zn | 3.5 ^67^Zn | 3.2 ^67^Zn | 0 | 0.14 ^67^Zn |
| Cyt0.10 ^67^Zn spiking after adding 10 µM ^67^Zn | 9.4 ^67^Zn | 8.8 ^67^Zn | 0 | 0.25 ^67^Zn |
| Cyt10.0 used in Zn spiking before adding Zn | 120 | 100 | 8 | 9 |
| Cyt10.10 used in Zn spiking after adding 10 µM Zn | 110 | 90 | 7 | 6 |
| Cyt100.0 used in Zn spiking before adding Zn | 260 | 135 | 110 | 14 |
| Cyt100.10 used in Zn spiking after adding 10 µM Zn | 480 | 250 | 190 | 32 |
| FTS0.0 Zn spiking before adding Zn | 8.3 | 0.1 | 0.6 | 7.7 |
| FTS0.10 Zn spiking after adding 10 µM Zn | 16.4 | 0.2 | 0.6 | 16 |
| FTS10.0 Zn spiking before adding Zn | 12 | 0.1 | 8.4 | 4 |
| FTS10.10 Zn spiking after adding 10 µM Zn | 21 | 0 | 14 | 7 |
| FTS100.0 Zn spiking before adding Zn | 150 | 0.1 | 150 | 4 |
| FTS100.10 Zn spiking after adding 10 µM Zn | 170 | 0.1 | 165 | 4 |
| Cyt0.0 before TPEN spiking | 100 | 92 | 1 | 3 |
| Cyt0.50 after 50 µM TPEN added | 94 | 64 | 0 | 30 |
| Cyt10.0 before TPEN spiking | 130 | 110 | 9 | 12 |
| Cyt10.50 before TPEN spiking | 110 | 66 | 2 | 40 |
| Cyt100.0 before TPEN spiking | 540 | 280 | 230 | 32 |
| Cyt100.50 before TPEN spiking | 460 | 210 | 190 | 60 |

**Figure S5. Weakly binding Zn complexes.** Standards were prepared by mixing 2 µM Zn(acetate)_2_ with 1 mM of the following ligands: (A) ATP; (B) Histidine; (C) Aspartate; (D) Glutamate; and (E) GSSG. The black line in (A) is due to ^31^P. The yellow trace in (E) is due to ^32^S. The detector response for Zn is ca. 1000 times lower than for more stable Zn complexes. Multiplications factors: (A-E) Total Zn x 1, (A) ^31^P x 0.0002; (E) ^32^S x 1. Peaks are the same because they arise from the buffer not the standard.


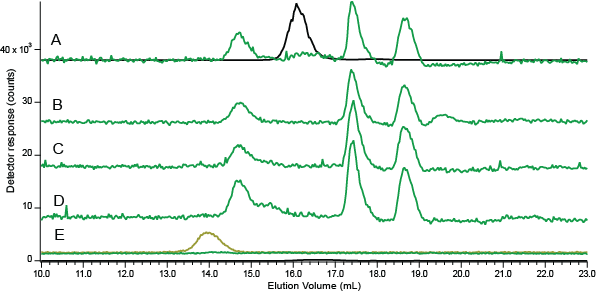


**Figure S6. RT-qPCR of gene expression of *gshA* for cells grown on media supplemented with 0, 10, or 100 μM Zn.** The average Cq values for n=3 for each condition are reported along with the individual data points and was plotted using OriginPro. The error bars represent ± SD

**
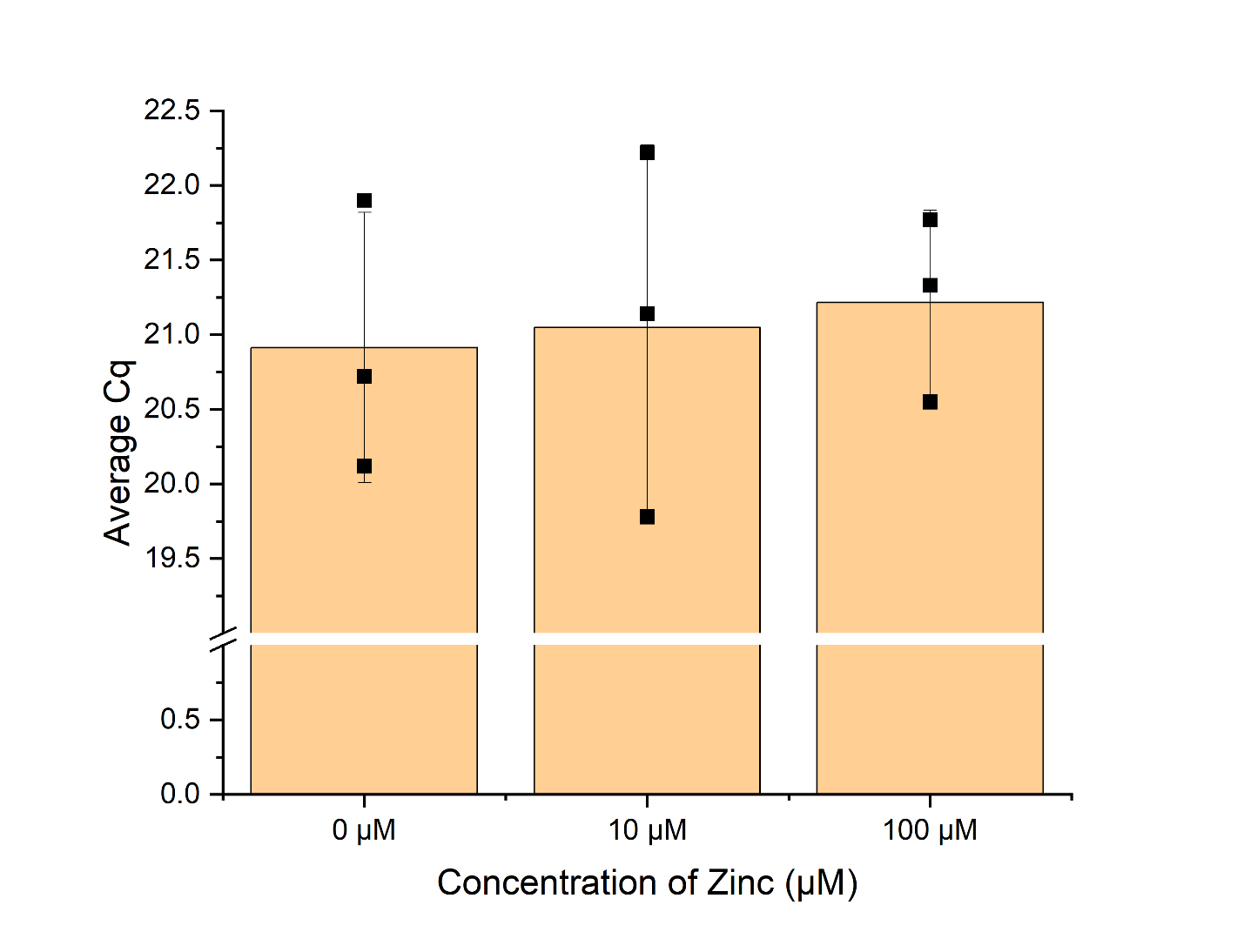
Analysis:** The expression of gshA was unaffected by zinc supplementation; differences were not statistically significant. Rather than this gene being upregulated as originally hypothesized, it appears that more Zn-GSH complex forms with increasing low-mass Zn concentrations in the cytoplasm, perhaps shifting the equilibrium position towards ZnGSH complex formation.
