# Supplementary material for: Low-mass zinc pools in Escherichia coli: Micromolar concentrations, diverse compositions, and Zn-glutathione dominating under Zn-replete conditions
Source: J Biol Chem. 2025 Jul 29;301(8):110362. doi: 10.1016/j.jbc.2025.110362 (PMC12329115; doi:10.1016/j.jbc.2025.110362)
Supplement: Chromatograms and simulations [file mmc3.pdf]

Origin Fitting Graphs; Table of Contents

Pages 1 and 2	Table of Contents
Page 3	Figure 1Ai Cyt0 zinc
Page 4	Figure 1Aii Cyt10 zinc
Page 5	Figure 1Aiii Cyt100 zinc
Page 6	Figure 1Bi Cyt0 sulfur
Page 7	Figure 1Bii Cyt10 sulfur
Page 8	Figure 1Biii Cyt100 sulfur
Page 9	Figure 2Ai Cyt0 zinc
Page 10	Figure 2Aii Cyt0 zinc
Page 11	Figure 2Aiii Cyt0 zinc
Page 12	Figure 2Aiv Cyt0 zinc
Page 13	Figure 2Ai Cyt0 sulfur
Page 14	Figure 2Aii Cyt0 sulfur
Page 15	Figure 2Aiii Cyt0 sulfur
Page 16	Figure 2Aiv Cyt0 sulfur
Page 17	Figure 3A Cyt0.0 zinc
Page 18	Figure 3B Cyt0.5 zinc
Page 19	Figure 3C Cyt0.10 zinc
Page 20	Figure 3D Cyt0.20 zinc
Page 21	Figure 3E Cyt0.50 zinc
Page 22	Associated with Figure 3A; Cyt0.0 sulfur
Page 23	Associated with Figure 3B; Cyt0.5 sulfur
Page 24	Associated with Figure 3C; Cyt0.10 sulfur
Page 25	Associated with Figure 3D; Cyt0.20 sulfur
Page 26	Associated with Figure 3E; Cyt0.50 sulfur
Page 27	Figure 4ABi Cyt0.0 67Zn spiking
Page 28	Figure 4ABii Cyt0.2 67Zn spiking
Page 29	Figure 4ABiii Cyt0.5 67Zn spiking
Page 30	Figure 4ABiv Cyt0.10 67Zn spiking
Page 31	Associated with Figure 4ABi; Cyt0.0 sulfur
Page 32	Associated with Figure 4ABii; Cyt0.2 sulfur
Page 33	Associated with Figure 4ABiii; Cyt0.5 sulfur
Page 34	Associated with Figure 4ABiv; Cyt0.10 sulfur
Page 35	Figure 5ABi FTS0 Zn
Page 36	Figure 5ABii FTS10 Zn
Page 37	Figure 5ABiii FTS100 Zn
Page 38	Figure 5Ci FTS0 Sulfur
Page 39	Figure 5Cii FTS10 sulfur
Page 40	Figure 5Ciii FTS100 sulfur
Page 41	Figure 5Aiv pFTS zinc
Page 42	Figure 5Av ZnGSH zinc
Page 43	Figure 5Avi Zncysteine zinc
Page 44	Figure 5Avii Zncitrate Zn
Page 45	Figure 5Civ pFTS sulfur
Page 46	Figure 5Cv ZnGSH sulfur
Page 47	Figure 5Cvi ZnCysteine sulfur
Page 48	Figure 6Ai FTS0.0 Zn spiking
Page 49	Figure 6Aii FTS0.2 Zn spiking
Page 50	Figure 6Aiii FTS0.5 Zn spiking
Page 51	Figure 6Aiv FTS0.10 Zn spiking
Page 52	Figure 6Bi FTS10.0 Zn spiking
Page 53	Figure 6Bii FTS10.2 Zn spiking
Page 54	Figure 6Biii FTS10.5 Zn spiking
Page 55	Figure 6Biv FTS10.10 Zn spiking

Page 56	Figure 6Ci	FTS100.0 Zn spiking
Page 57	Figure 6Cii	FTS100.2 Zn spiking
Page 58	Figure 6Ciii	FTS100.5 Zn spiking
Page 59	Figure 6Civ	FTS100.10 Zn spiking
Page 60	Associated with Figure 6Ai FTS0.0 Zn spiking sulfur	
Page 61	Associated with Figure 6Aii FTS0.2 Zn spiking sulfur	
Page 62	Associated with Figure 6Aiii FTS0.5 Zn spiking sulfur	
Page 63	Associated with Figure 6Aiv FTS0.10 Zn spiking sulfur	
Page 64	Associated with Figure 6Bi FTS10.0 Zn spiking sulfur	
Page 65	Associated with Figure 6Bii FTS10.2 Zn spiking sulfur	
Page 66	Associated with Figure 6Biii FTS10.5 Zn spiking sulfur	
Page 67	Associated with Figure 6Biv FTS10.10 Zn spiking sulfur	
Page 68	Associated with Figure 6Ci FTS100.0 Zn spiking sulfur	
Page 69	Associated with Figure 6Cii FTS100.2 Zn spiking sulfur	
Page 70	Associated with Figure 6Ciii FTS100.5 Zn spiking sulfur	
Page 71	Associated with Figure 6Civ FTS100.10 Zn spiking sulfur	
Page 72	Figure S3A Cyt10.0 TPEN spiking Zn	
Page 73	Figure S3A Cyt10.5 TPEN spiking Zn	
Page 74	Figure S3A Cyt10.10 TPEN spiking Zn	
Page 75	Figure S3A Cyt10.20 TPEN spiking Zn	
Page 76	Figure S3A Cyt10.50 TPEN spiking Zn	
Page 77	Figure S3B Cyt100.0 TPEN spiking Zn	
Page 78	Figure S3B Cyt100.5 TPEN spiking Zn	
Page 79	Figure S3B Cyt100.10 TPEN spiking Zn	
Page 80	Figure S3B Cyt100.20 TPEN spiking Zn	
Page 81	Figure S3B Cyt100.50 TPEN spiking Zn	
Page 82	Associated with Figure S3A Cyt10.0 TPEN spiking S	
Page 83	Associated with Figure S3A Cyt10.5 TPEN spiking S	
Page 84	Associated with Figure S3A Cyt10.10 TPEN spiking S	
Page 85	Associated with Figure S3A Cyt10.20 TPEN spiking S	
Page 86	Associated with Figure S3A Cyt10.50 TPEN spiking S	
Page 87	Associated with Figure S3B Cyt100.0 TPEN spiking S	
Page 88	Associated with Figure S3B Cyt100.5 TPEN spiking S	
Page 89	Associated with Figure S3B Cyt100.10 TPEN spiking S	
Page 90	Associated with Figure S3B Cyt100.20 TPEN spiking S	
Page 91	Associated with Figure S3B Cyt100.50 TPEN spiking S	
Page 92	Figure S4A Cyt10.0 Zn spiking Zn	
Page 93	Figure S4A Cyt10.2 Zn spiking Zn	
Page 94	Figure S4A Cyt10.5 Zn spiking Zn	
Page 95	Figure S4A Cyt10.10 Zn spiking Zn	
Page 96	Figure S4B Cyt100.0 Zn spiking Zn	
Page 97	Figure S4B Cyt100.2 Zn spiking Zn	
Page 98	Figure S4B Cyt100.5 Zn spiking Zn	
Page 99	Figure S4B Cyt100.10 Zn spiking Zn	
Page 100	Associated with Figure S4A Cyt10.0 Zn spiking S	
Page 101	Associated with Figure S4A Cyt10.2 Zn spiking S	
Page 102	Associated with Figure S4A Cyt10.5 Zn spiking S	
Page 103	Associated with Figure S4A Cyt10.10 Zn spiking S	
Page 104	Associated with Figure S4B Cyt100.0 Zn spiking S	
Page 105	Associated with Figure S4B Cyt100.2 Zn spiking S	
Page 106	Associated with Figure S4B Cyt100.5 Zn spiking S	
Page 107	Associated with Figure S4B Cyt100.10 Zn spiking S	

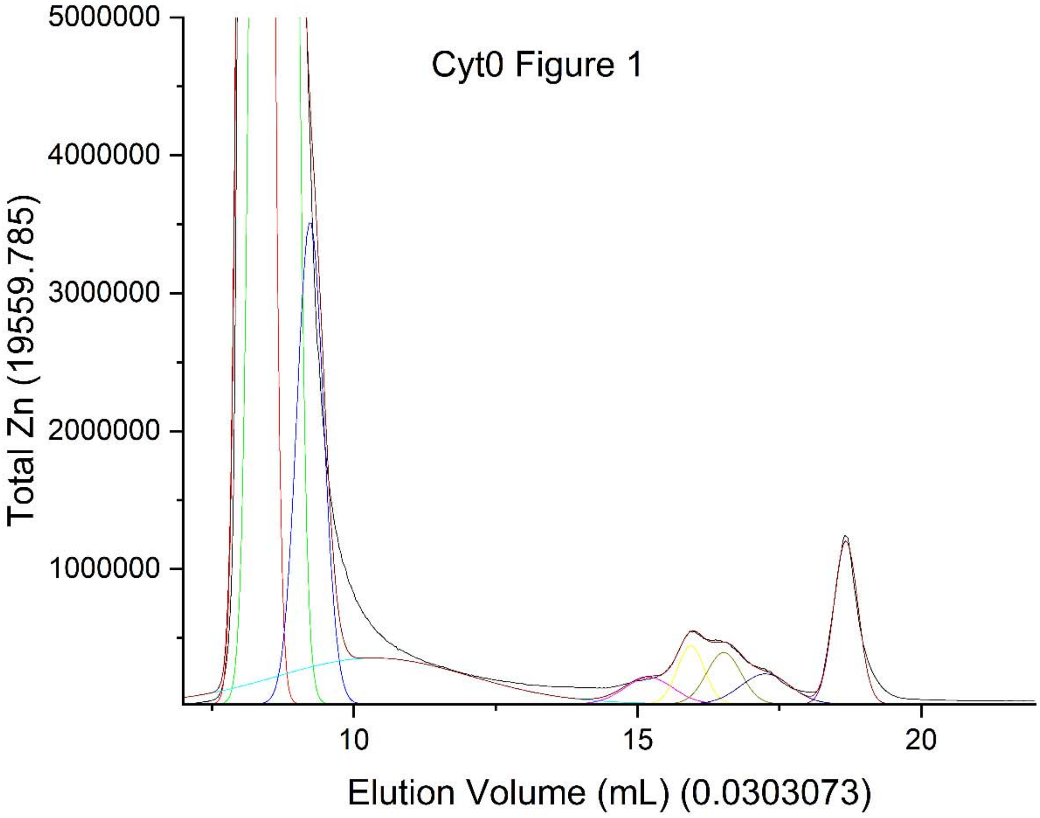
Cyt0 Figure 1
Total Zn (19559.785)
5000000
4000000
3000000
2000000
1000000
10
15
20
Elution Volume (mL) (0.0303073)

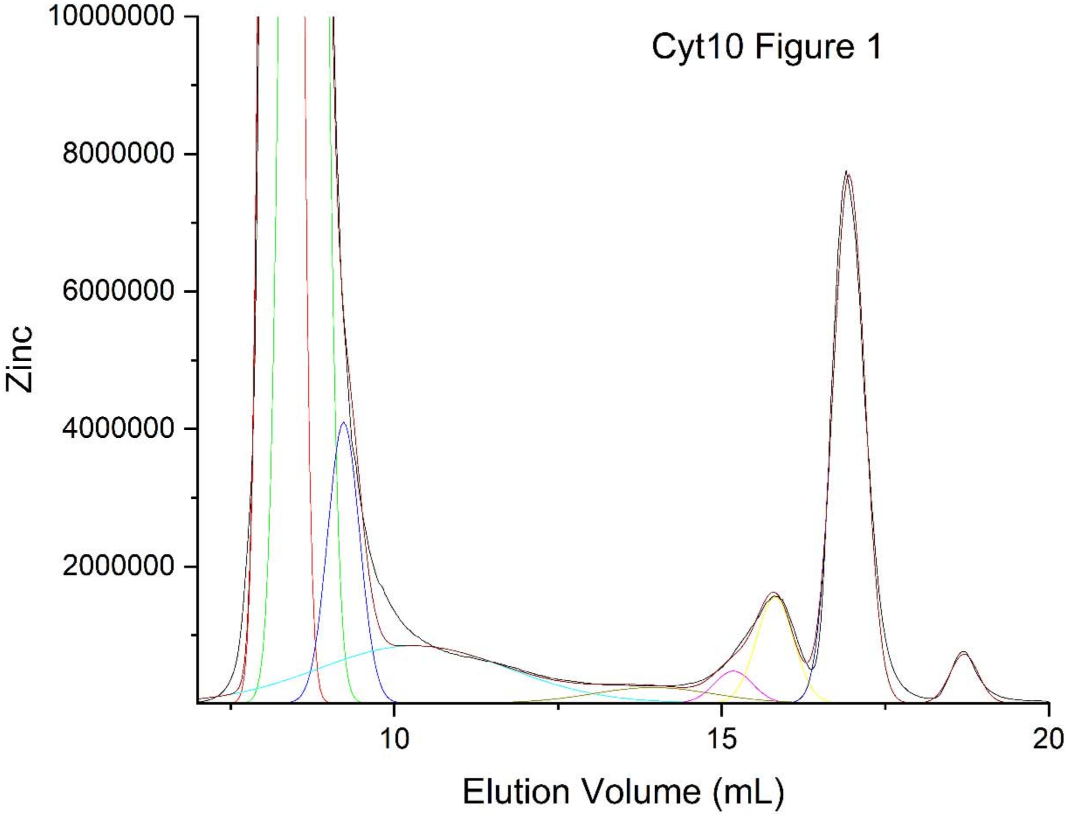
Cyt10 Figure 1
10000000
8000000
6000000
4000000
2000000
Zinc
10
15
20
Elution Volume (mL)

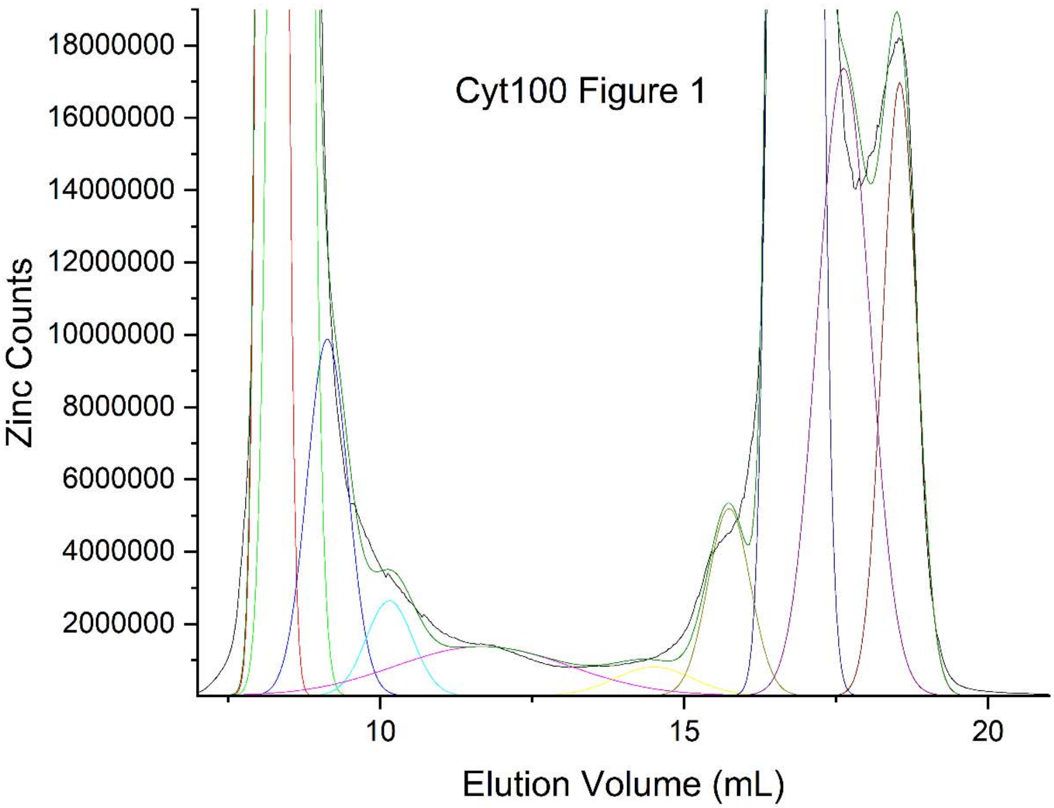
Cyt100 Figure 1
Zinc Counts
18000000
16000000
14000000
12000000
10000000
8000000
6000000
4000000
2000000
10
15
20
Elution Volume (mL)

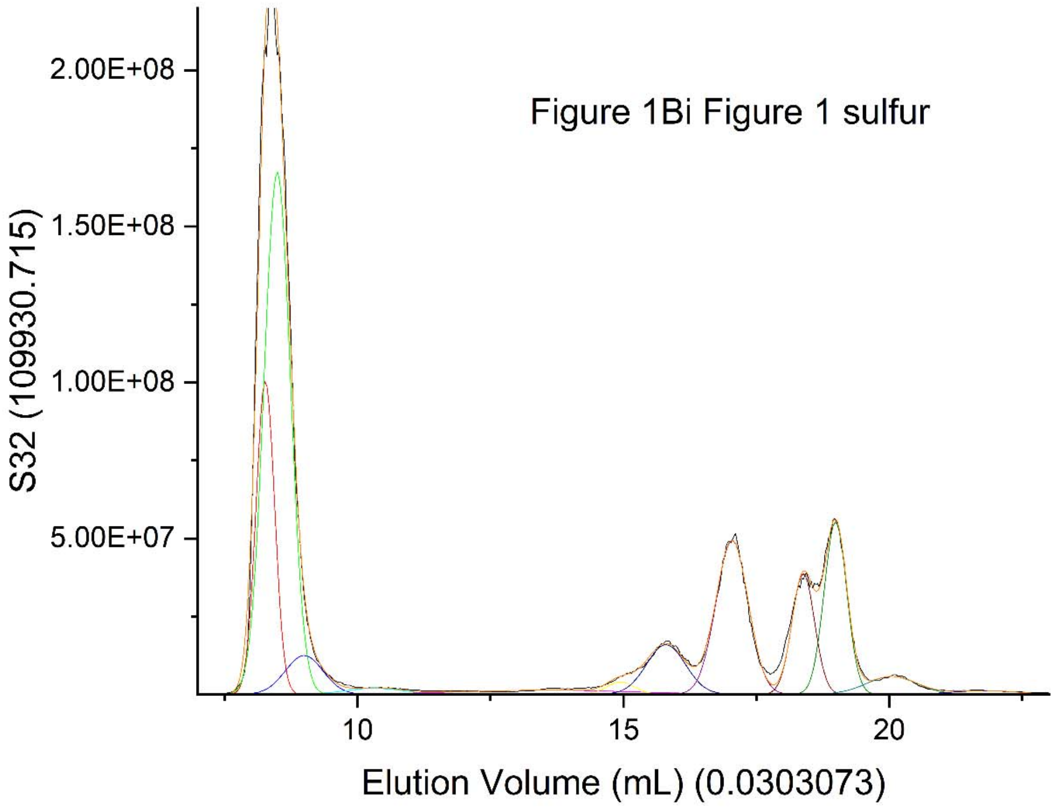

Figure 1Bi Figure 1 sulfur
S32 (109930.715)
2.00E+08
1.50E+08
1.00E+08
5.00E+07
10
15
20
Elution Volume (mL) (0.0303073)

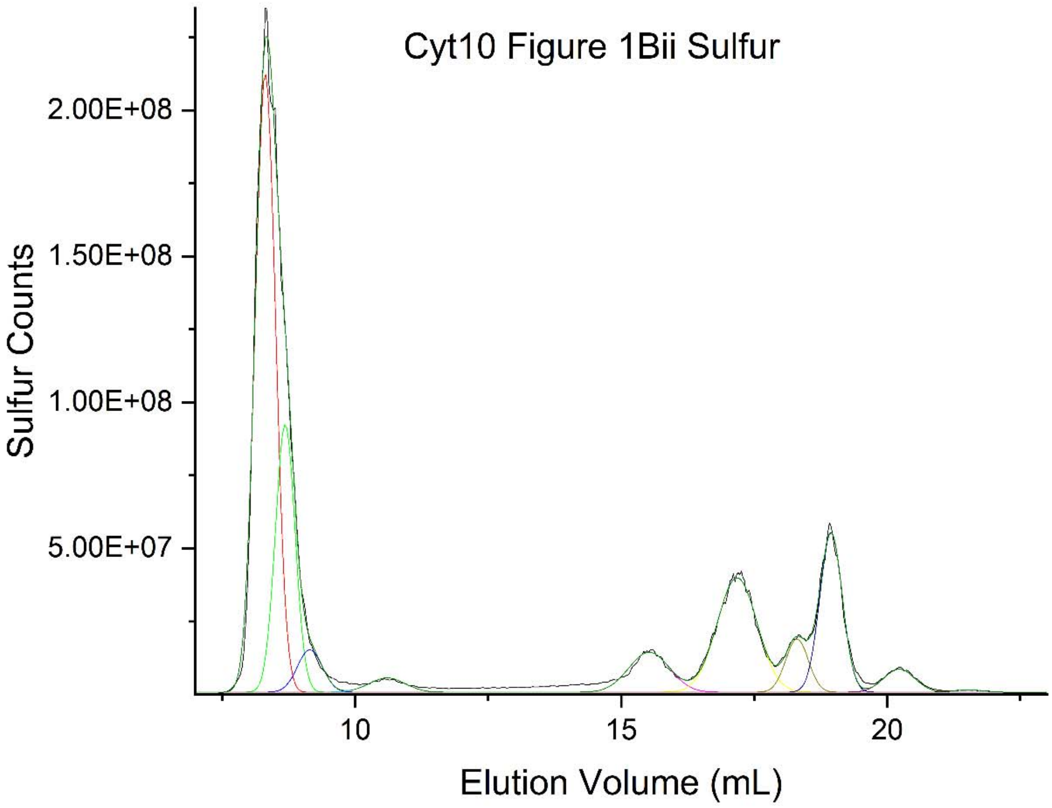

Cyt10 Figure 1Bii Sulfur
2.00E+08
1.50E+08
1.00E+08
5.00E+07
Sulfur Counts
10
15
20
Elution Volume (mL)

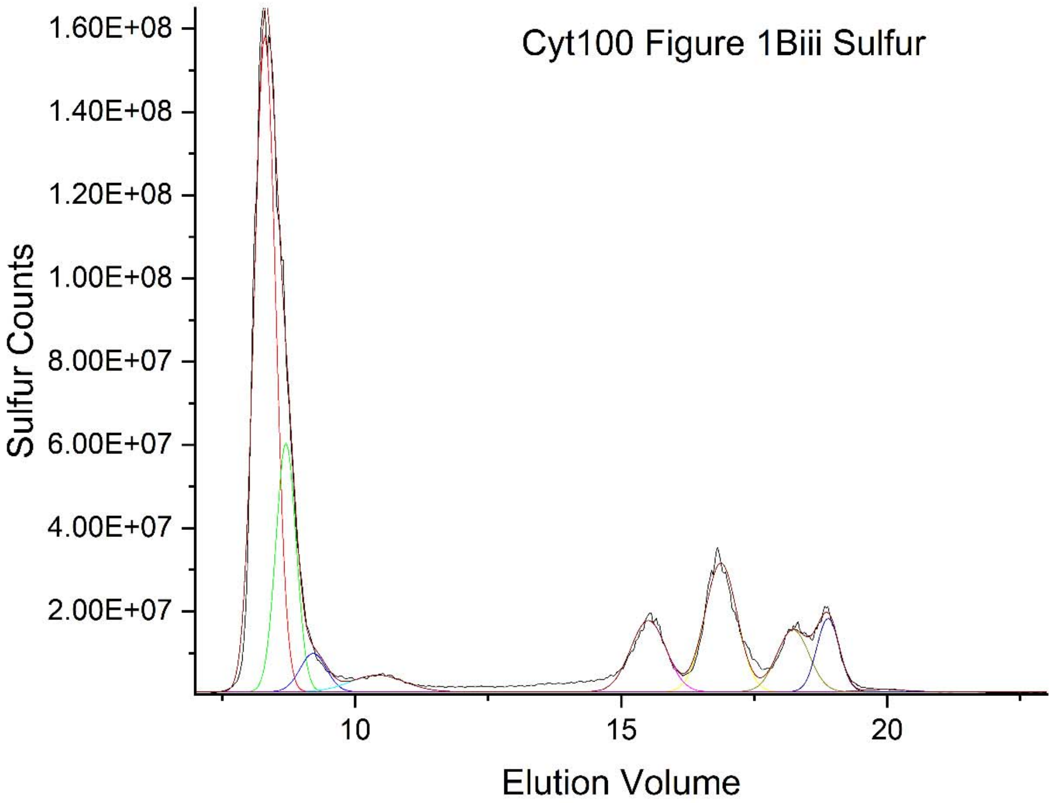
Cyt100 Figure 1Biii Sulfur
Sulfur Counts
1.60E+08
1.40E+08
1.20E+08
1.00E+08
8.00E+07
6.00E+07
4.00E+07
2.00E+07
10
15
20
Elution Volume

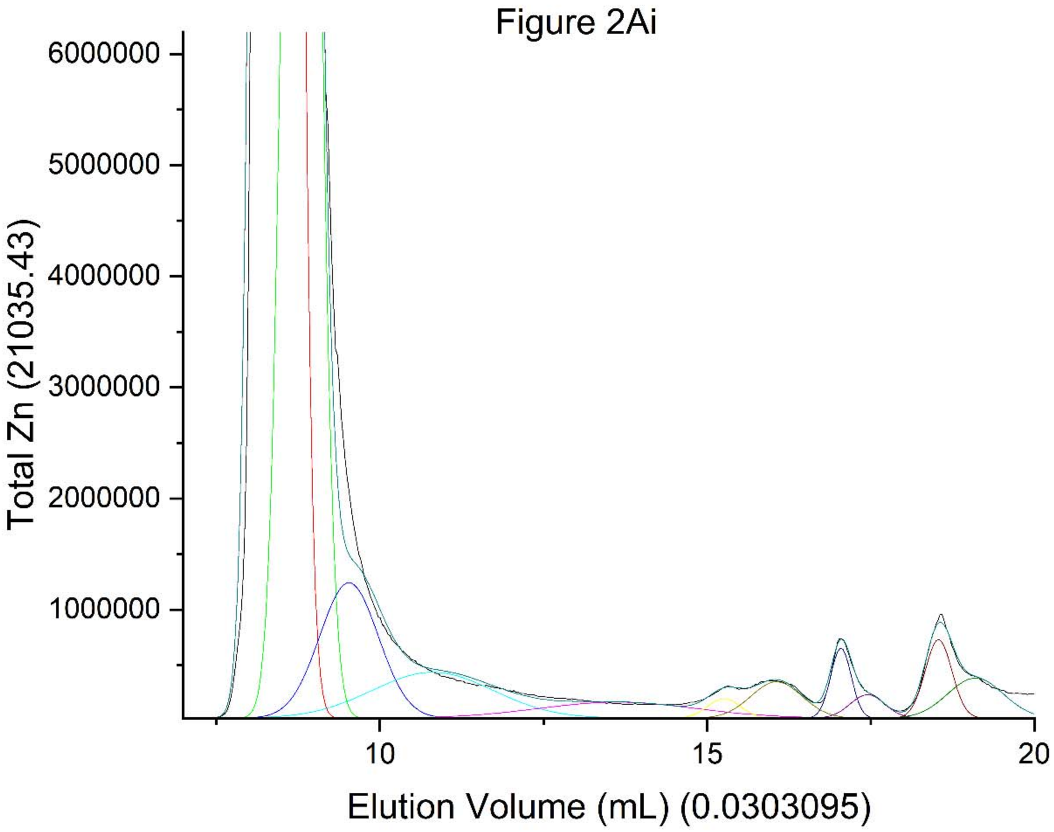

Figure 2Ai
6000000
5000000
4000000
3000000
2000000
1000000
Total Zn (21035.43)
10
15
20
Elution Volume (mL) (0.0303095)

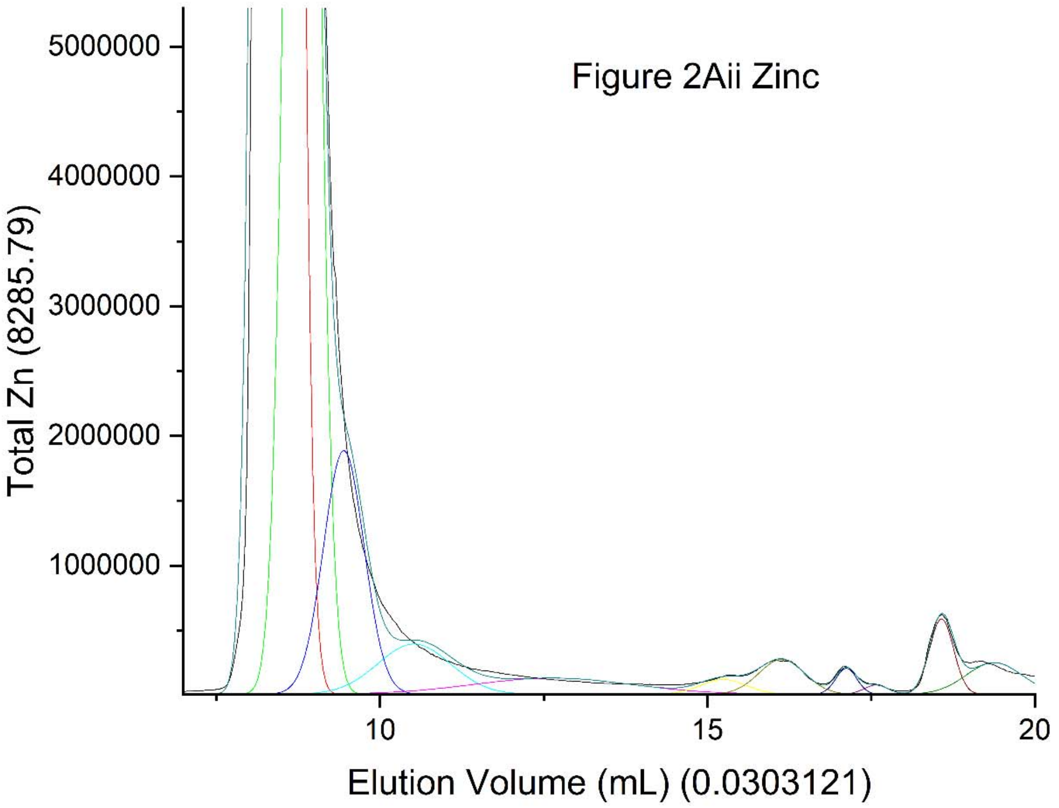
Figure 2Aii Zinc
Total Zn (8285.79)
5000000
4000000
3000000
2000000
1000000
10
15
20
Elution Volume (mL) (0.0303121)

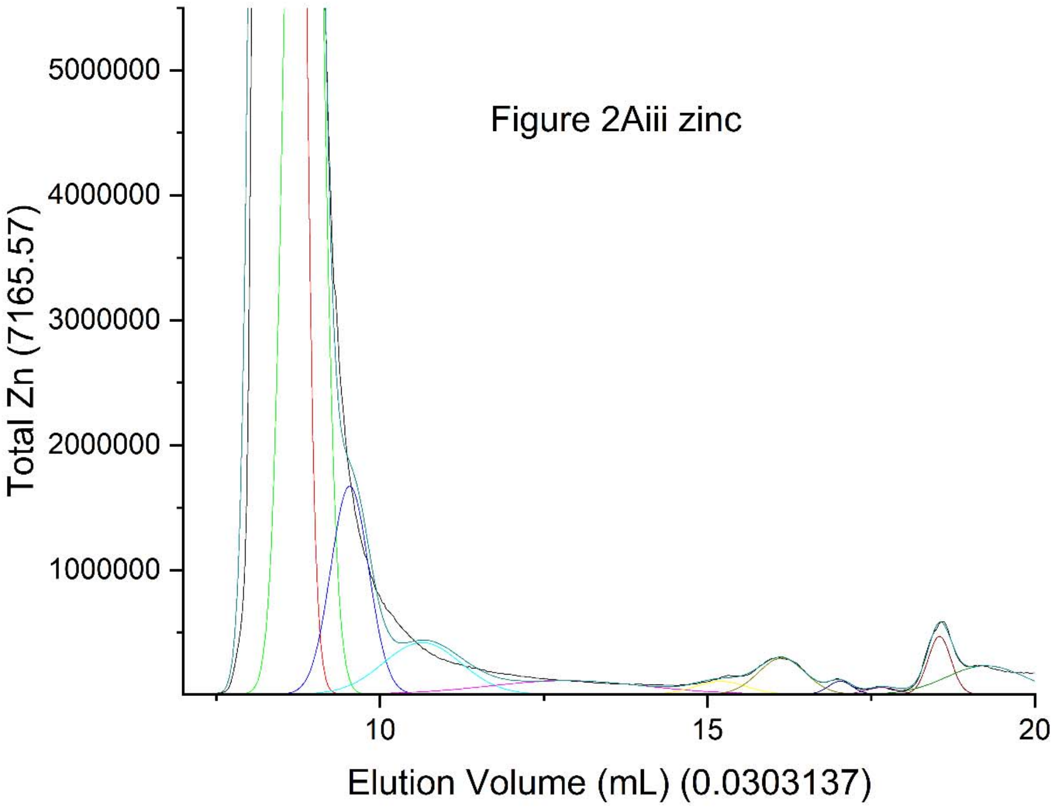
Figure 2Aiii zinc
Total Zn (7165.57)
5000000
4000000
3000000
2000000
1000000
10
15
20
Elution Volume (mL) (0.0303137)

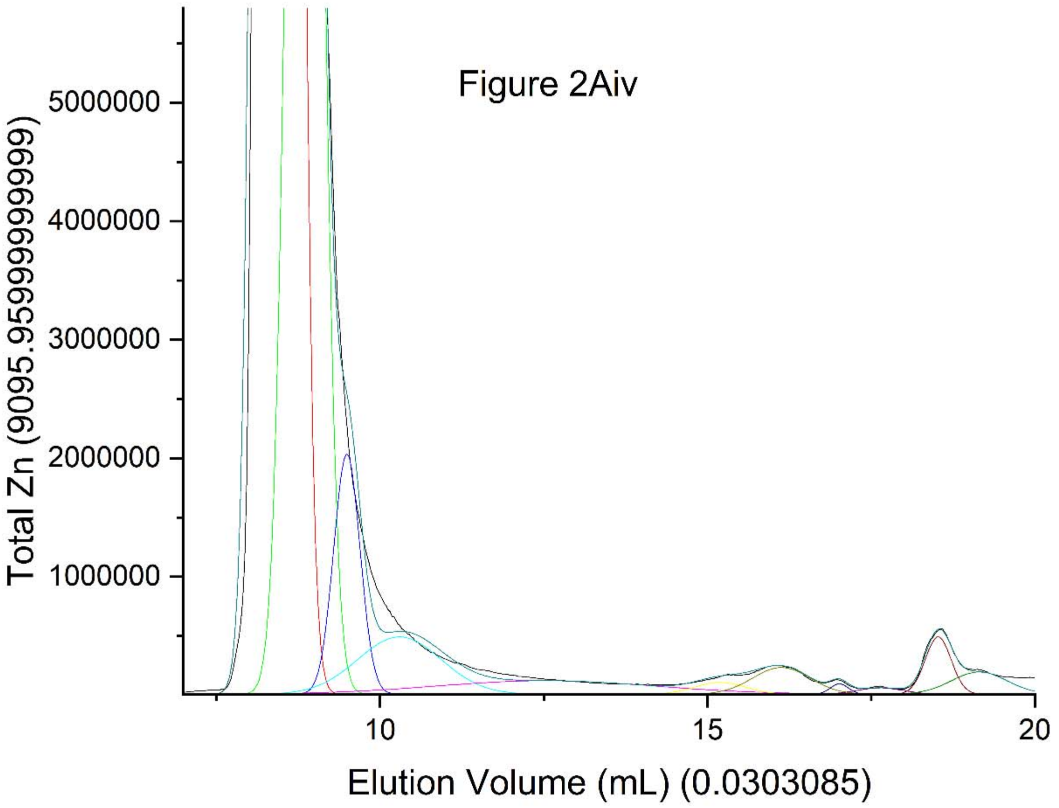
Figure 2Aiv
5000000
4000000
3000000
2000000
1000000
Total Zn (9095.959999999999)
10
15
20
Elution Volume (mL) (0.0303085)

Figure 2i sulfur (not shown)

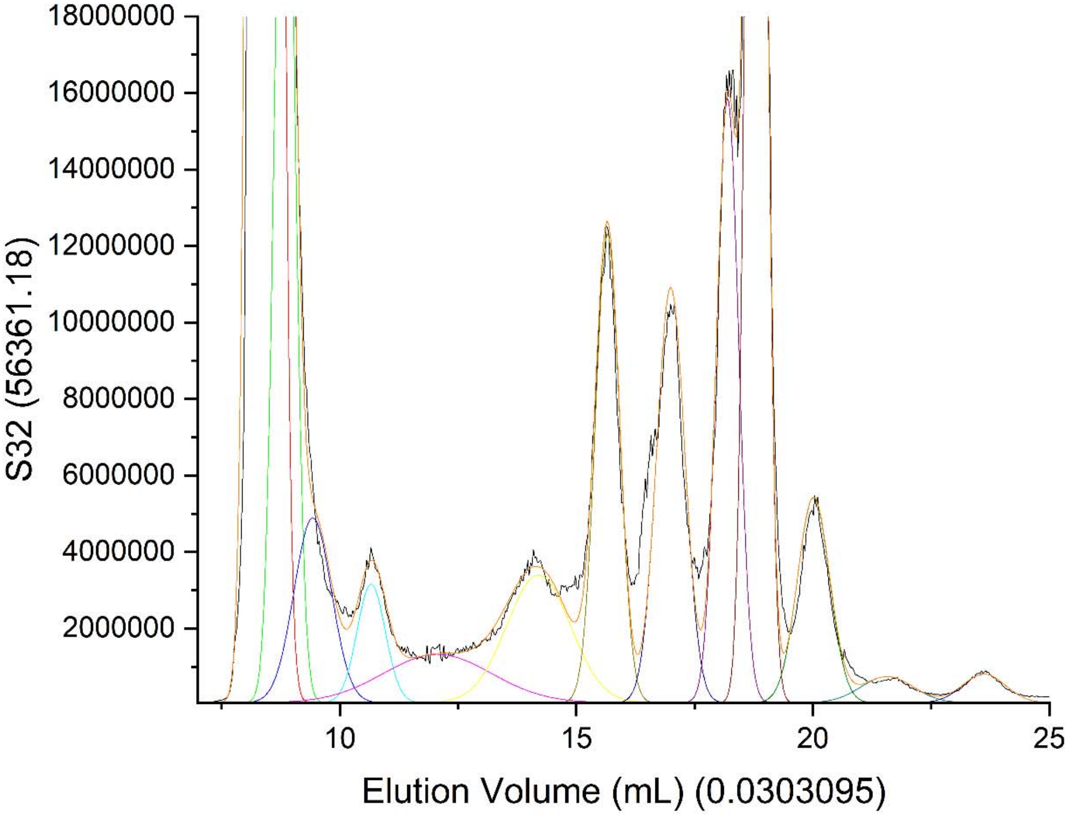

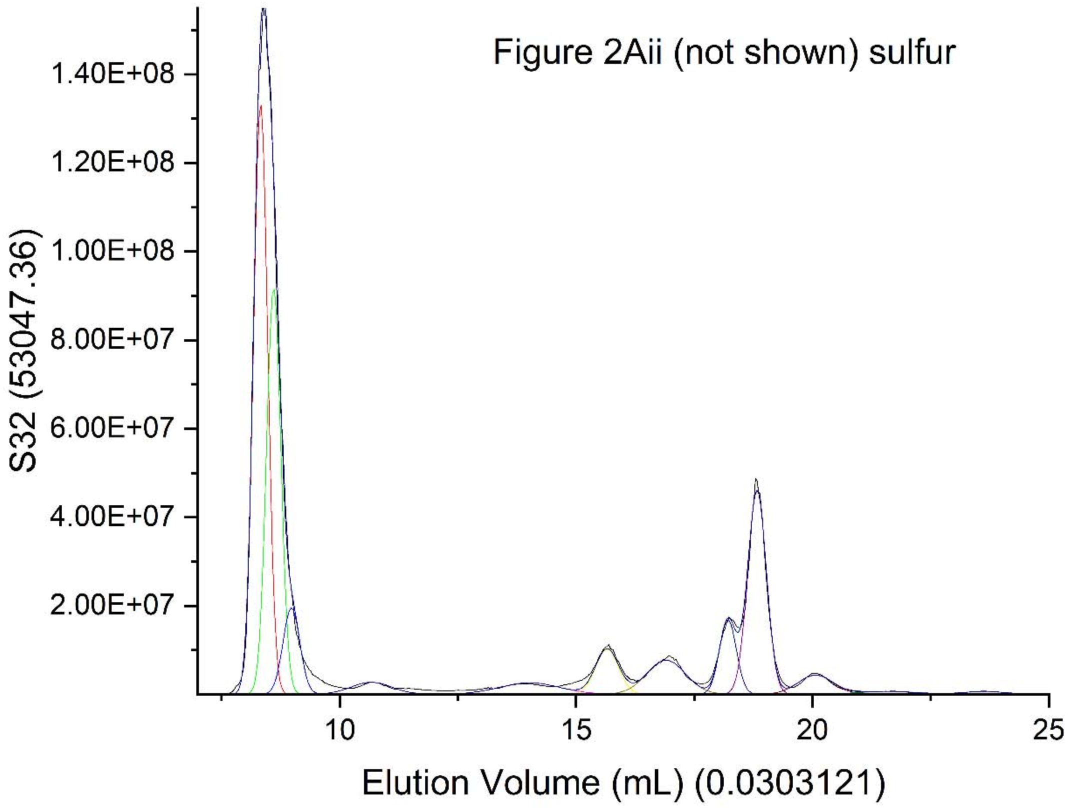
Figure 2Aii (not shown) sulfur
S32 (53047.36)
1.40E+08
1.20E+08
1.00E+08
8.00E+07
6.00E+07
4.00E+07
2.00E+07
10
15
20
25
Elution Volume (mL) (0.0303121)

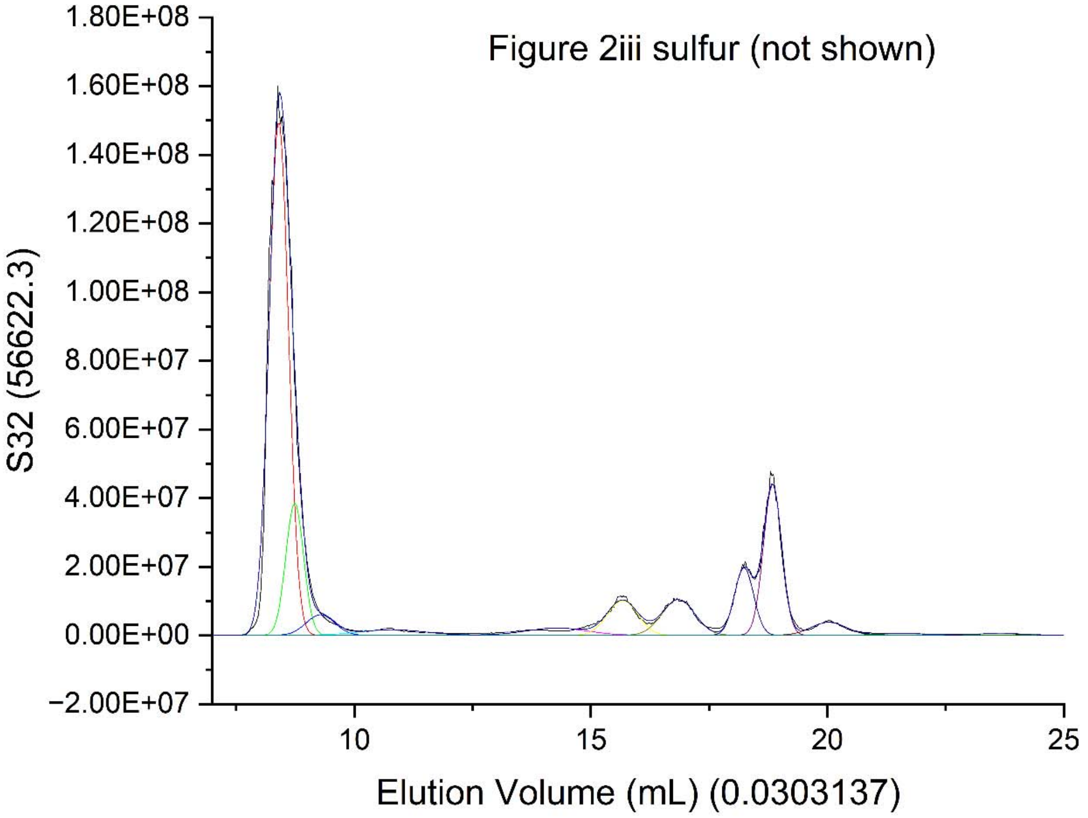
Figure 2iii sulfur (not shown)
1.80E+08
1.60E+08
1.40E+08
1.20E+08
1.00E+08
8.00E+07
6.00E+07
4.00E+07
2.00E+07
0.00E+00
−2.00E+07
S32 (56622.3)
10
15
20
25
Elution Volume (mL) (0.0303137)

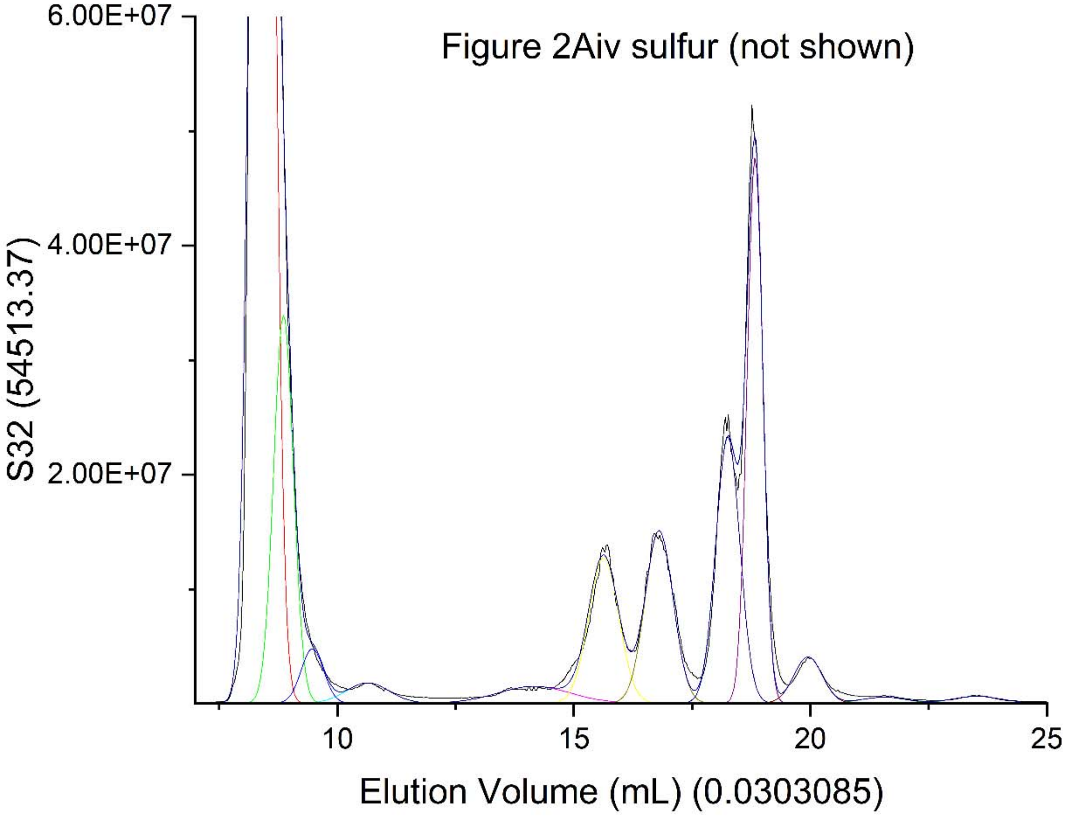
Figure 2Aiv sulfur (not shown)
6.00E+07
4.00E+07
2.00E+07
S32 (54513.37)
10
15
20
25
Elution Volume (mL) (0.0303085)

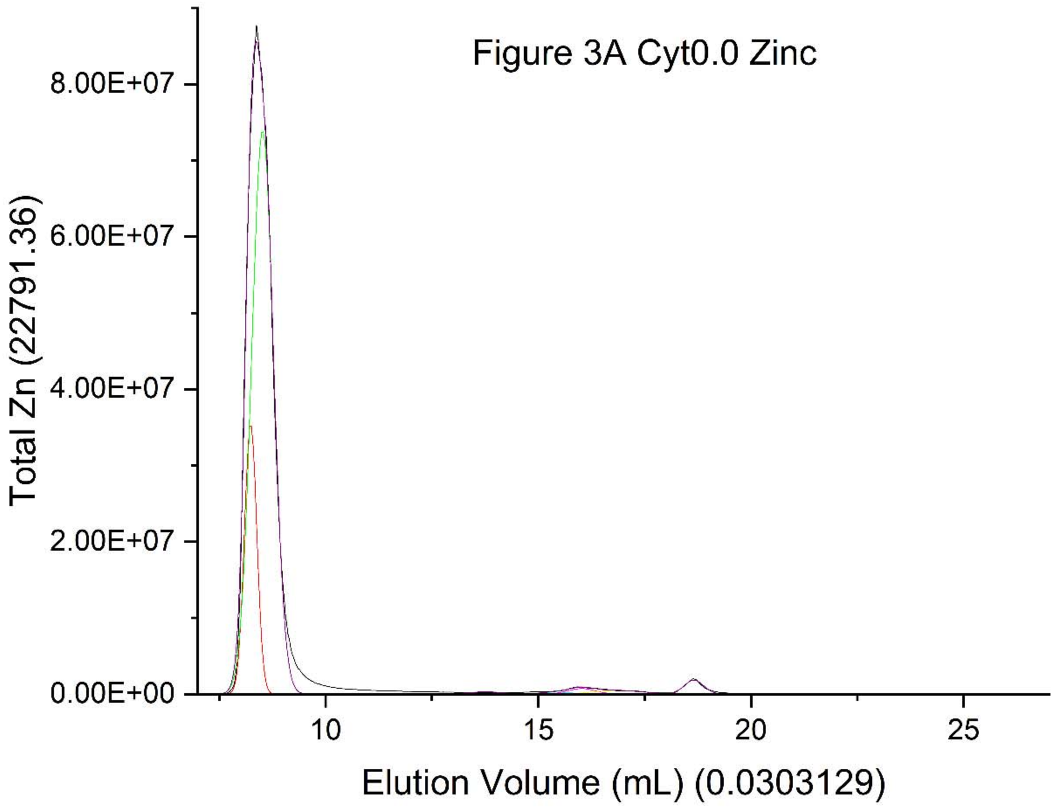
Figure 3A Cyt0.0 Zinc
Total Zn (22791.36)
8.00E+07
6.00E+07
4.00E+07
2.00E+07
0.00E+00
10
15
20
25
Elution Volume (mL) (0.0303129)

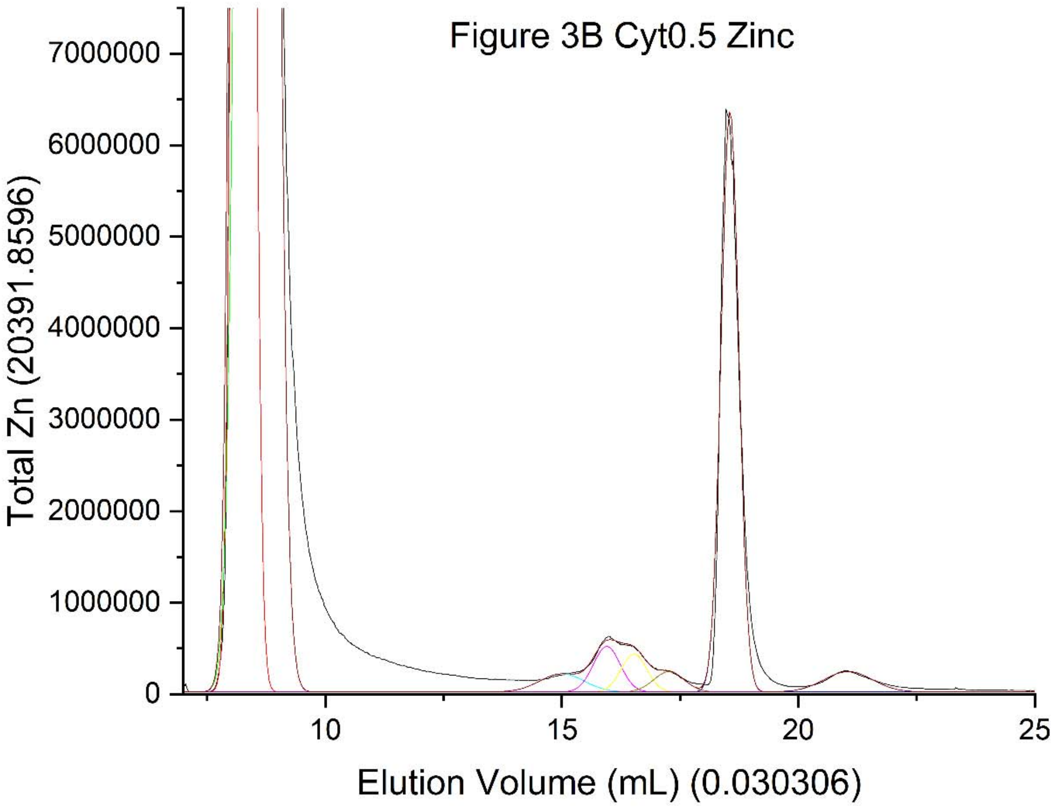
Figure 3B Cyt0.5 Zinc
Total Zn (20391.8596)
7000000
6000000
5000000
4000000
3000000
2000000
1000000
0
10
15
20
25
Elution Volume (mL) (0.030306)

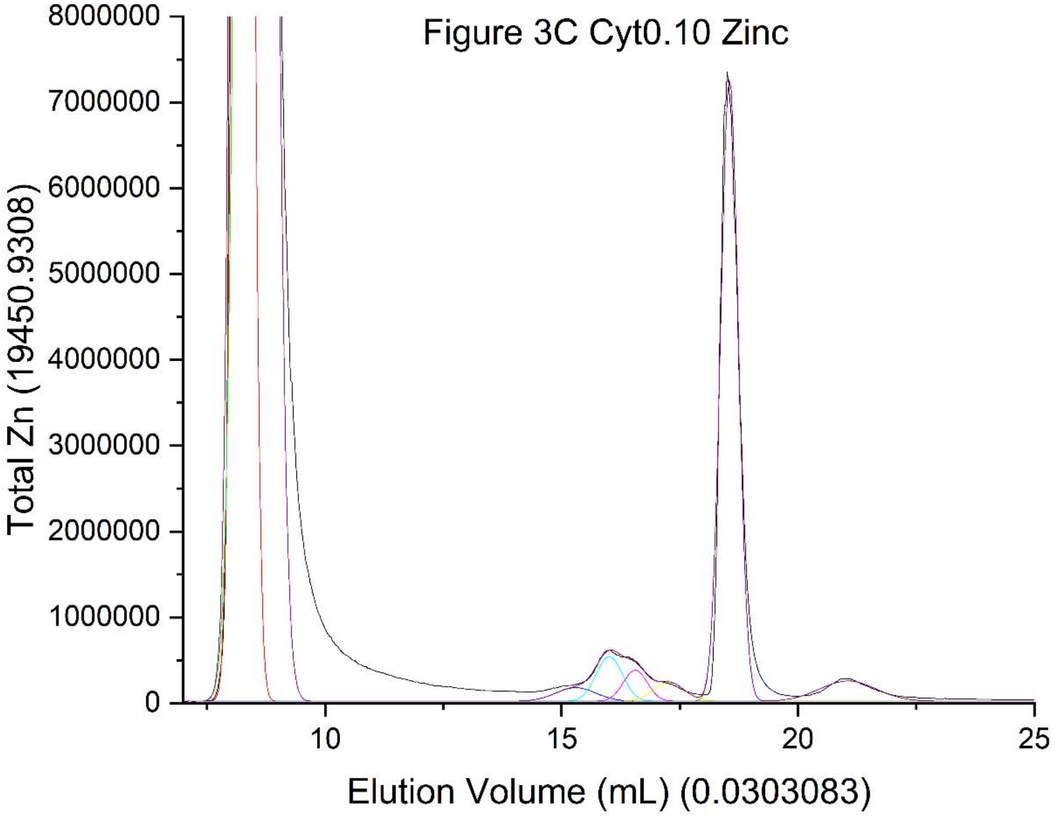
Figure 3C Cyt0.10 Zinc
Total Zn (19450.9308)
8000000
7000000
6000000
5000000
4000000
3000000
2000000
1000000
0
10
15
20
25
Elution Volume (mL) (0.0303083)

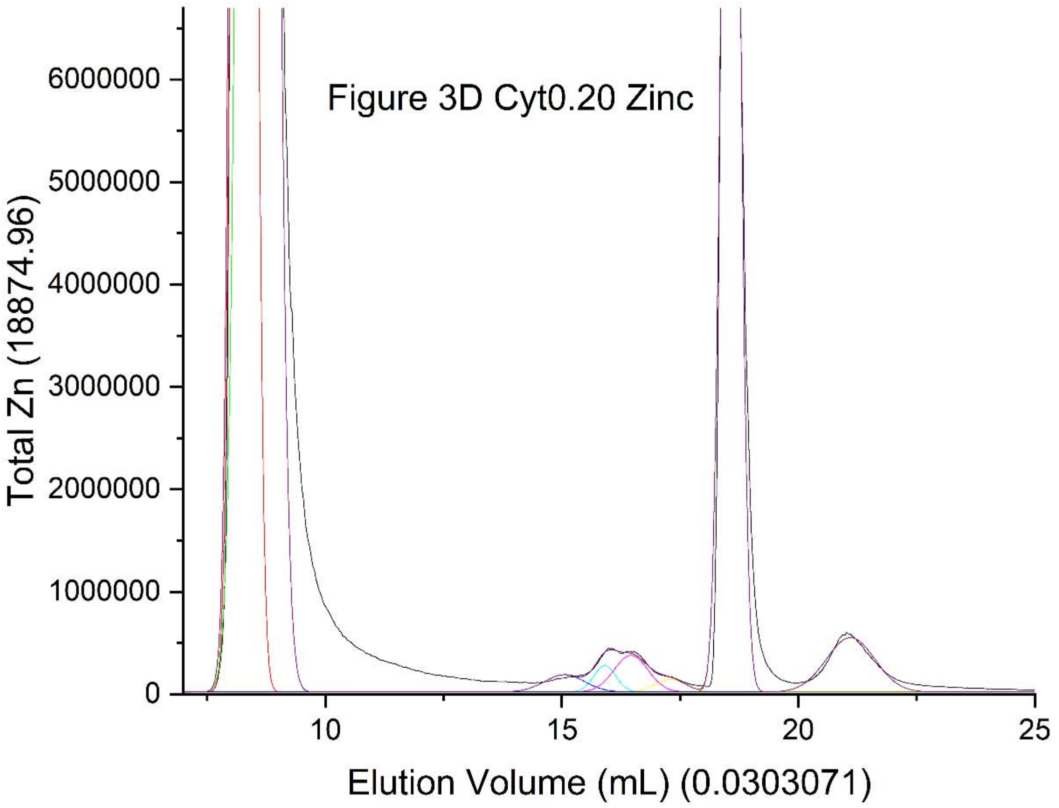

Figure 3D Cyt0.20 Zinc
Total Zn (18874.96)
6000000
5000000
4000000
3000000
2000000
1000000
0
10
15
20
25
Elution Volume (mL) (0.0303071)

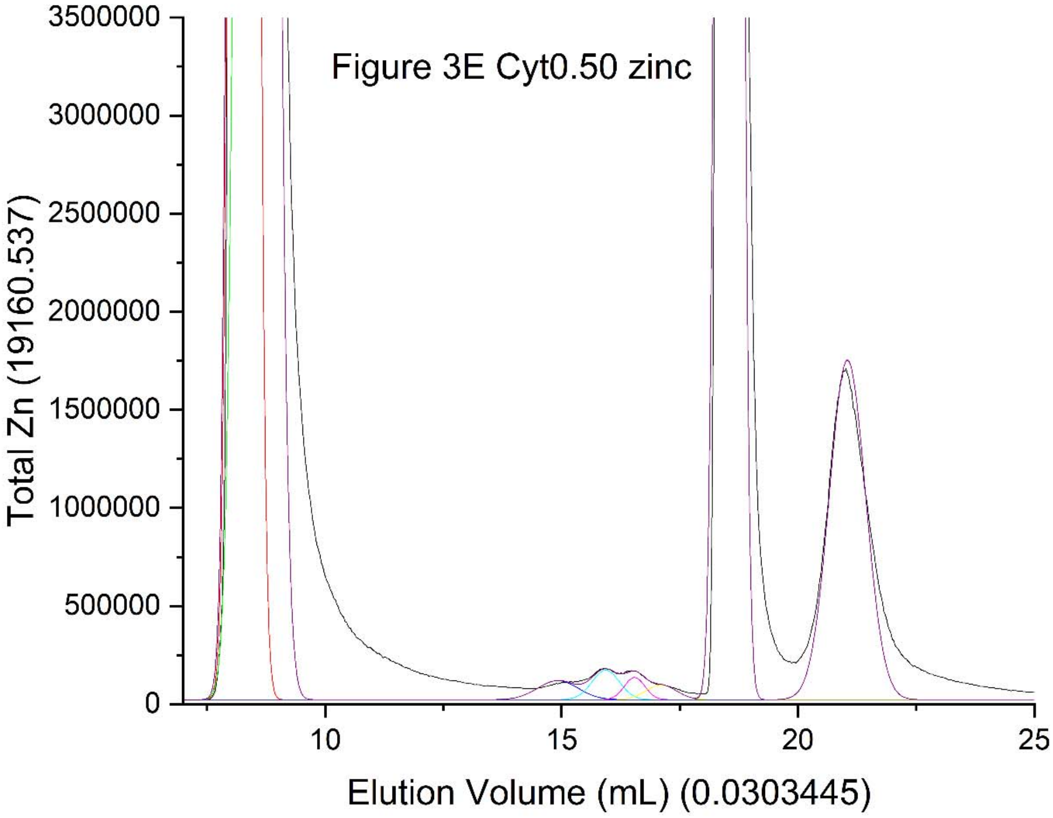
Figure 3E Cyt0.50 zinc
3500000
3000000
2500000
2000000
1500000
1000000
500000
0
Total Zn (19160.537)
10
15
20
25
Elution Volume (mL) (0.0303445)

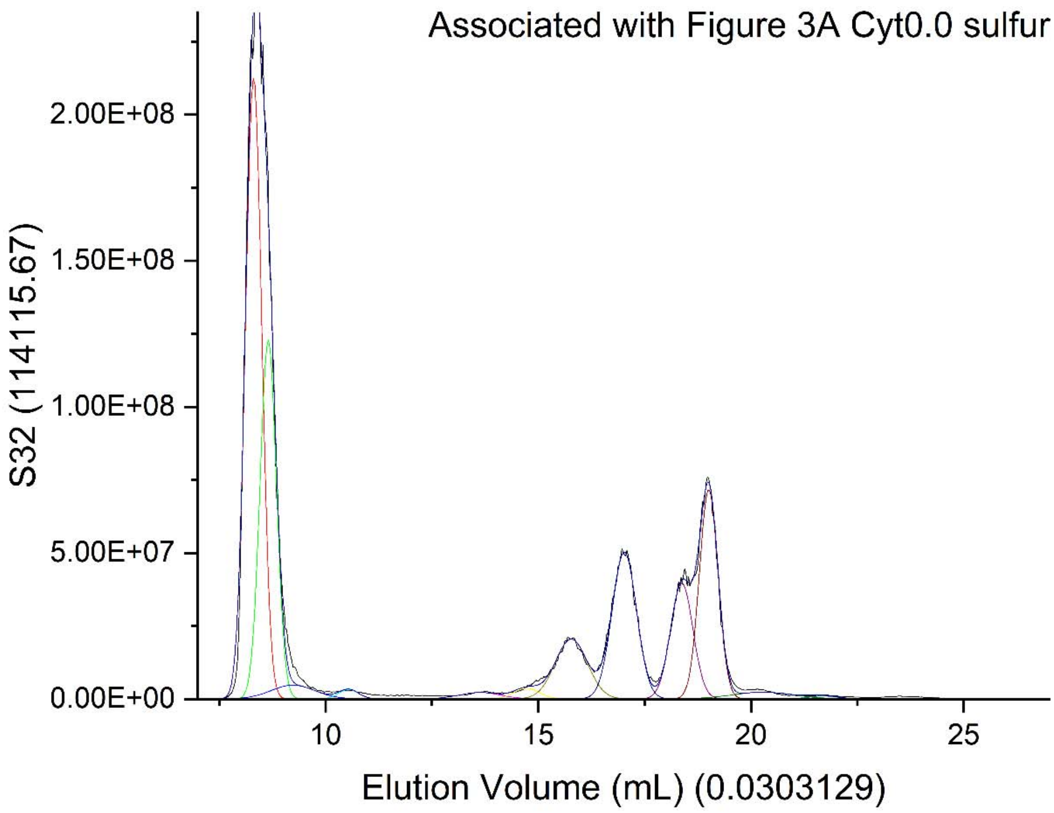
Associated with Figure 3A Cyt0.0 sulfur
S32 (114115.67)
2.00E+08
1.50E+08
1.00E+08
5.00E+07
0.00E+00
10
15
20
25
Elution Volume (mL) (0.0303129)

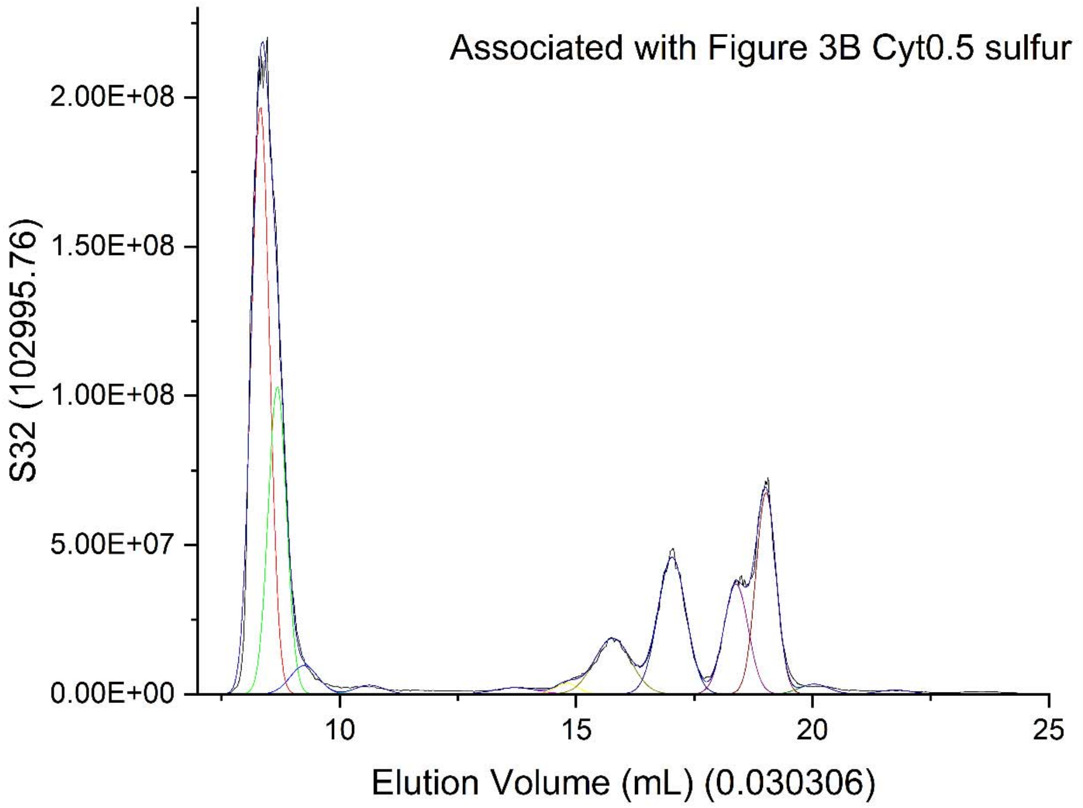

Associated with Figure 3B Cyt0.5 sulfur
S32 (102995.76)
2.00E+08
1.50E+08
1.00E+08
5.00E+07
0.00E+00
10
15
20
25
Elution Volume (mL) (0.030306)

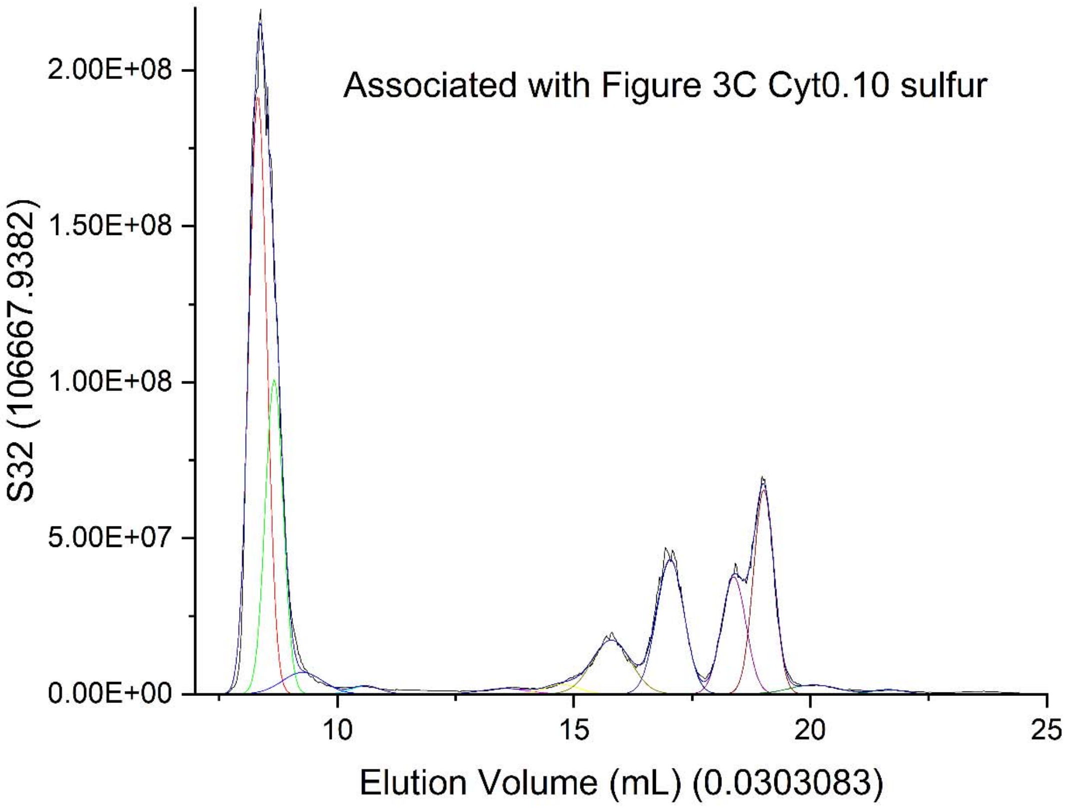
Associated with Figure 3C Cyt0.10 sulfur
S32 (106667.9382)
2.00E+08
1.50E+08
1.00E+08
5.00E+07
0.00E+00
10
15
20
25
Elution Volume (mL) (0.0303083)

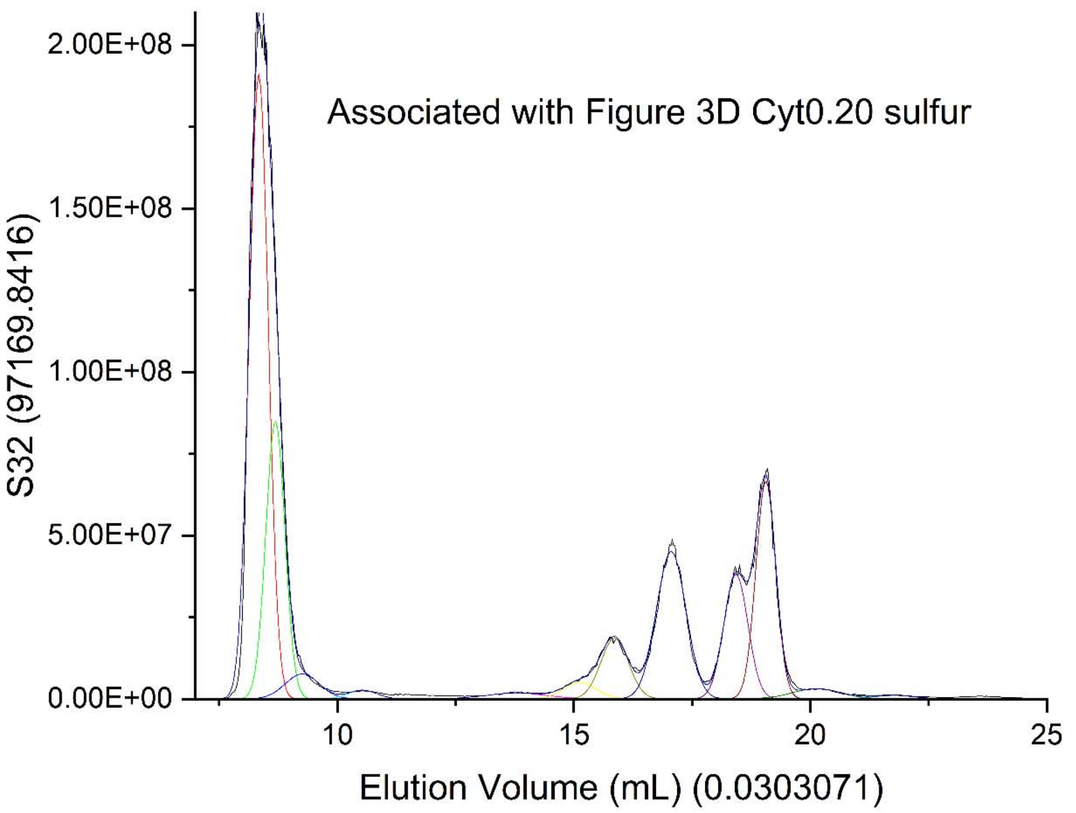

Associated with Figure 3D Cyt0.20 sulfur
S32 (97169.8416)
2.00E+08
1.50E+08
1.00E+08
5.00E+07
0.00E+00
10
15
20
25
Elution Volume (mL) (0.0303071)

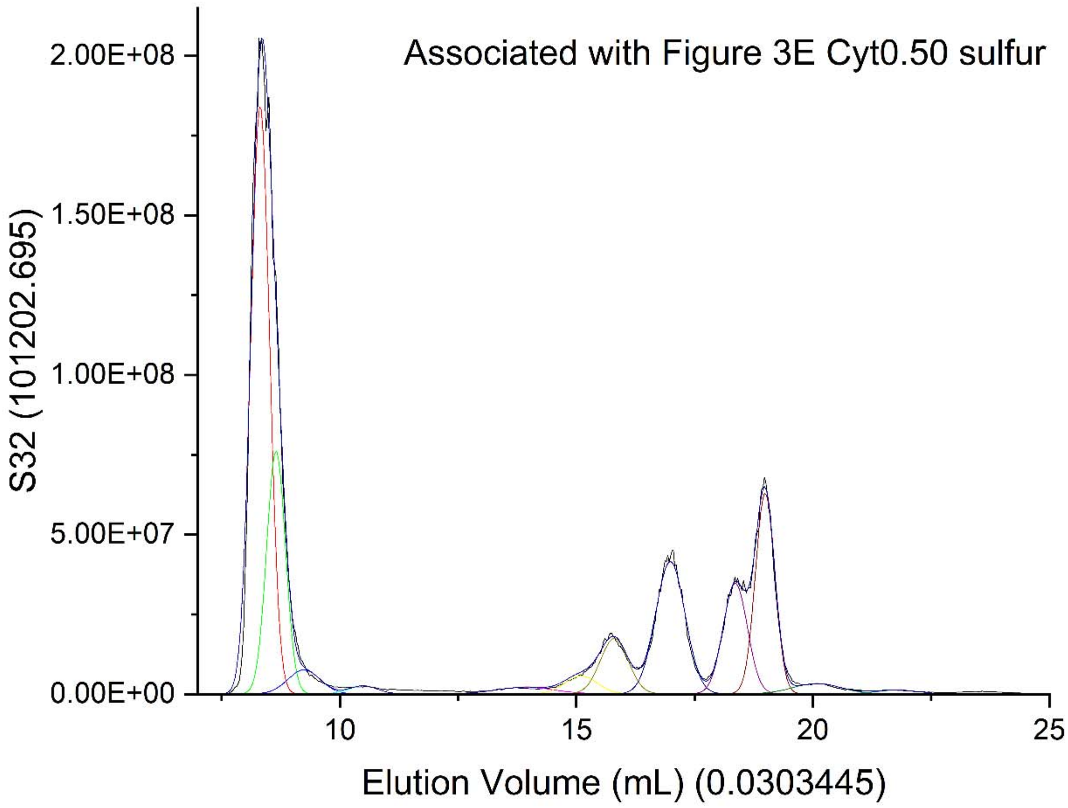
Associated with Figure 3E Cyt0.50 sulfur
S32 (101202.695)
2.00E+08
1.50E+08
1.00E+08
5.00E+07
0.00E+00
10
15
20
25
Elution Volume (mL) (0.0303445)

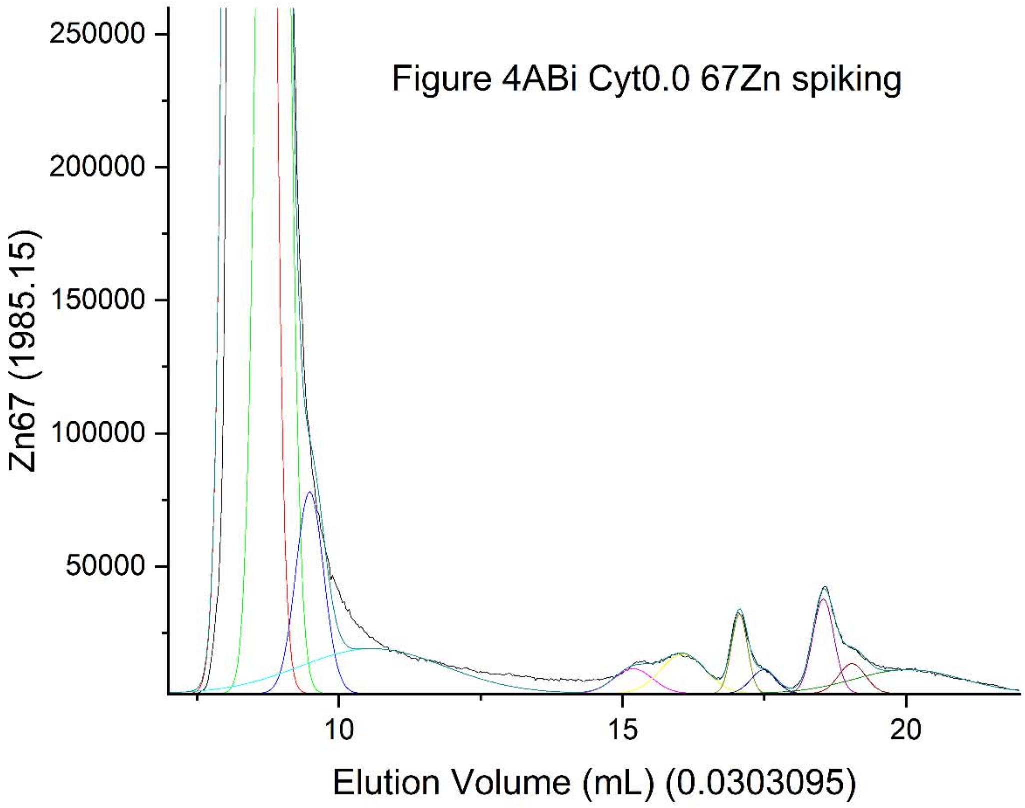
Figure 4ABi Cyt0.0 67Zn spiking
250000
200000
150000
100000
50000
Zn67 (1985.15)
10
15
20
Elution Volume (mL) (0.0303095)

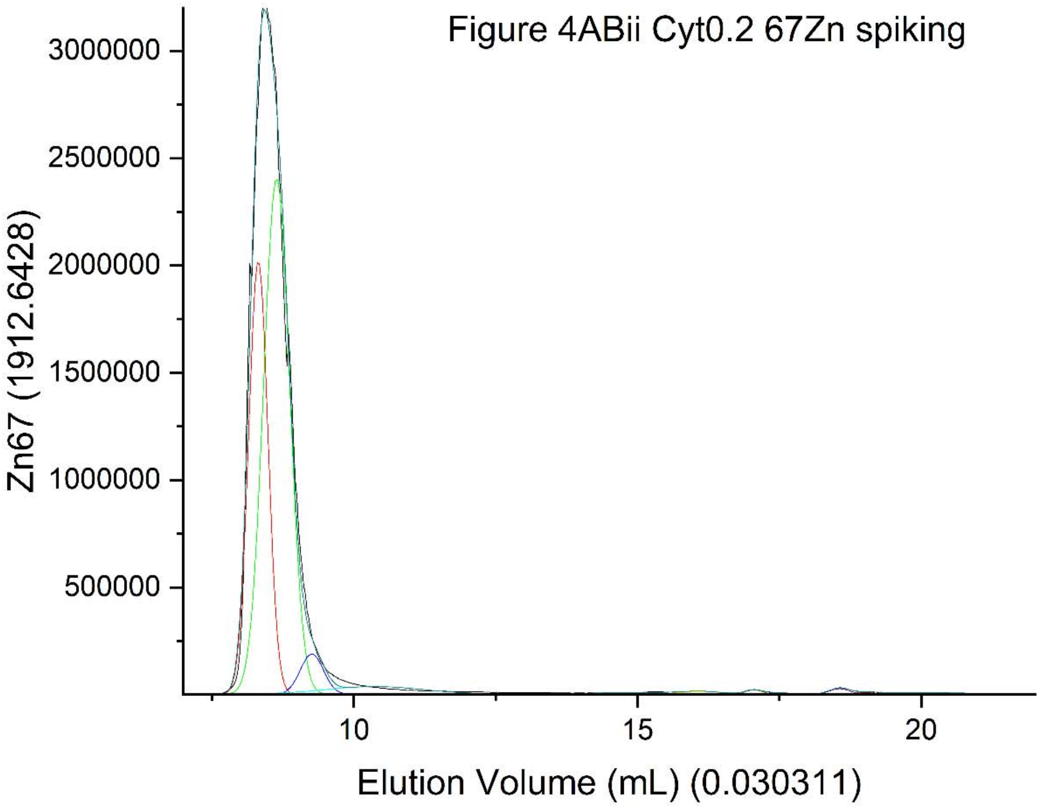
Figure 4ABii Cyt0.2 67Zn spiking
3000000
2500000
2000000
1500000
1000000
500000
Zn67 (1912.6428)
10
15
20
Elution Volume (mL) (0.030311)

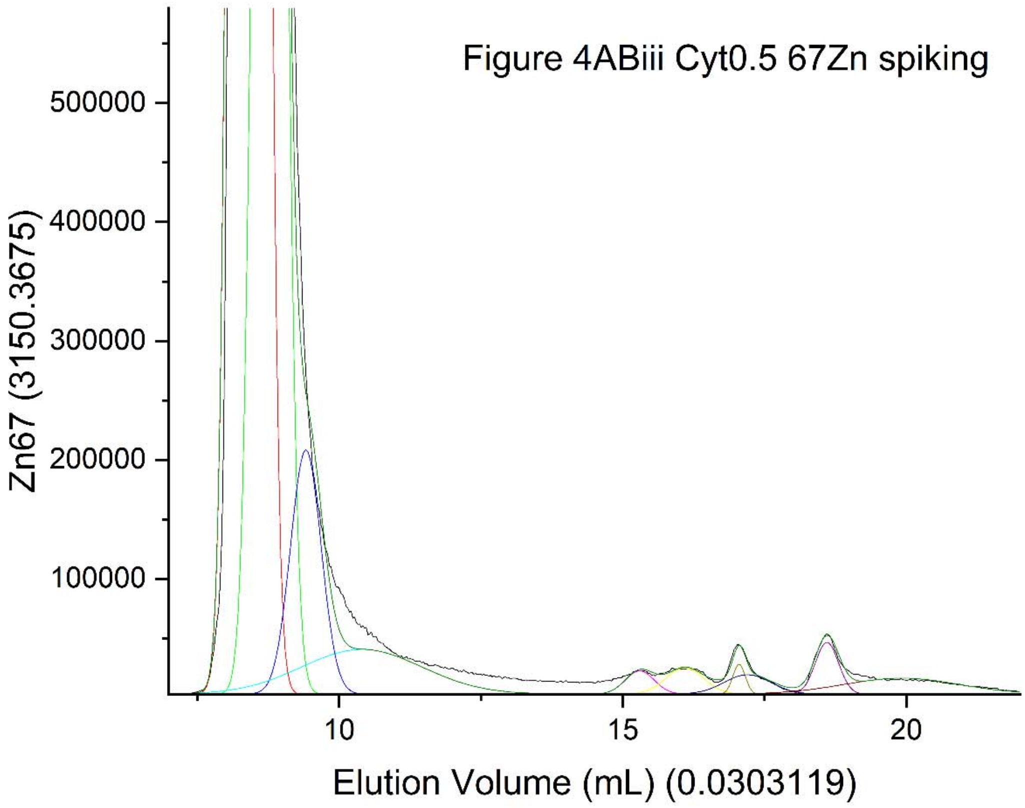
Figure 4ABiii Cyt0.5 67Zn spiking
Zn67 (3150.3675)
500000
400000
300000
200000
100000
10
15
20
Elution Volume (mL) (0.0303119)

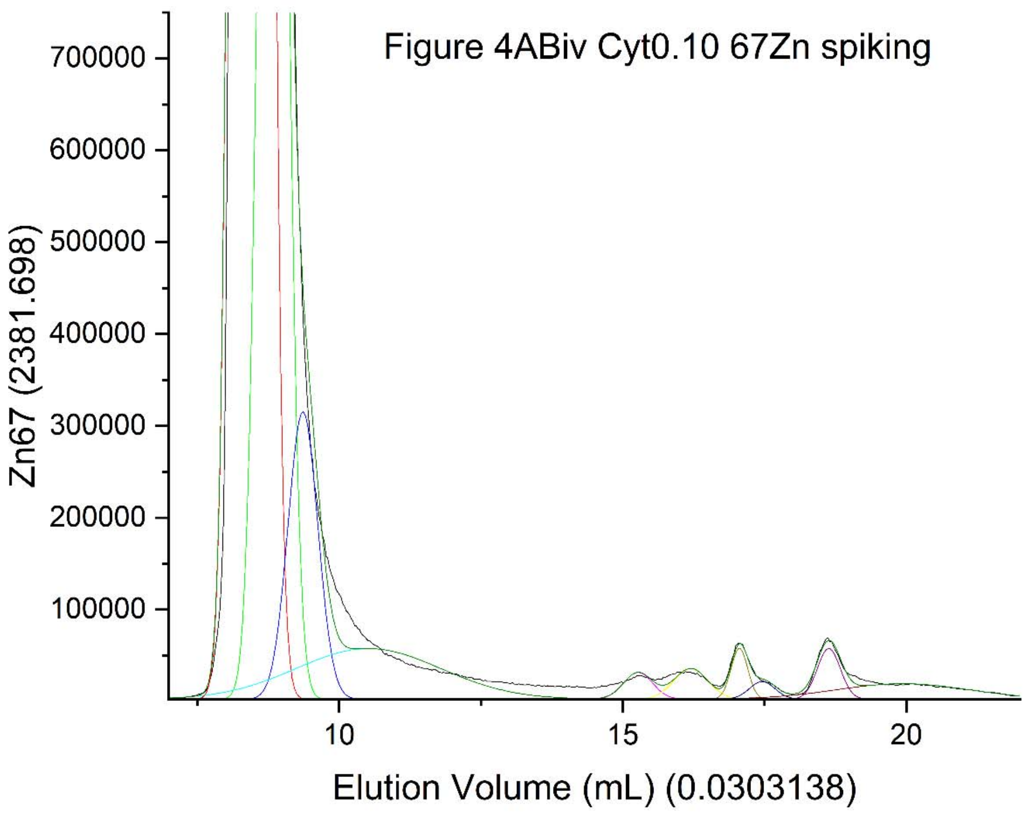

Figure 4ABiv Cyt0.10 67Zn spiking
Zn67 (2381.698)
700000
600000
500000
400000
300000
200000
100000
10
15
20
Elution Volume (mL) (0.0303138)

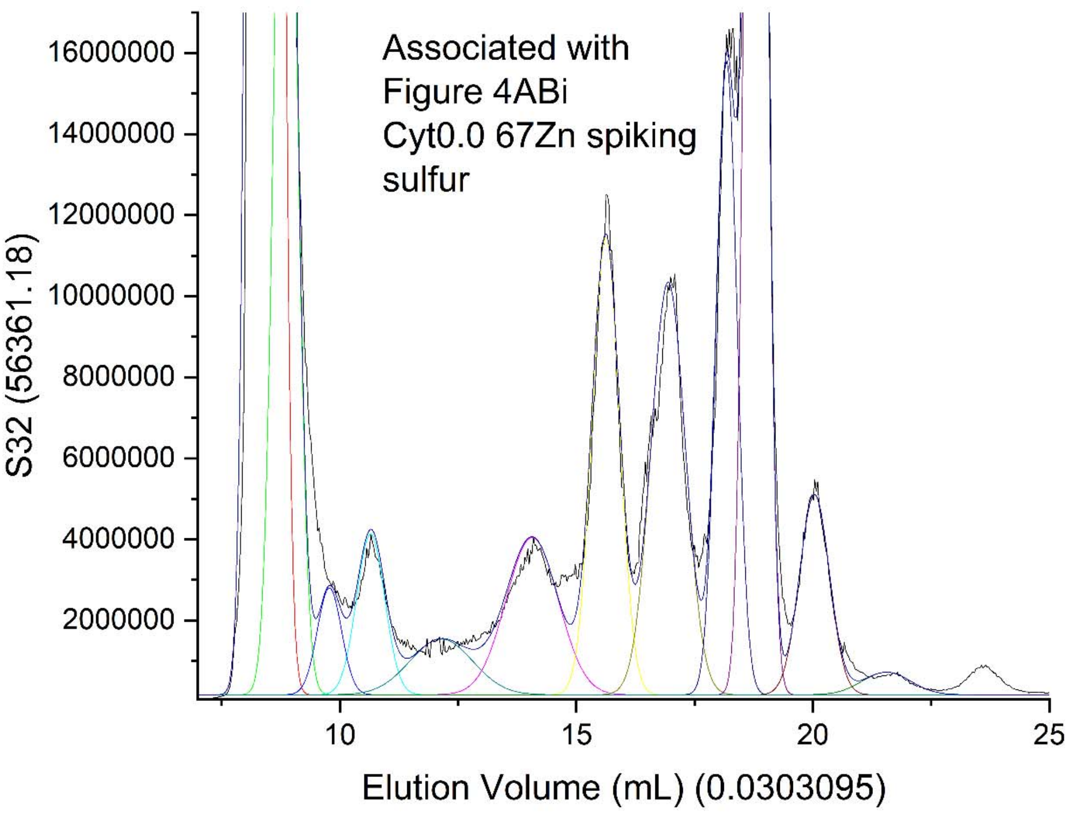
Associated with
Figure 4ABi
Cyt0.0 67Zn spiking
sulfur
S32 (56361.18)
16000000
14000000
12000000
10000000
8000000
6000000
4000000
2000000
10
15
20
25
Elution Volume (mL) (0.0303095)

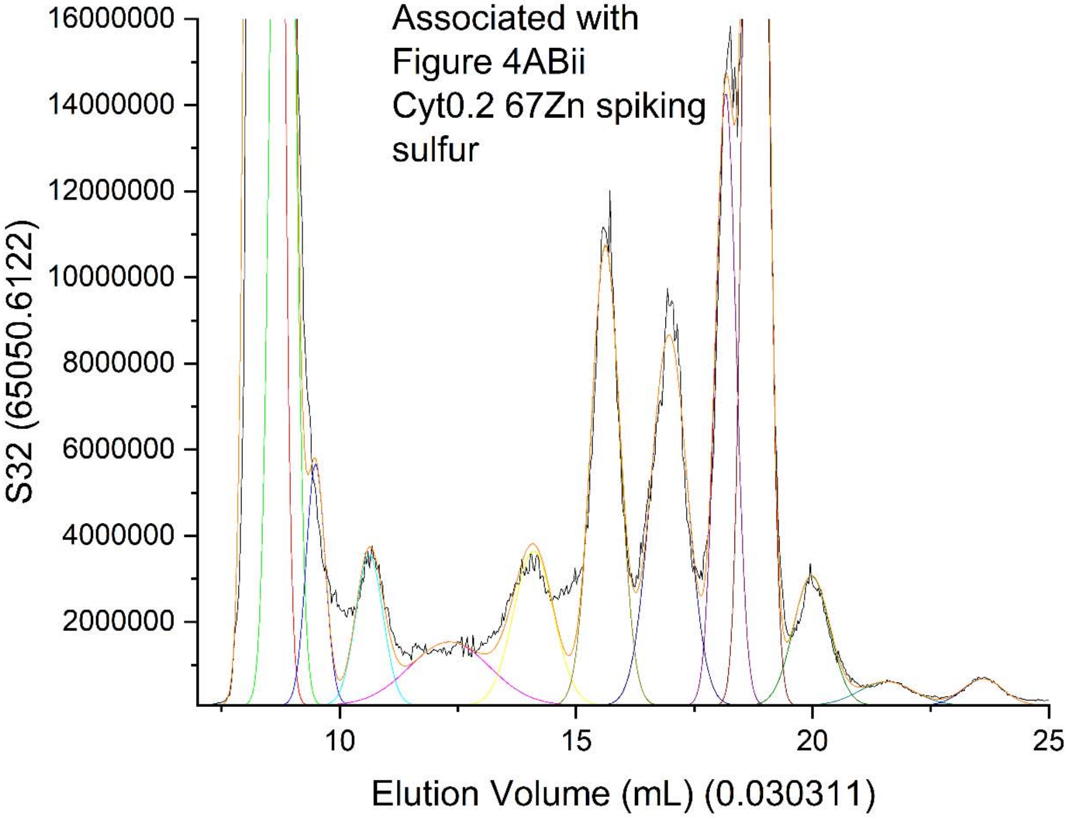
Associated with
Figure 4ABii
Cyt0.2 67Zn spiking
sulfur
S32 (65050.6122)
Elution Volume (mL) (0.030311)
16000000
14000000
12000000
10000000
8000000
6000000
4000000
2000000
10
15
20
25

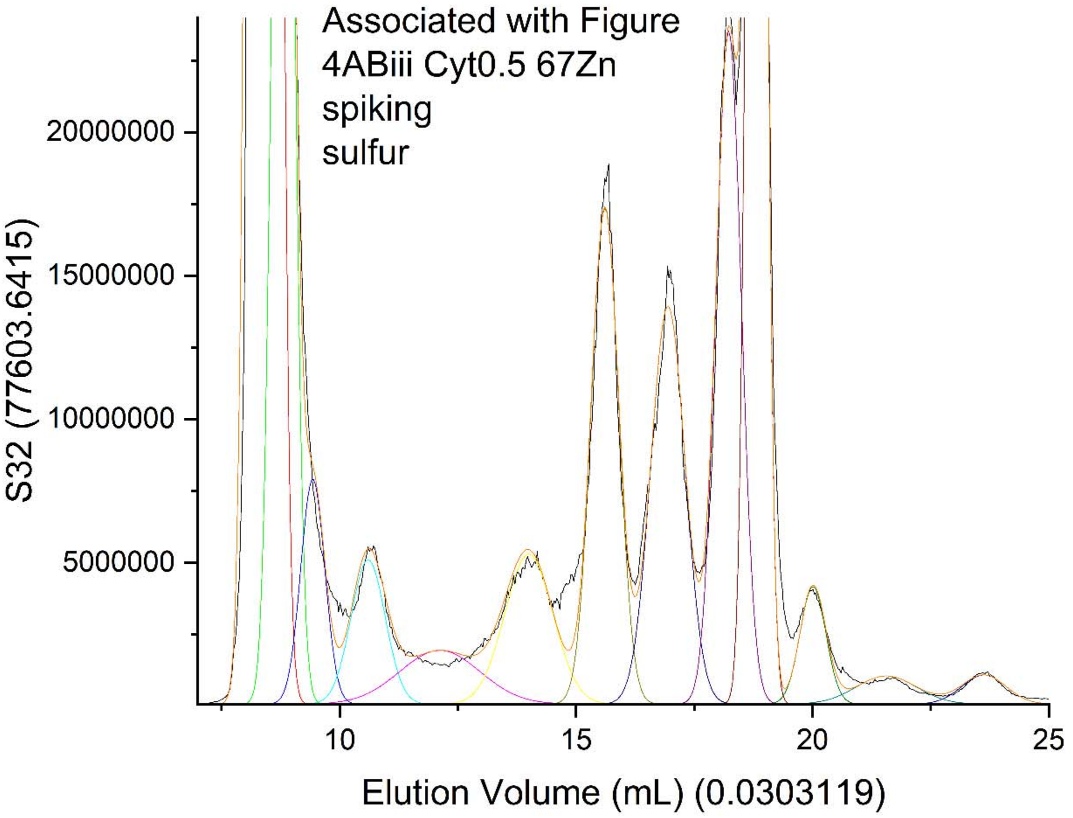
Associated with Figure
4ABiii Cyt0.5 67Zn
spiking
sulfur
S32 (77603.6415)
20000000
15000000
10000000
5000000
10
15
20
25
Elution Volume (mL) (0.0303119)

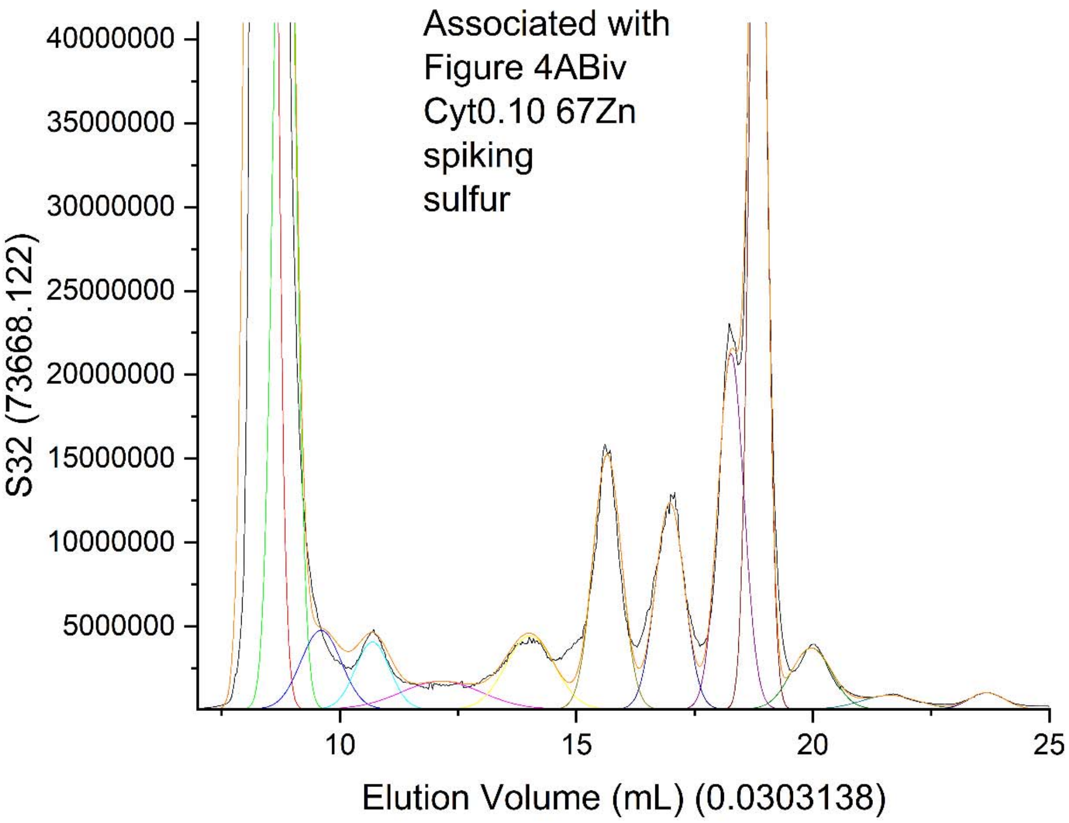
Associated with
Figure 4ABiv
Cyt0.10 67Zn
spiking
sulfur
40000000
35000000
30000000
25000000
20000000
15000000
10000000
5000000
S32 (73668.122)
10
15
20
25
Elution Volume (mL) (0.0303138)

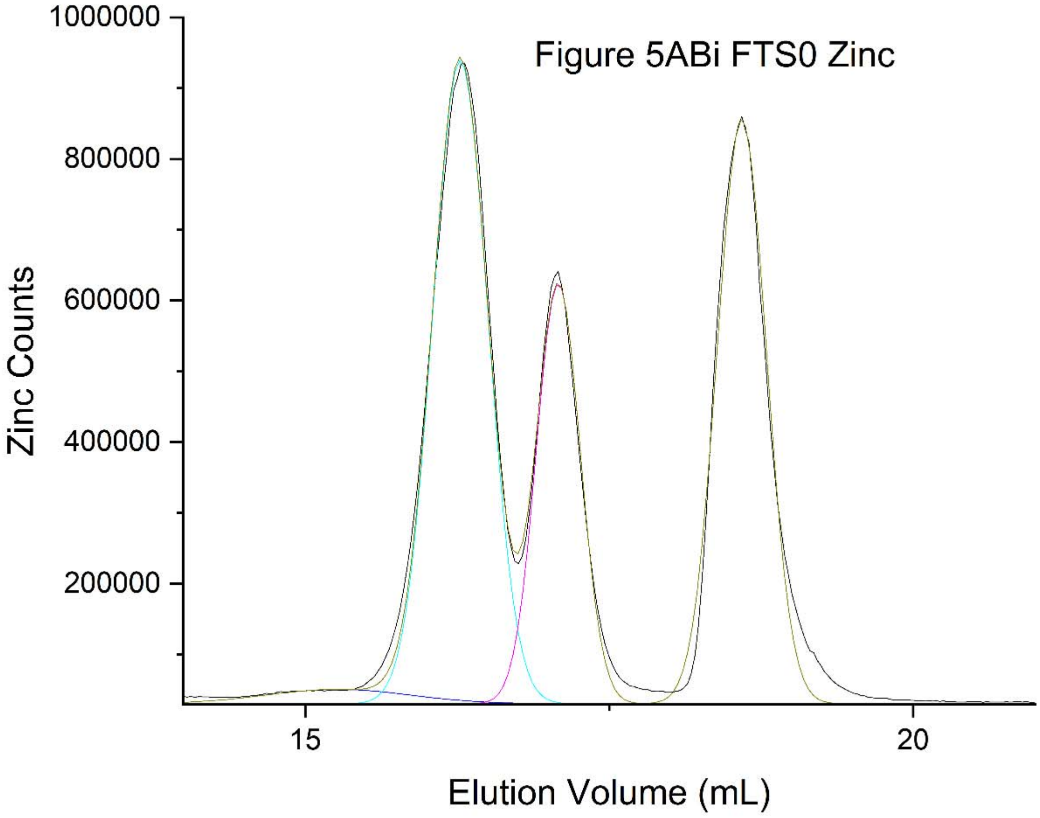
Figure 5ABi FTS0 Zinc
1000000
800000
600000
400000
200000
Zinc Counts
15
20
Elution Volume (mL)

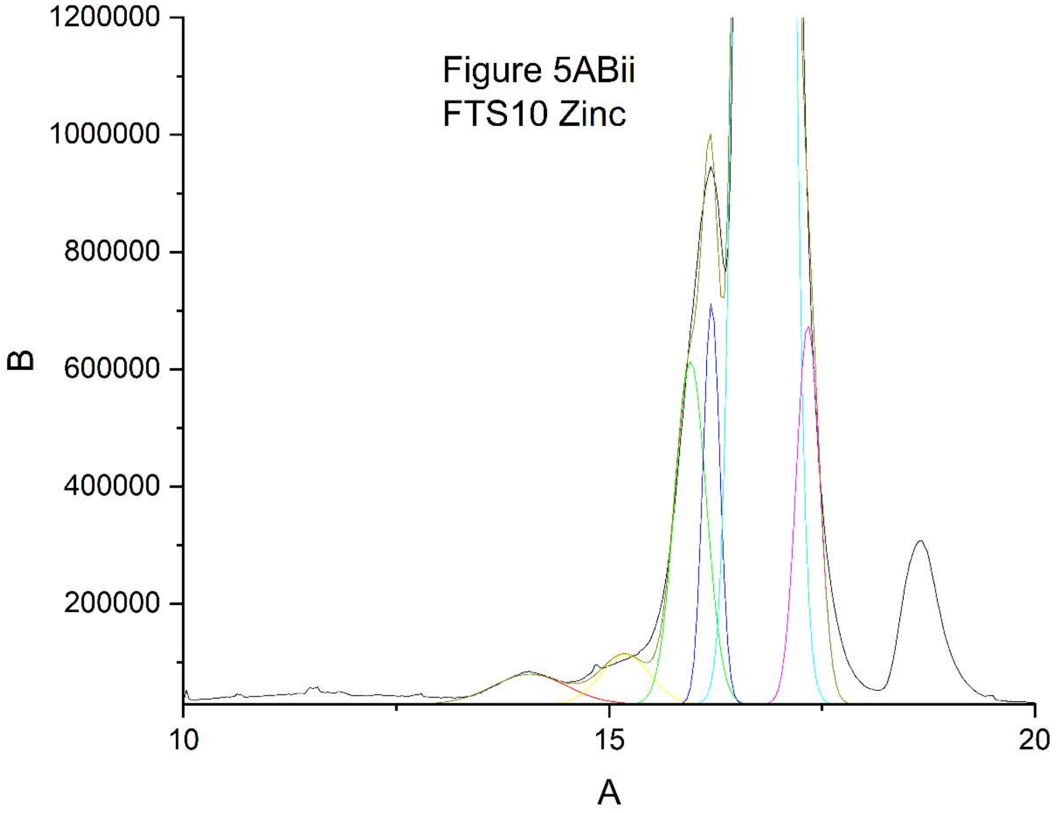
Figure 5ABii
FTS10 Zinc
1200000
1000000
800000
600000
400000
200000
B
10
15
20
A

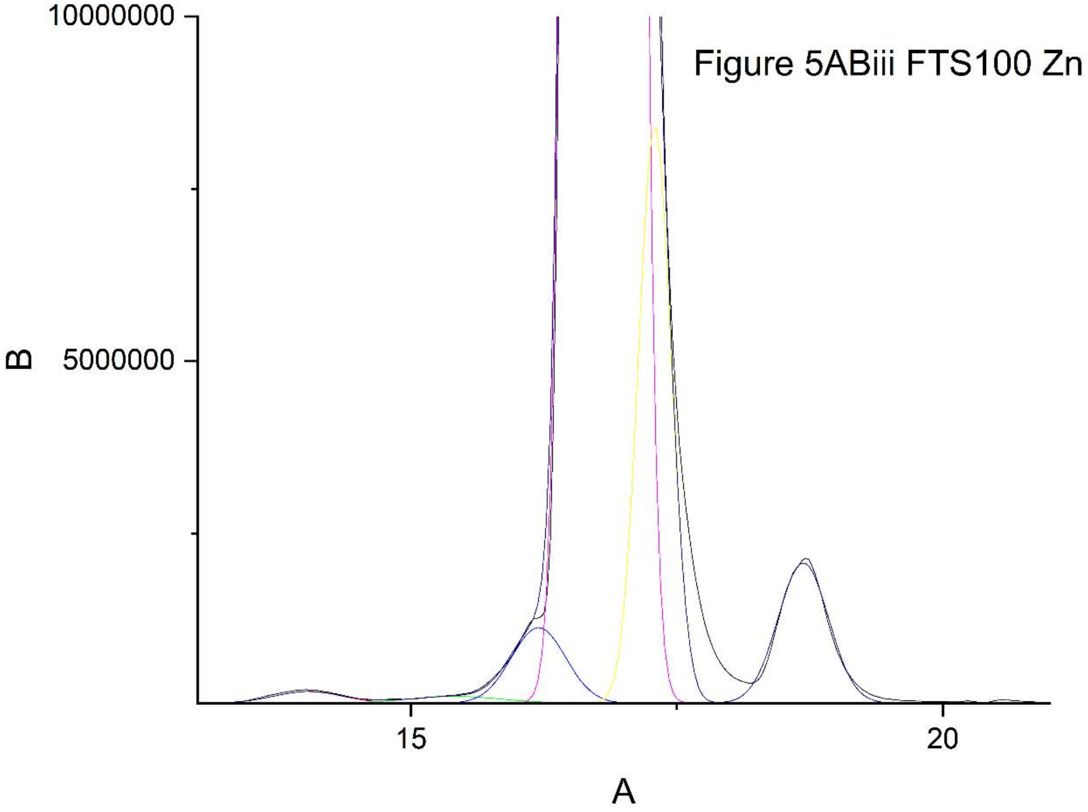

Figure 5ABiii FTS100 Zn
10000000
5000000
B
15
20
A

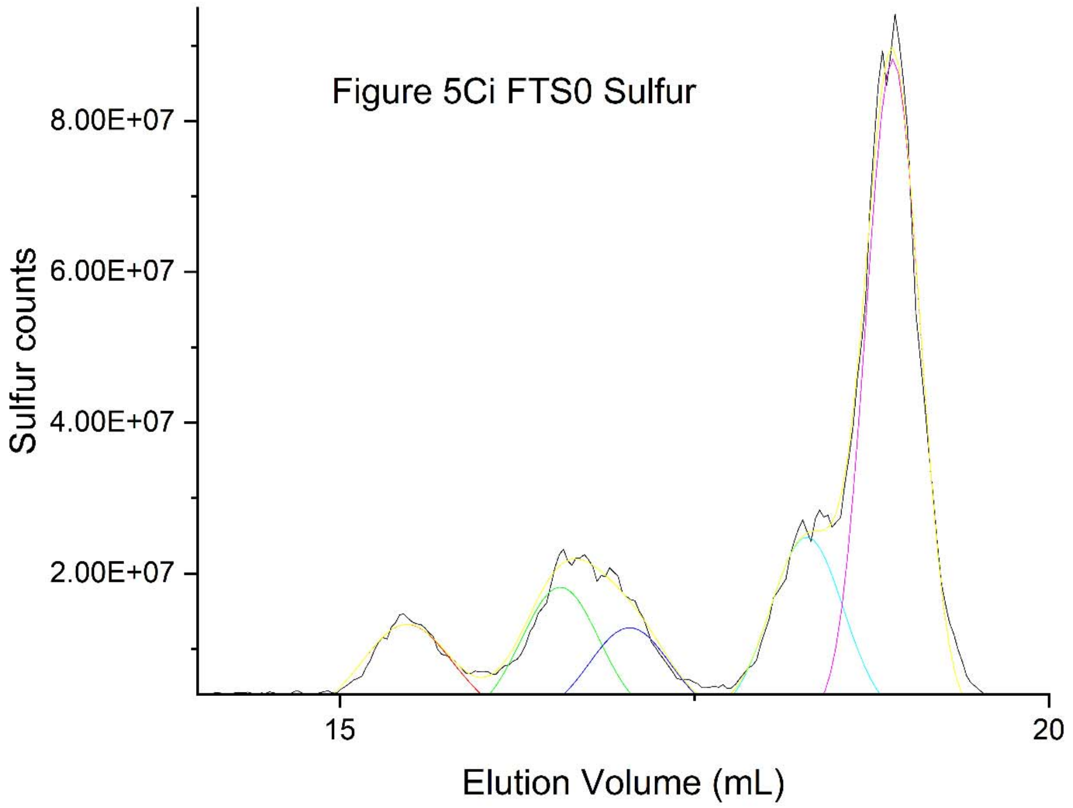

Figure 5Ci FTS0 Sulfur
8.00E+07
6.00E+07
4.00E+07
2.00E+07
Sulfur counts
15
20
Elution Volume (mL)

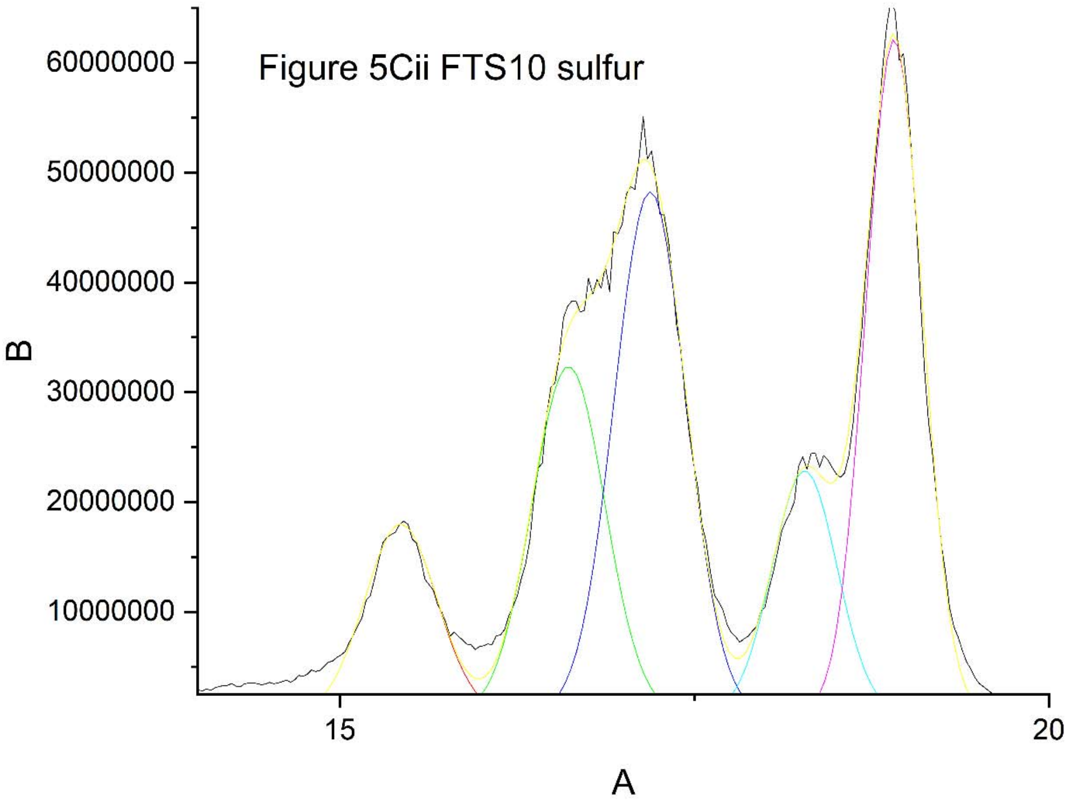
Figure 5Cii FTS10 sulfur
60000000
50000000
40000000
30000000
20000000
10000000
B
15
20
A

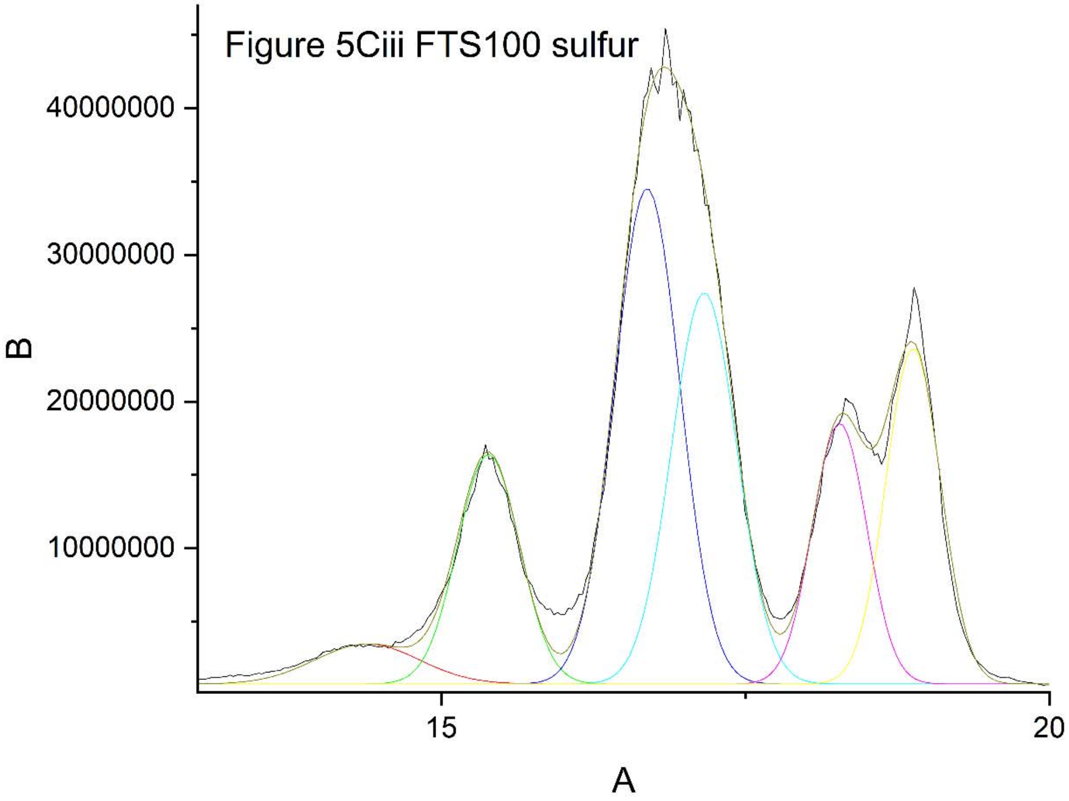
Figure 5Ciii FTS100 sulfur
40000000
30000000
20000000
10000000
B
15
20
A

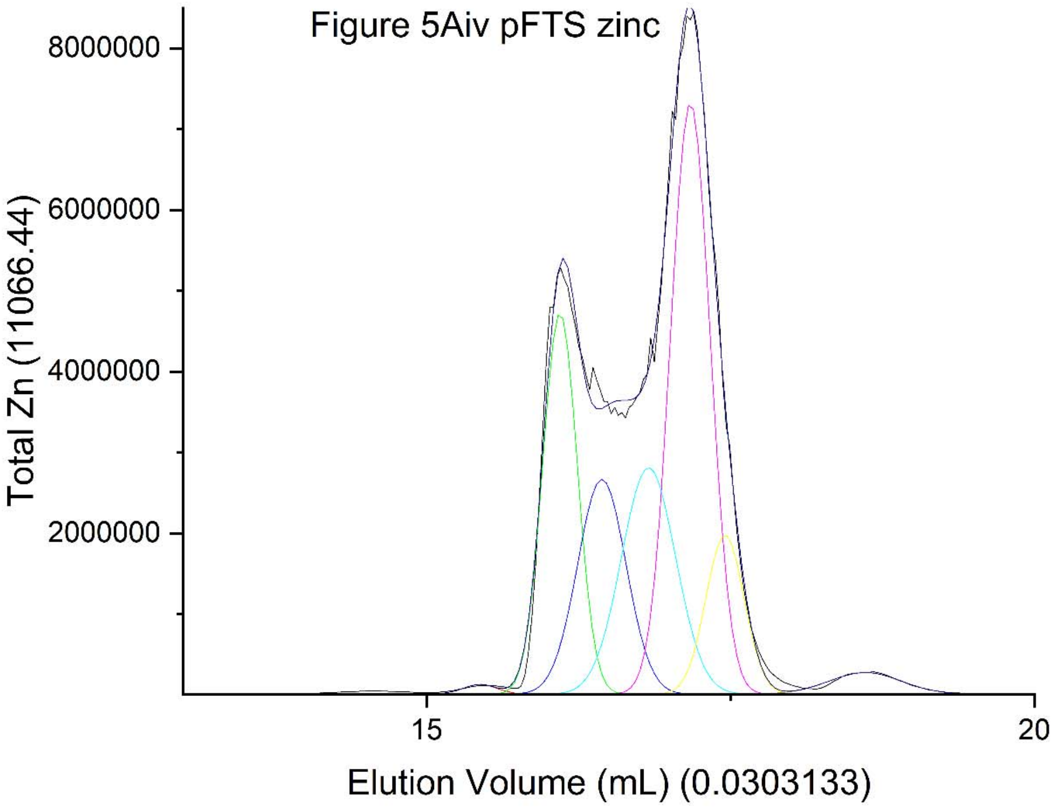

Figure 5Aiv pFTS zinc
8000000
6000000
4000000
2000000
Total Zn (11066.44)
15
20
Elution Volume (mL) (0.0303133)

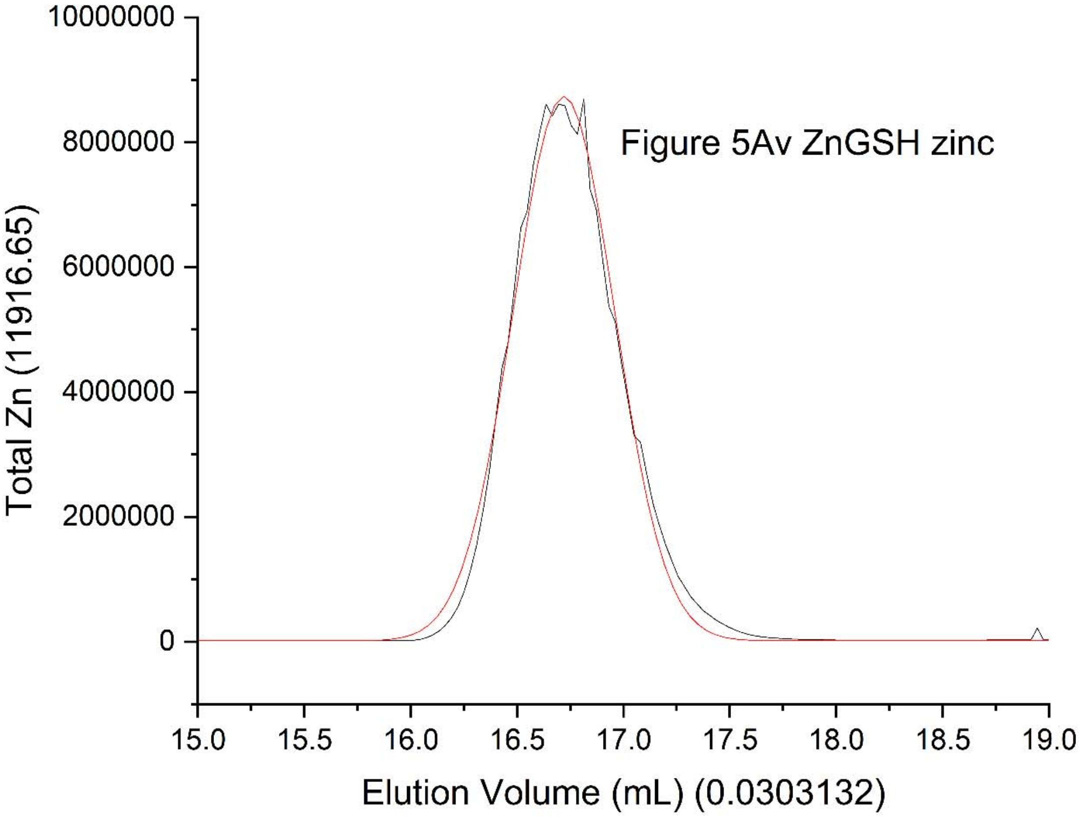
Figure 5Av ZnGSH zinc
Total Zn (11916.65)
10000000
8000000
6000000
4000000
2000000
0
15.0
15.5
16.0
16.5
17.0
17.5
18.0
18.5
19.0
Elution Volume (mL) (0.0303132)

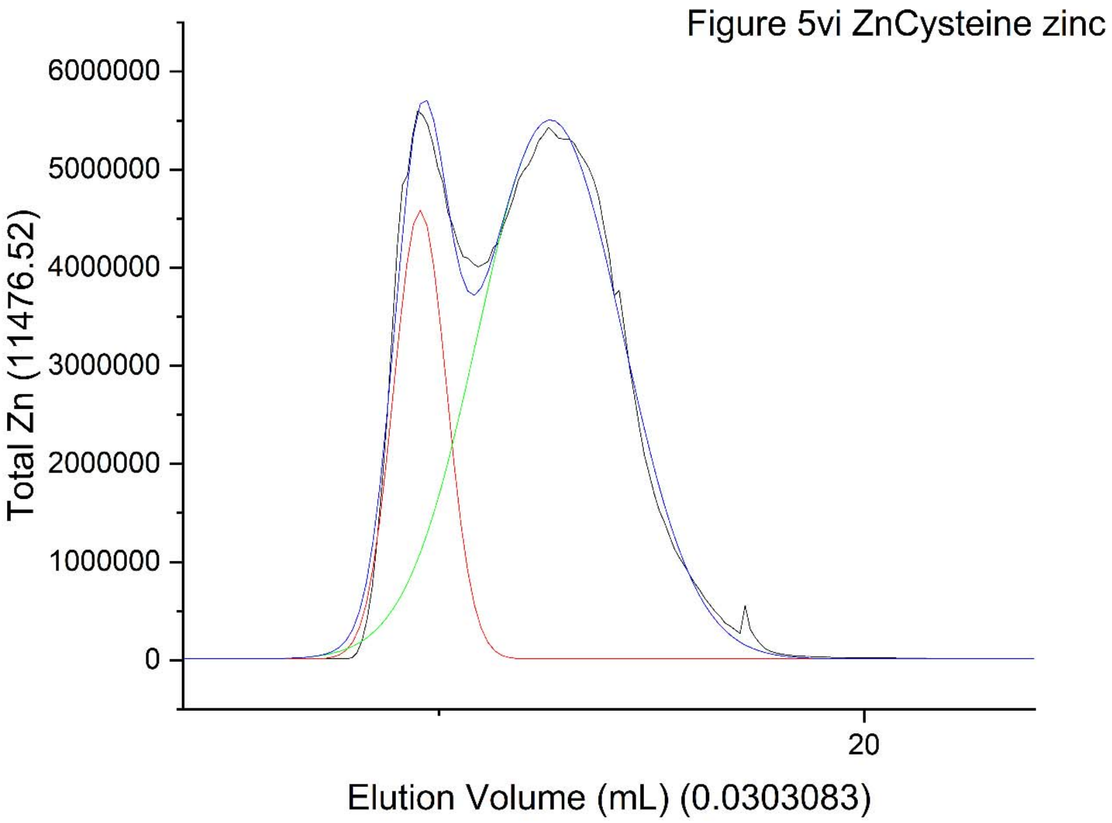
Figure 5vi ZnCysteine zinc
6000000
5000000
4000000
3000000
2000000
1000000
0
Total Zn (11476.52)
20
Elution Volume (mL) (0.0303083)

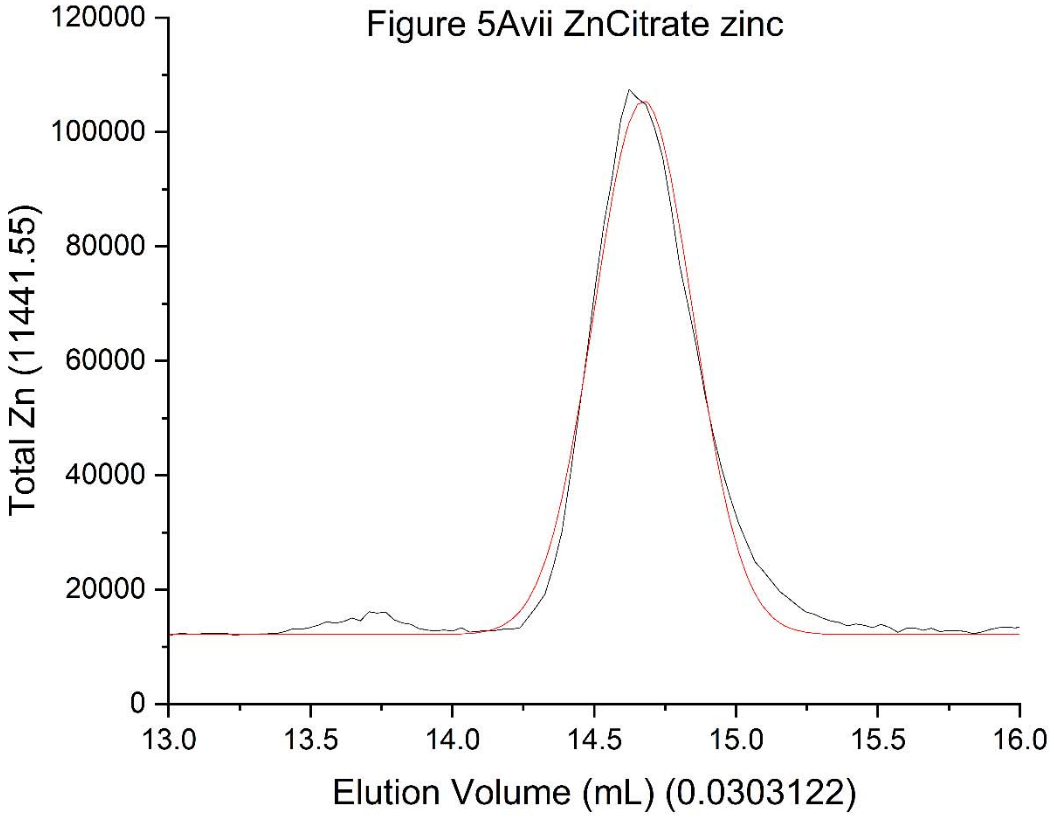
Figure 5Avii ZnCitrate zinc
Total Zn (11441.55)
120000
100000
80000
60000
40000
20000
0
13.0
13.5
14.0
14.5
15.0
15.5
16.0
Elution Volume (mL) (0.0303122)

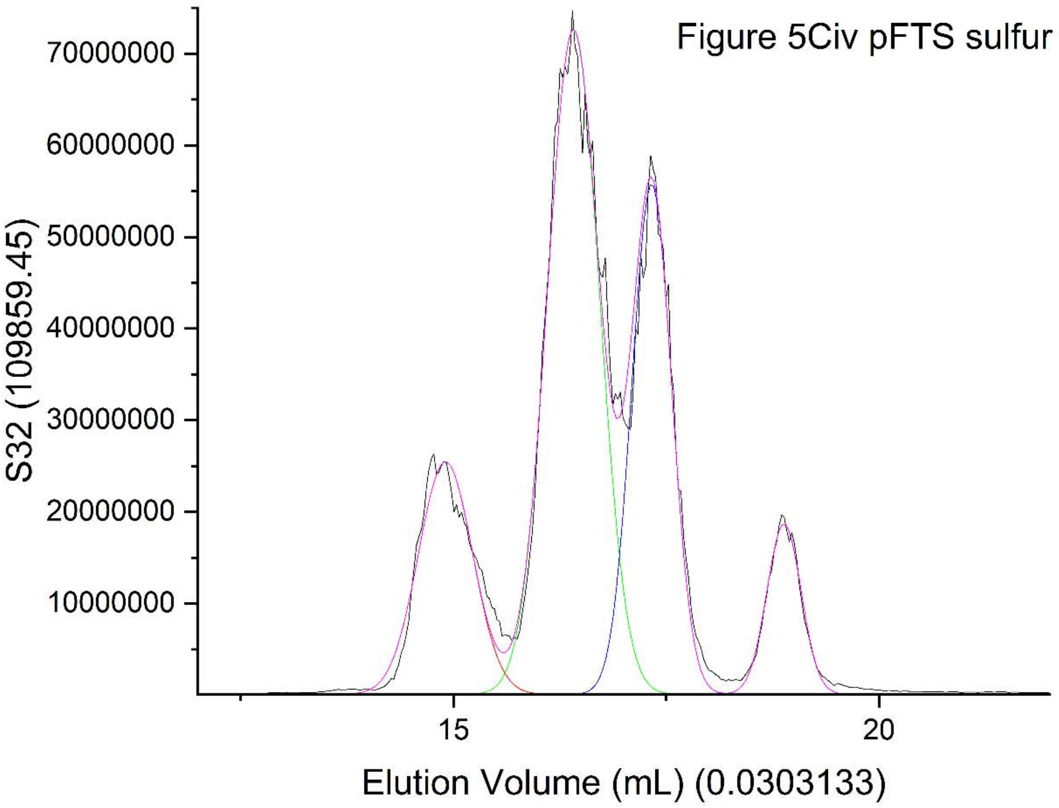
Figure 5Civ pFTS sulfur
70000000
60000000
50000000
40000000
30000000
20000000
10000000
S32 (109859.45)
15
20
Elution Volume (mL) (0.0303133)

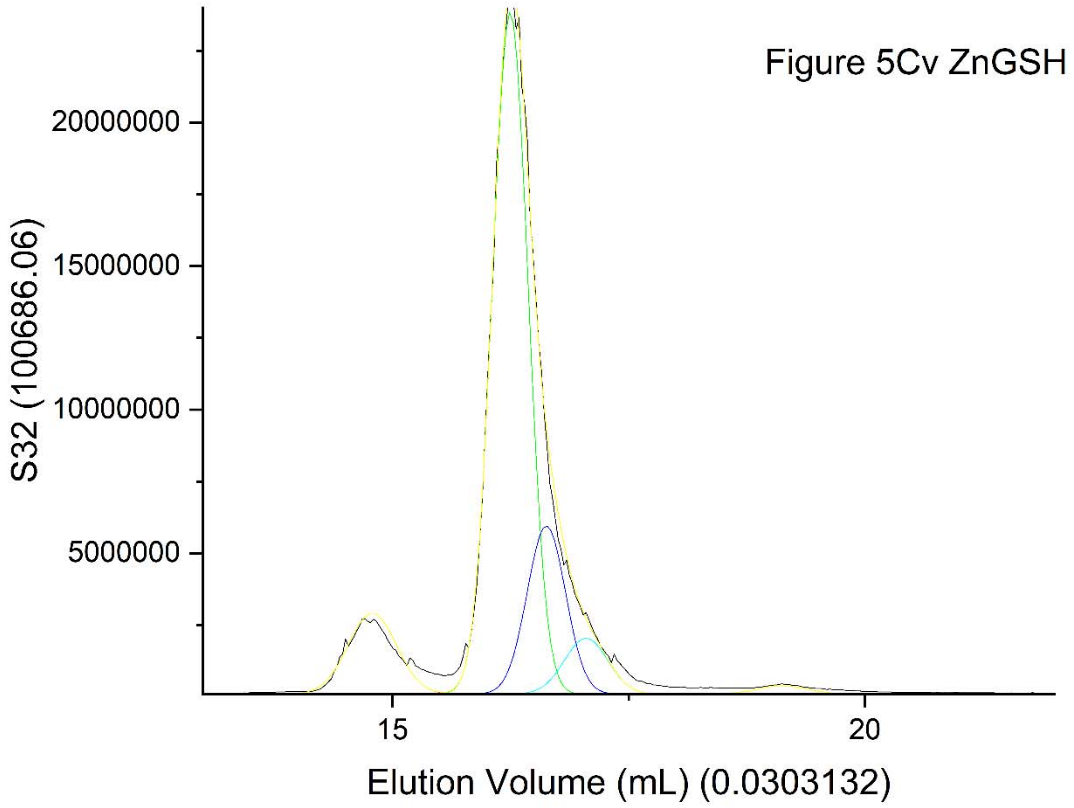

Figure 5Cv ZnGSH
S32 (100686.06)
20000000
15000000
10000000
5000000
15
20
Elution Volume (mL) (0.0303132)

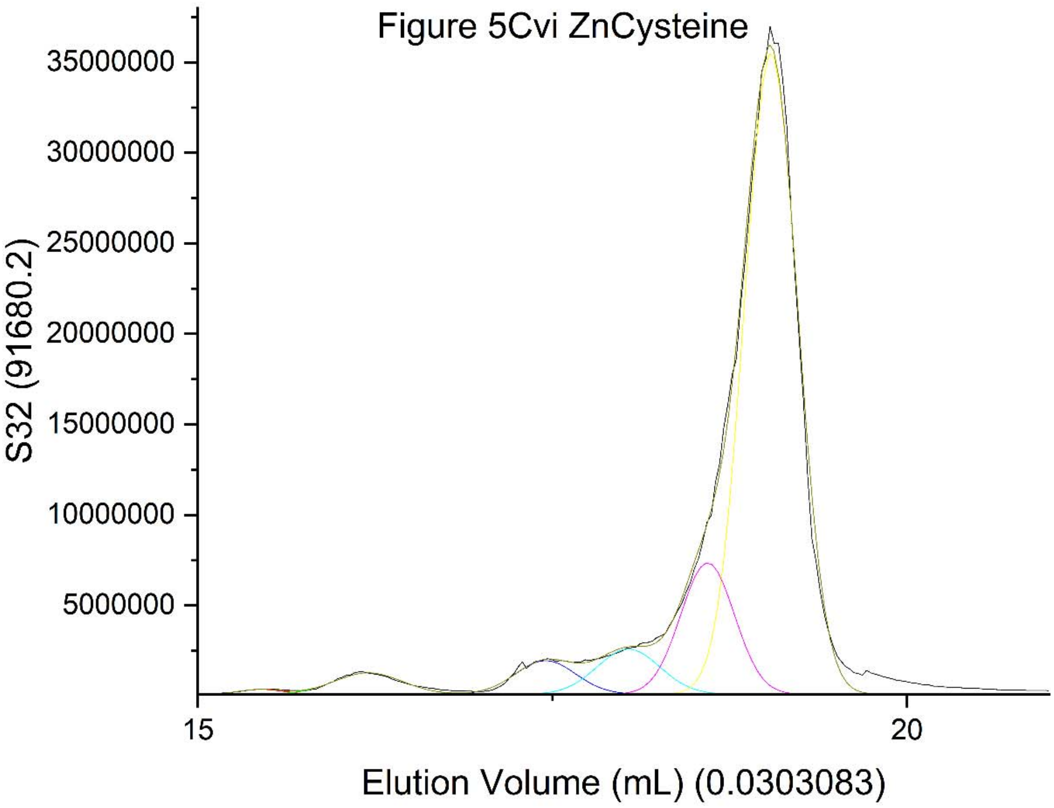

Figure 5Cvi ZnCysteine
35000000
30000000
25000000
20000000
15000000
10000000
5000000
S32 (91680.2)
15
20
Elution Volume (mL) (0.0303083)

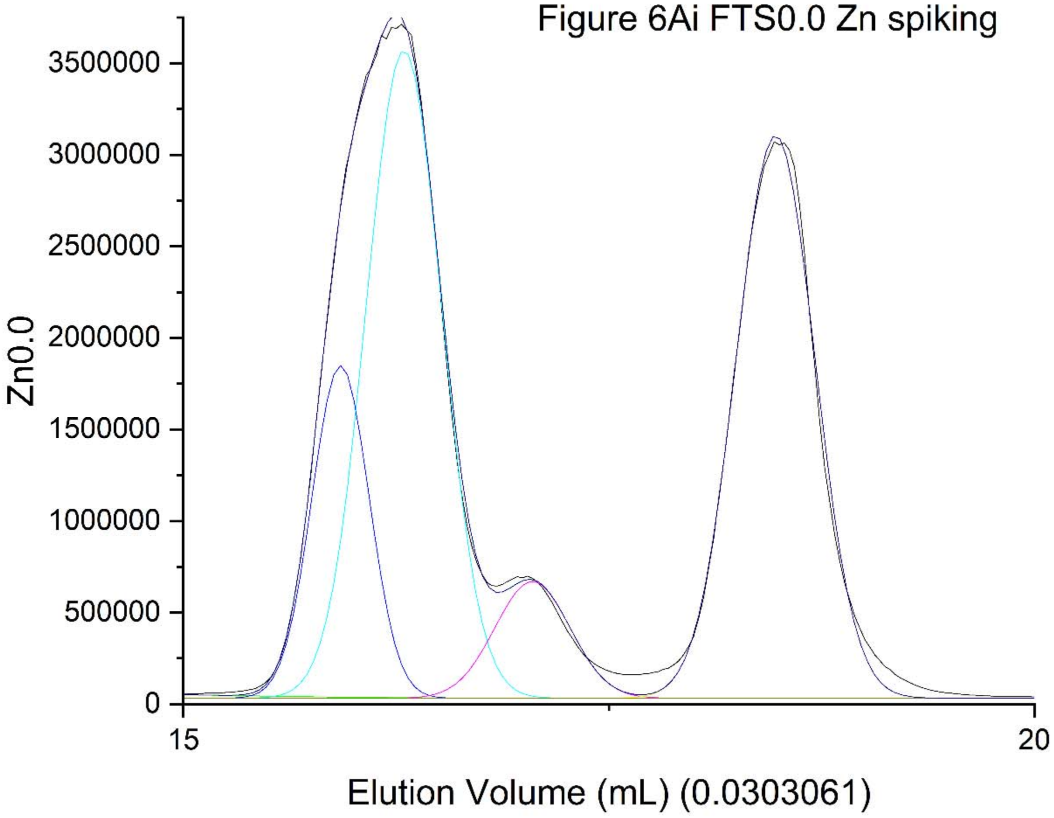

Figure 6Ai FTS0.0 Zn spiking
3500000
3000000
2500000
2000000
1500000
1000000
500000
0
Zn0.0
15
20
Elution Volume (mL) (0.0303061)

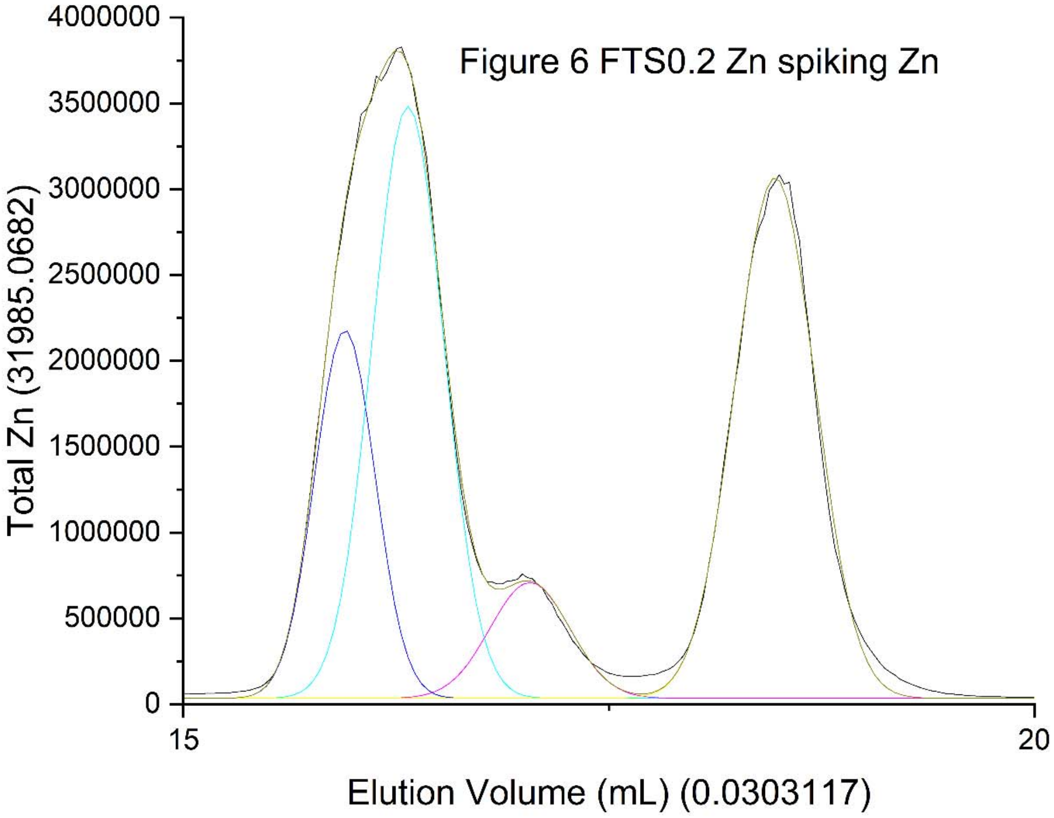

Figure 6 FTS0.2 Zn spiking Zn
Total Zn (31985.0682)
Elution Volume (mL) (0.0303117)
4000000
3500000
3000000
2500000
2000000
1500000
1000000
500000
0
15
20

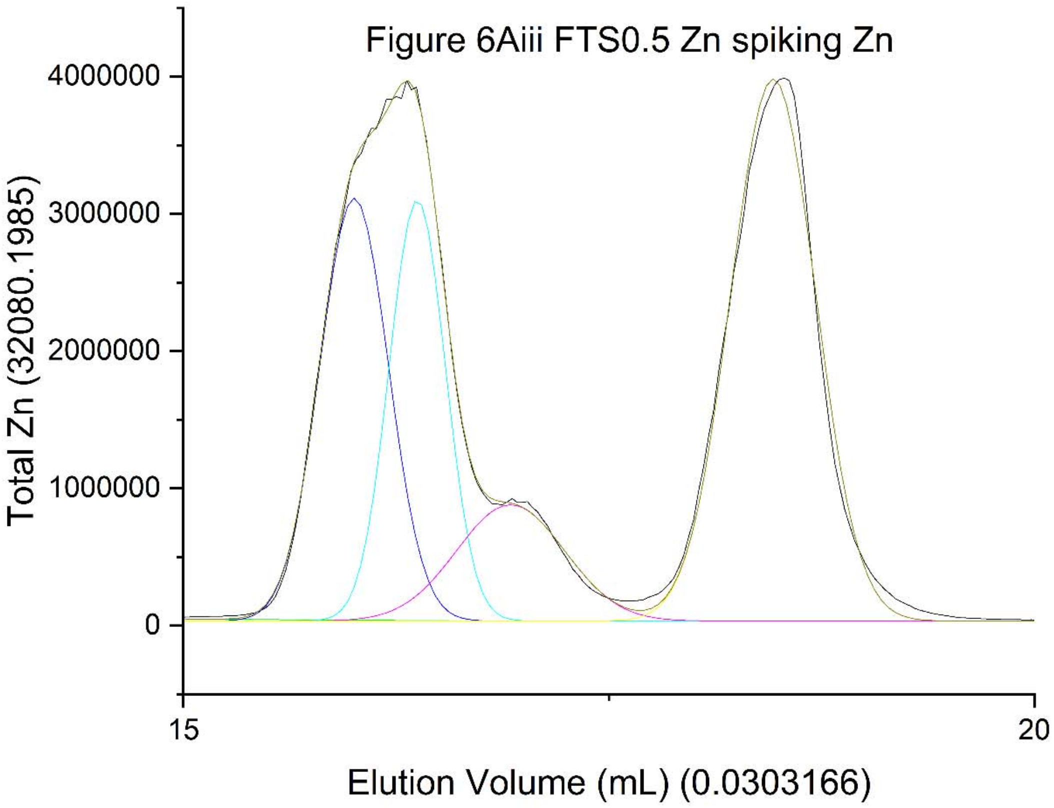
Figure 6Aiii FTS0.5 Zn spiking Zn
4000000
3000000
2000000
1000000
0
Total Zn (32080.1985)
15
20
Elution Volume (mL) (0.0303166)

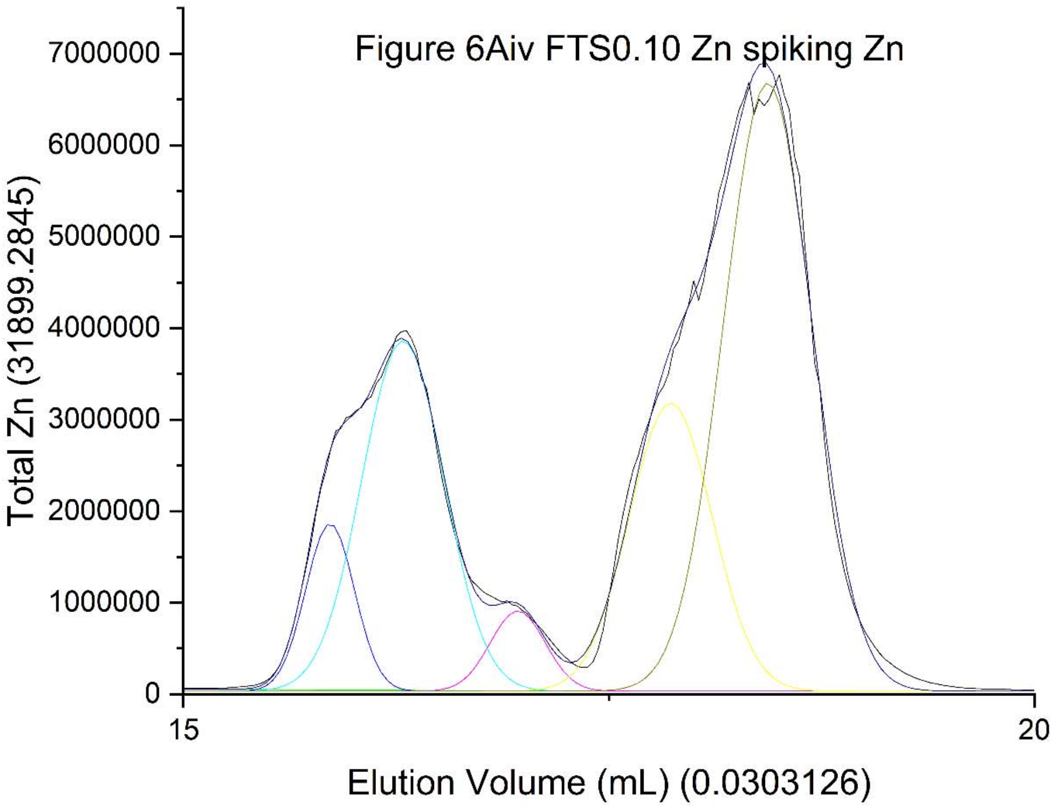
Figure 6Aiv FTS0.10 Zn spiking Zn
Total Zn (31899.2845)
7000000
6000000
5000000
4000000
3000000
2000000
1000000
0
15
20
Elution Volume (mL) (0.0303126)

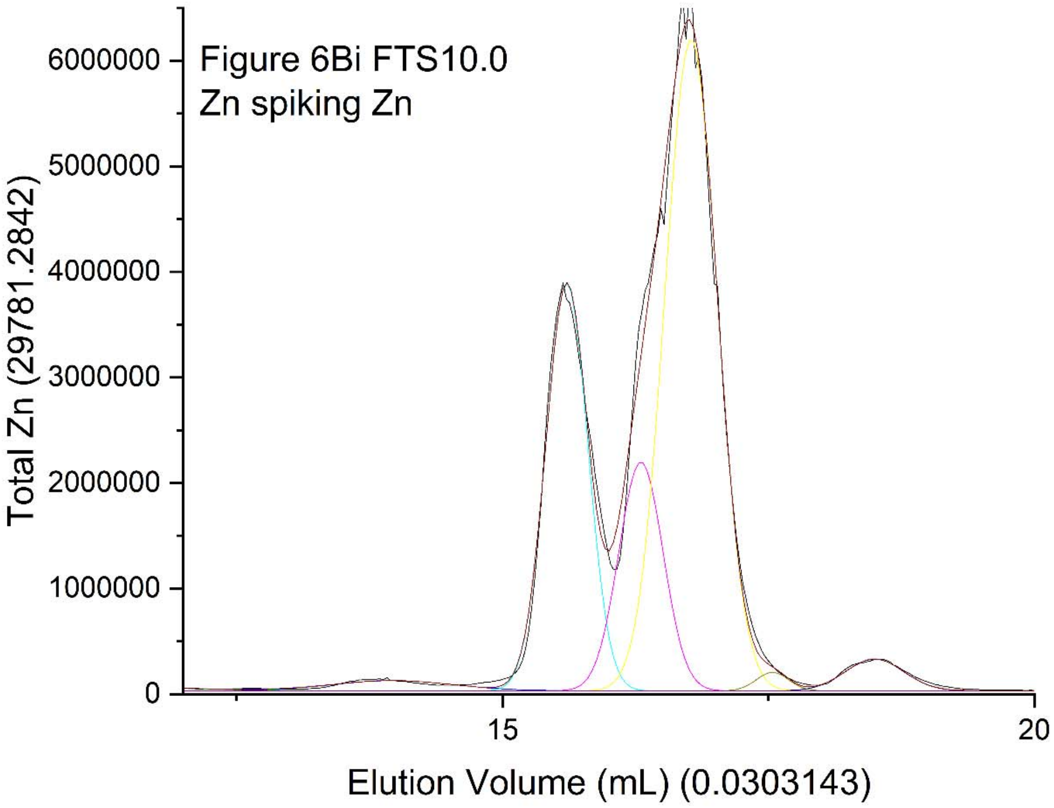
Figure 6Bi FTS10.0
Zn spiking Zn
Total Zn (29781.2842)
6000000
5000000
4000000
3000000
2000000
1000000
0
15
20
Elution Volume (mL) (0.0303143)

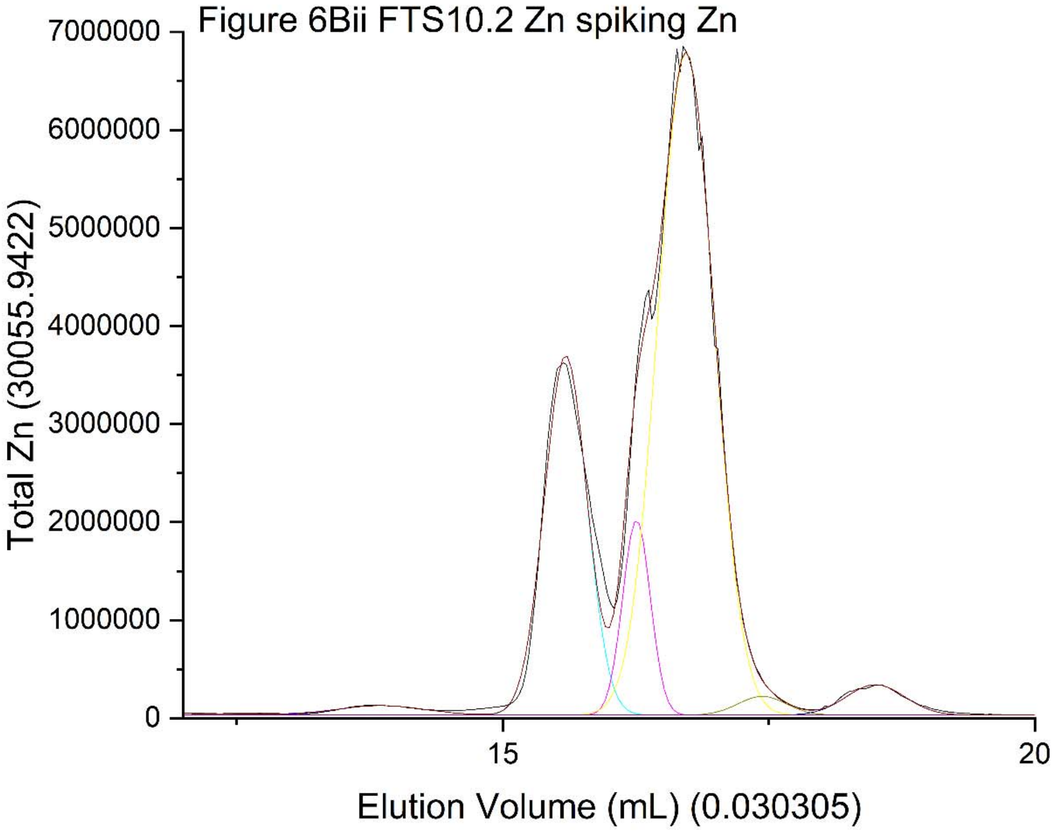
Figure 6Bii FTS10.2 Zn spiking Zn
7000000
6000000
5000000
4000000
3000000
2000000
1000000
0
Total Zn (30055.9422)
15
20
Elution Volume (mL) (0.030305)

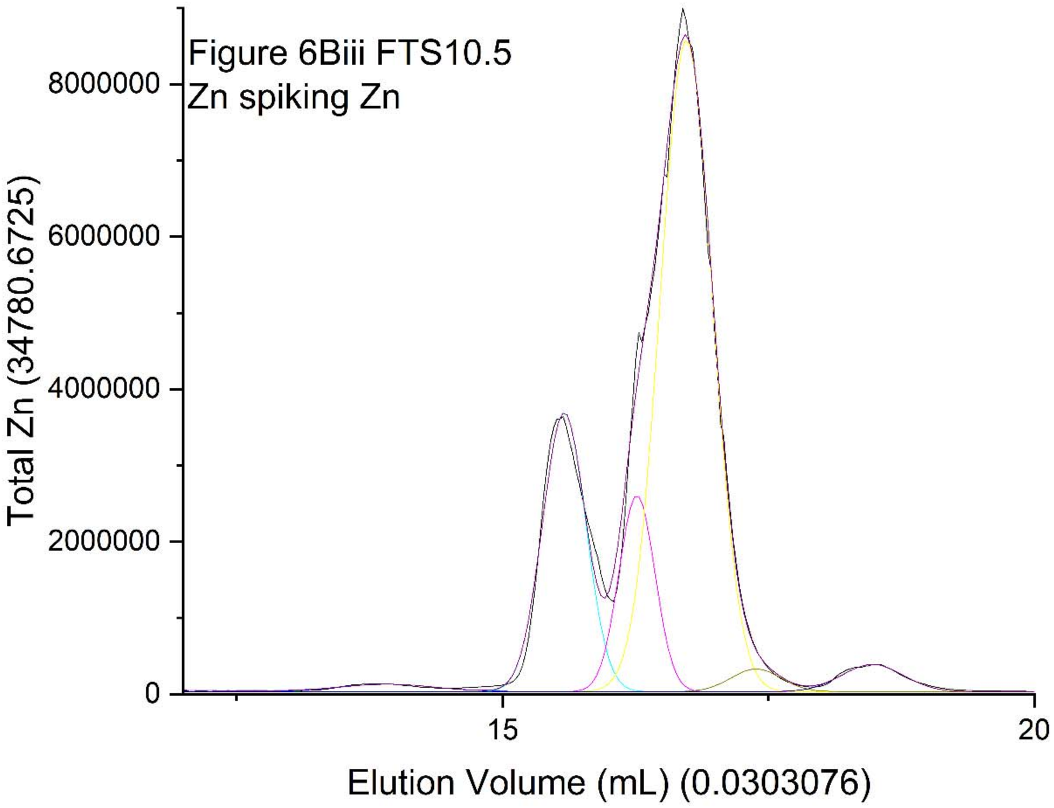
Figure 6Biii FTS10.5
Zn spiking Zn
Total Zn (34780.6725)
8000000
6000000
4000000
2000000
0
15
20
Elution Volume (mL) (0.0303076)

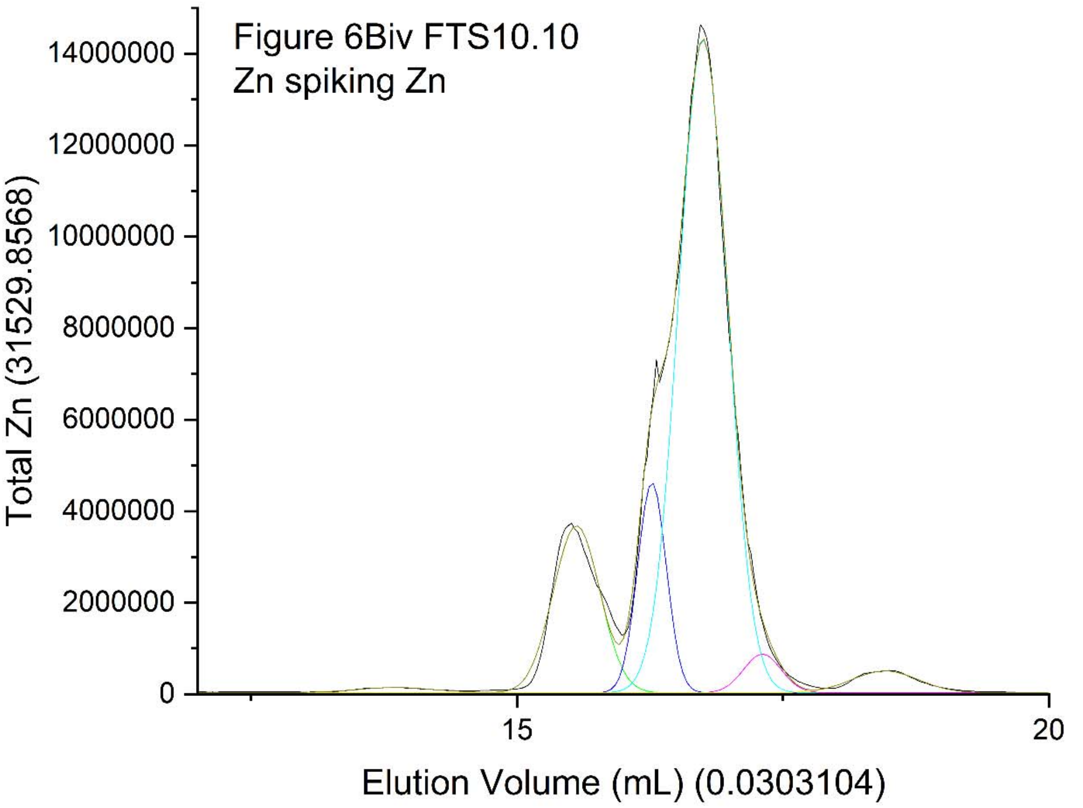

Figure 6Biv FTS10.10
Zn spiking Zn
Total Zn (31529.8568)
14000000
12000000
10000000
8000000
6000000
4000000
2000000
0
15
20
Elution Volume (mL) (0.0303104)

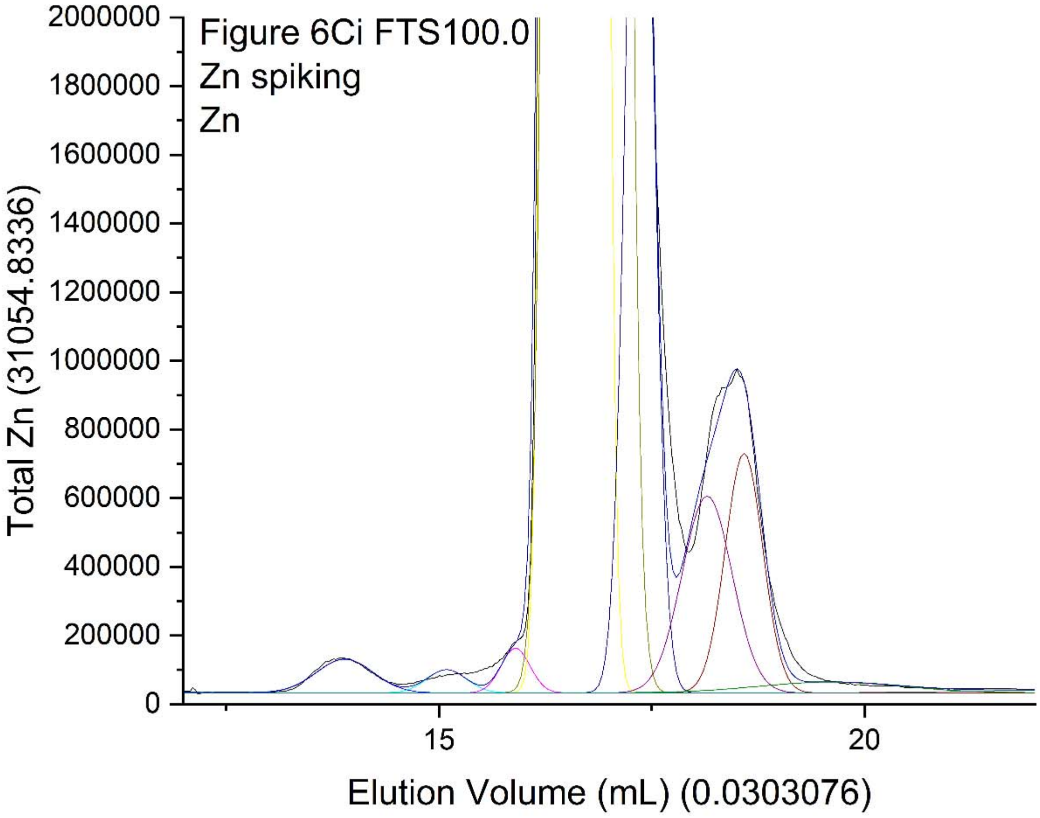

Figure 6Ci FTS100.0
Zn spiking
Zn
Total Zn (31054.8336)
2000000
1800000
1600000
1400000
1200000
1000000
800000
600000
400000
200000
0
15
20
Elution Volume (mL) (0.0303076)

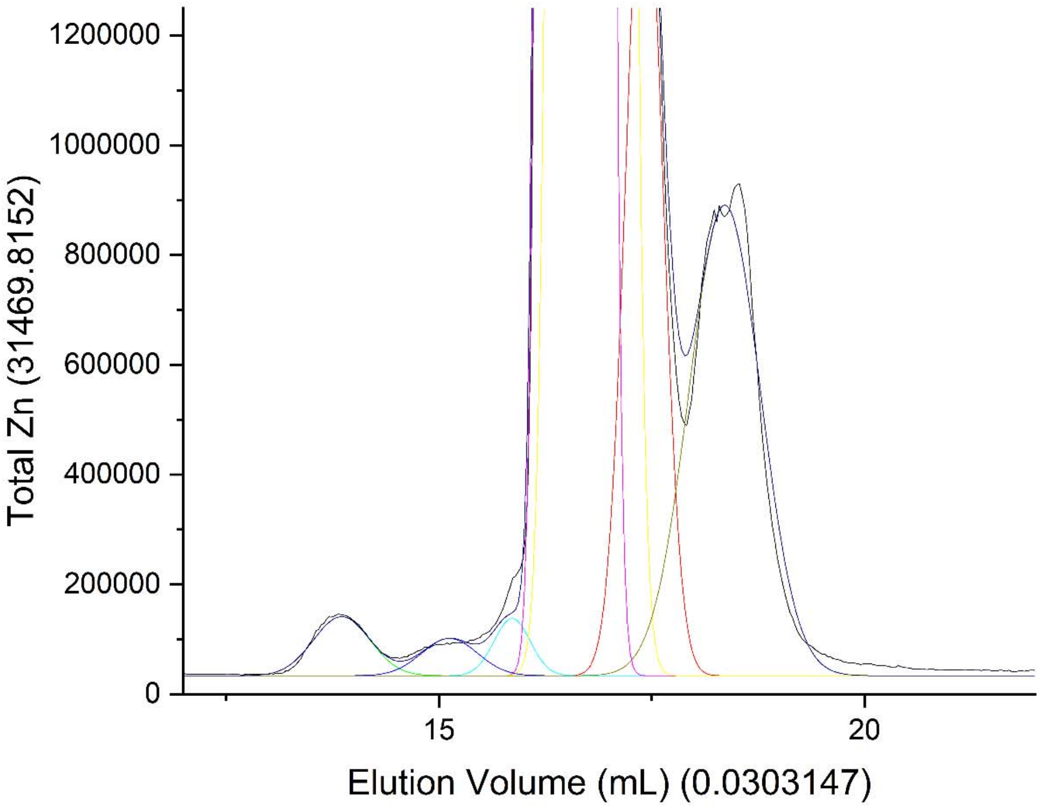
1200000
1000000
800000
600000
400000
200000
0
Total Zn (31469.8152)
15
20
Elution Volume (mL) (0.0303147)

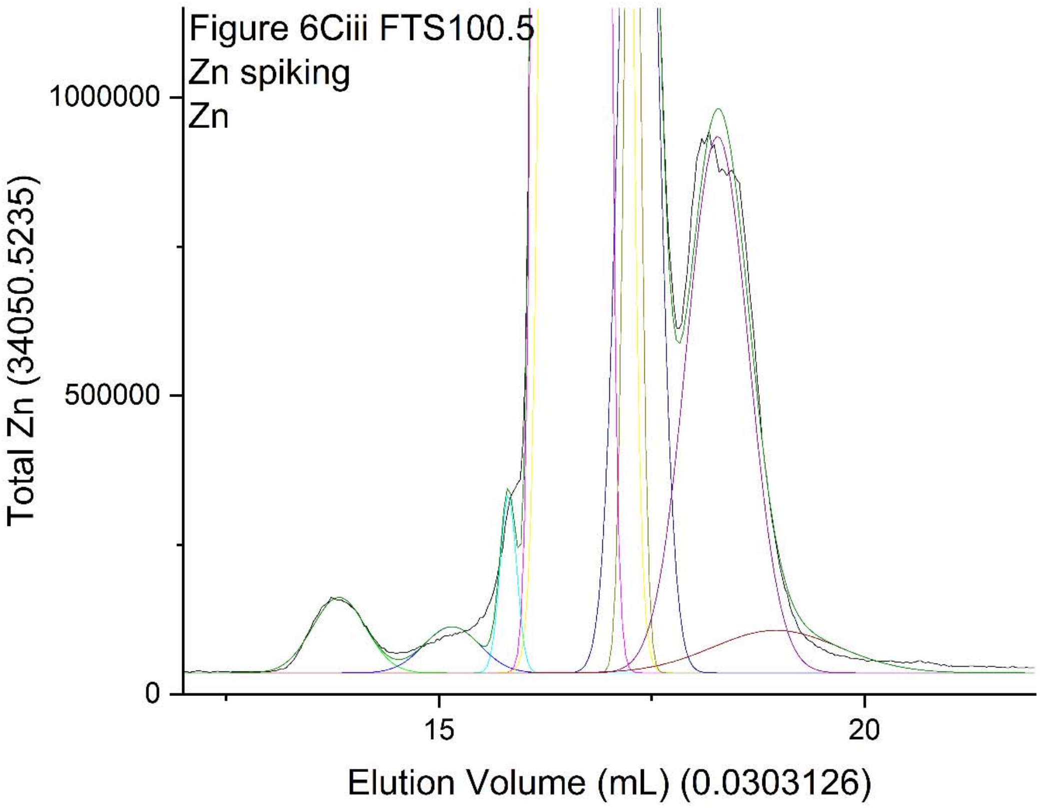
Figure 6Ciii FTS100.5
Zn spiking
Zn
1000000
500000
0
Total Zn (34050.5235)
15
20
Elution Volume (mL) (0.0303126)

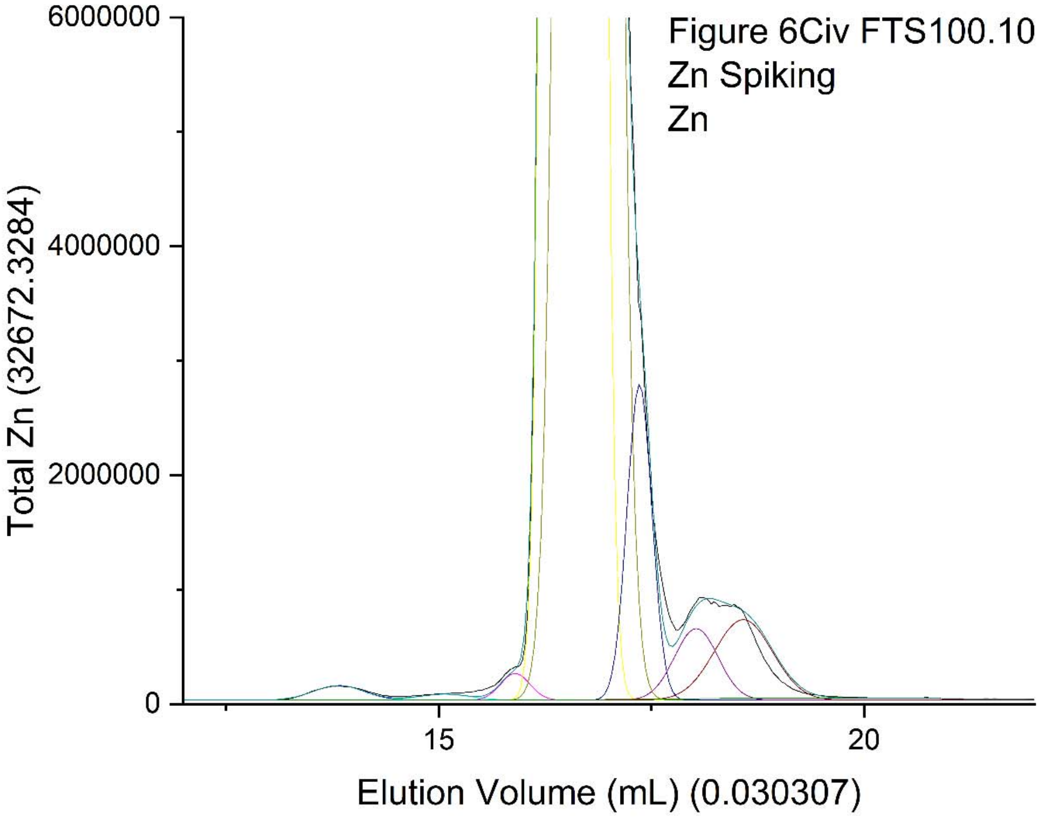
Figure 6Civ FTS100.10
Zn Spiking
Zn
Total Zn (32672.3284)
6000000
4000000
2000000
0
15
20
Elution Volume (mL) (0.030307)

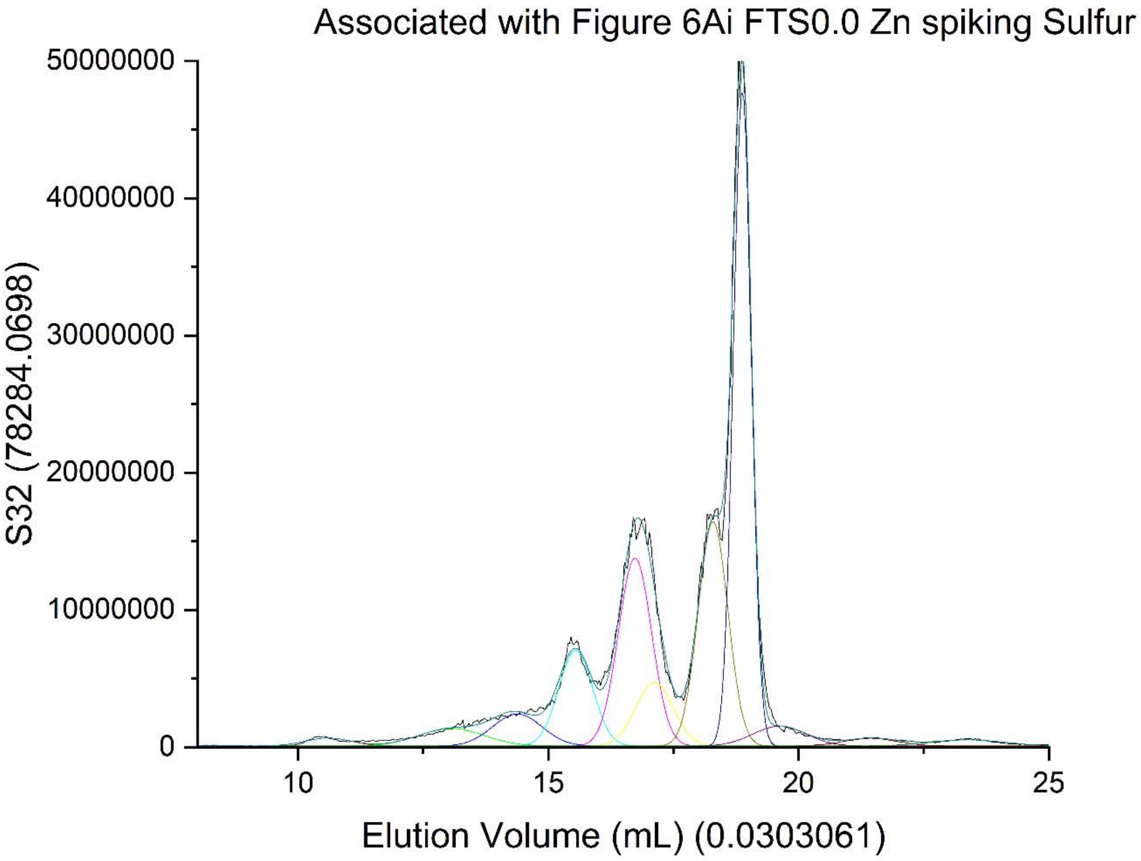
Associated with Figure 6Ai FTS0.0 Zn spiking Sulfur
S32 (78284.0698)
50000000
40000000
30000000
20000000
10000000
0
10
15
20
25
Elution Volume (mL) (0.0303061)

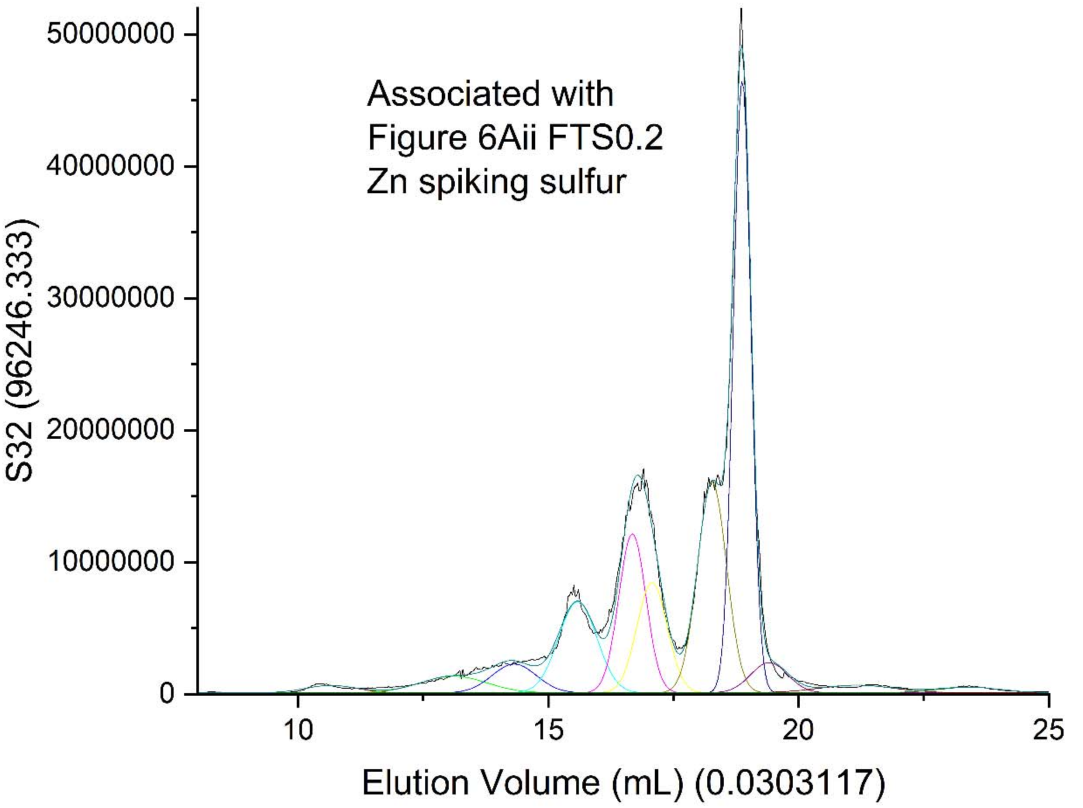
Associated with
Figure 6Aii FTS0.2
Zn spiking sulfur
S32 (96246.333)
50000000
40000000
30000000
20000000
10000000
0
10
15
20
25
Elution Volume (mL) (0.0303117)

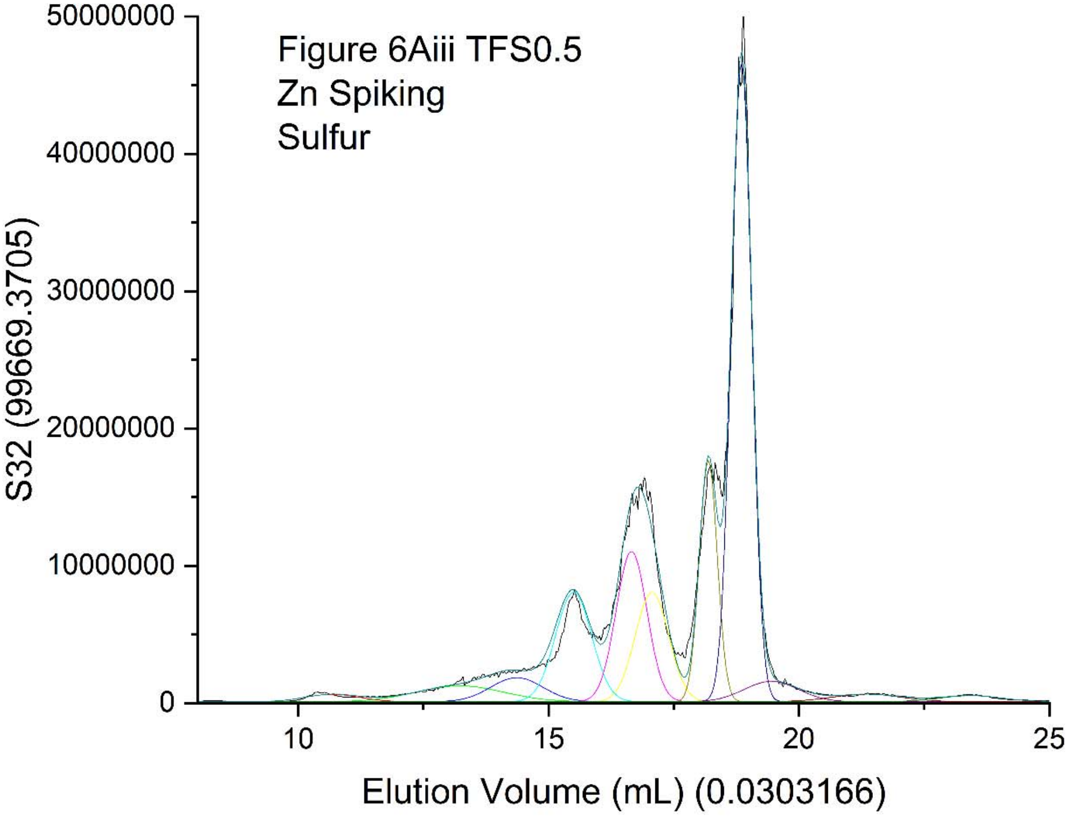

Figure 6Aiii TFS0.5
Zn Spiking
Sulfur
S32 (99669.3705)
50000000
40000000
30000000
20000000
10000000
0
10
15
20
25
Elution Volume (mL) (0.0303166)

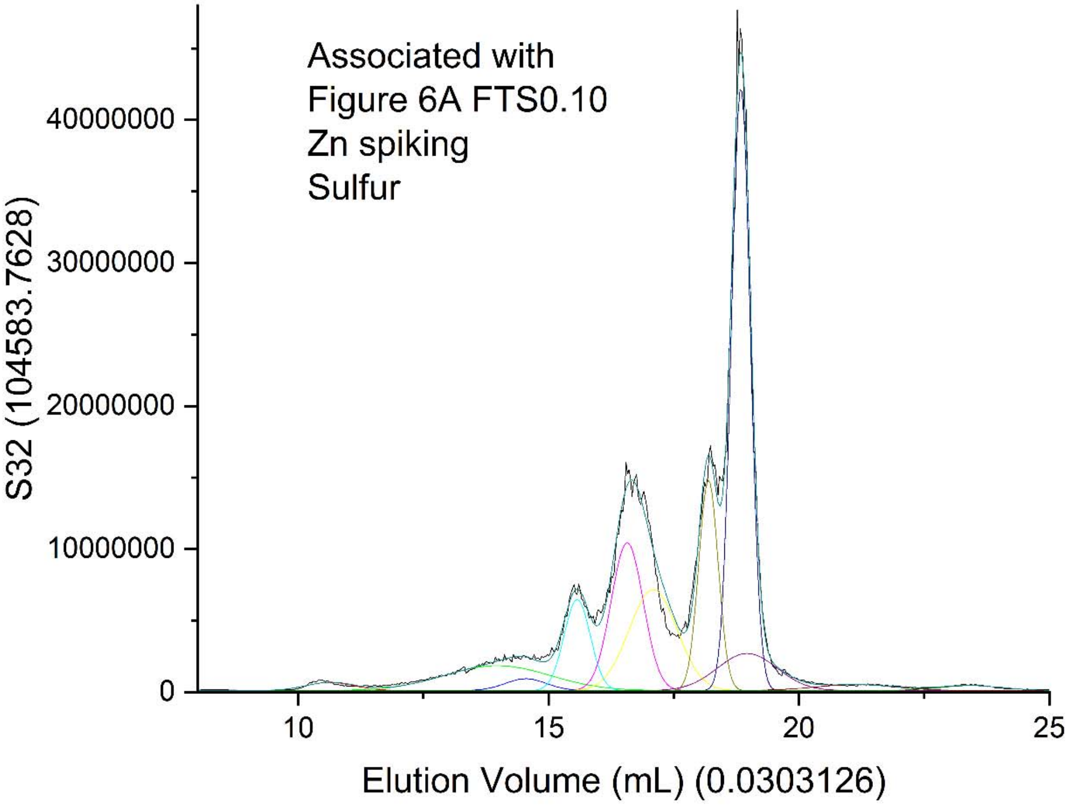
Associated with
Figure 6A FTS0.10
Zn spiking
Sulfur
S32 (104583.7628)
40000000
30000000
20000000
10000000
0
10
15
20
25
Elution Volume (mL) (0.0303126)

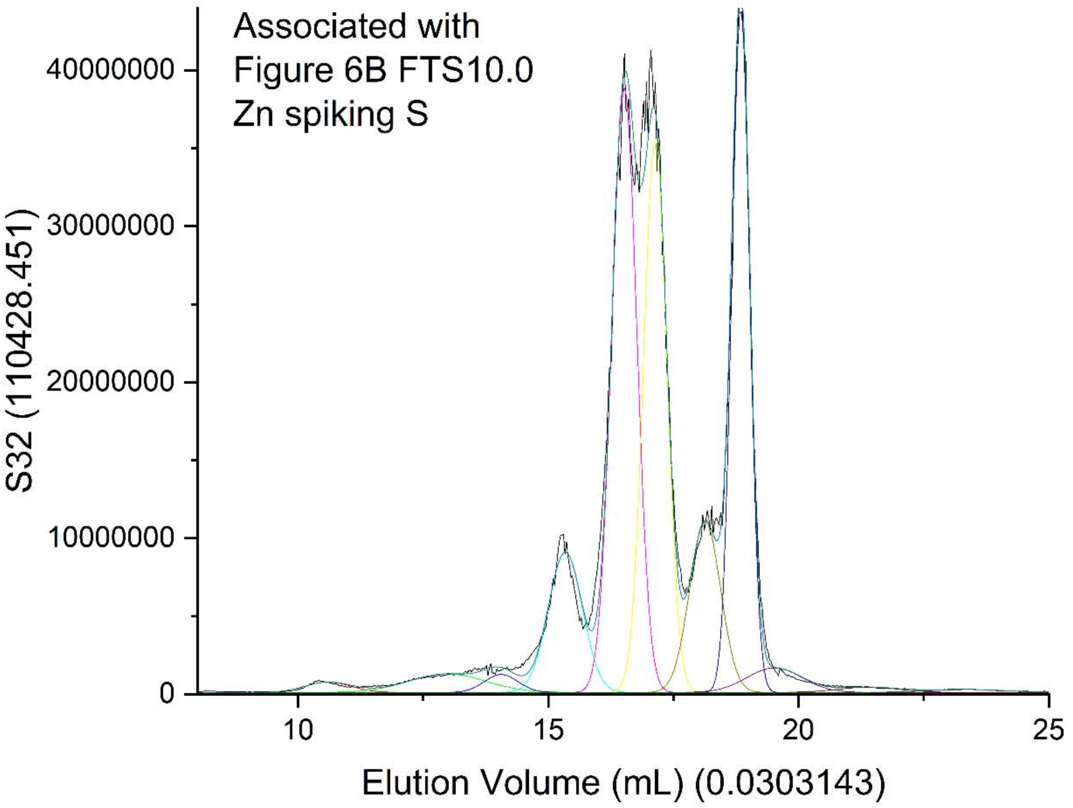
Associated with
Figure 6B FTS10.0
Zn spiking S
40000000
30000000
20000000
10000000
0
S32 (110428.451)
10
15
20
25
Elution Volume (mL) (0.0303143)

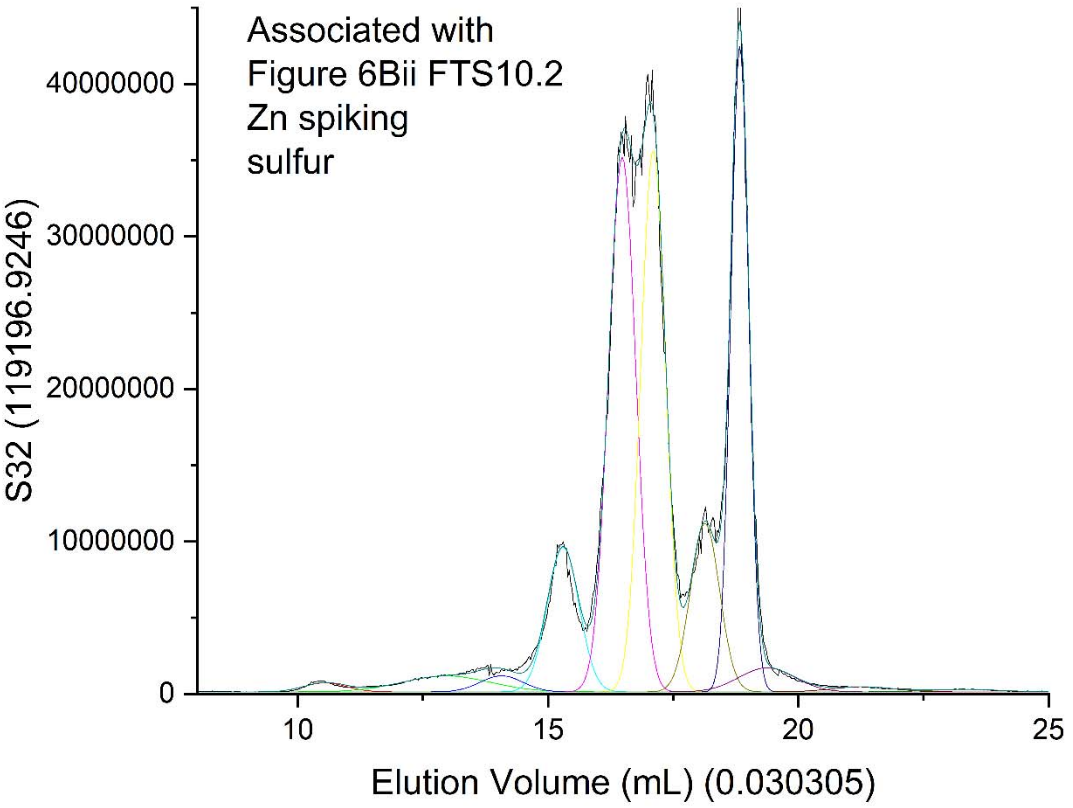
Associated with
Figure 6Bii FTS10.2
Zn spiking
sulfur
S32 (119196.9246)
40000000
30000000
20000000
10000000
0
10
15
20
25
Elution Volume (mL) (0.030305)

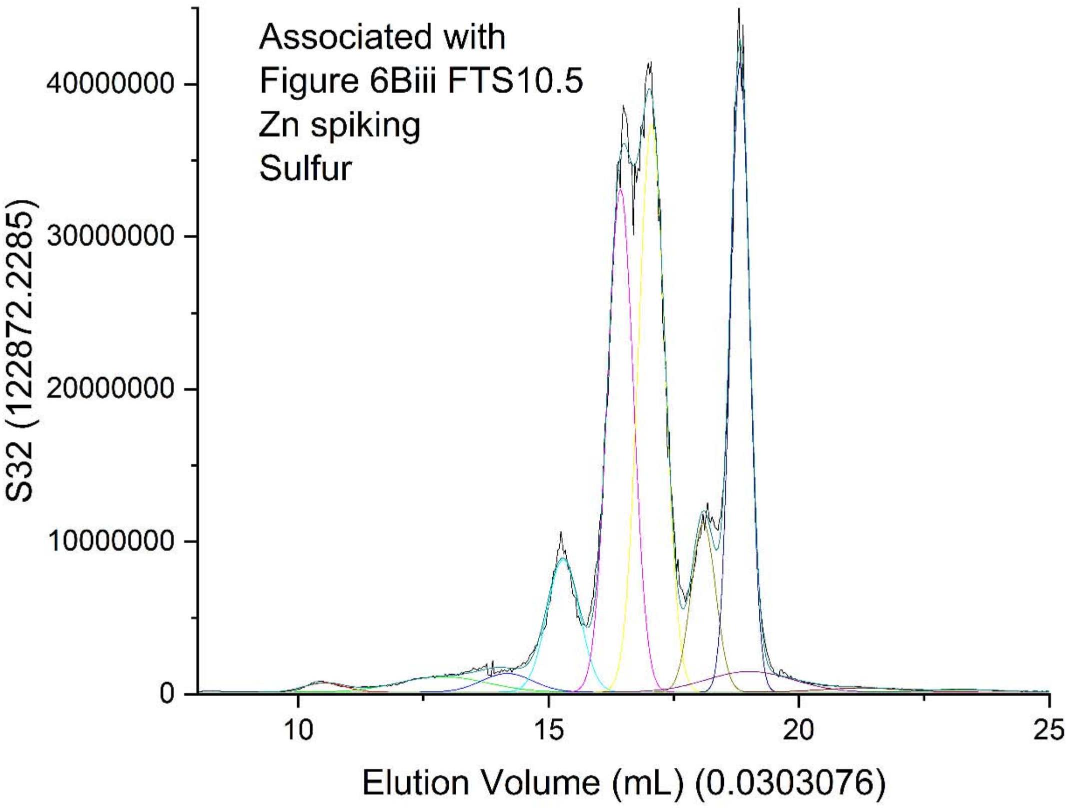
Associated with
Figure 6Biii FTS10.5
Zn spiking
Sulfur
S32 (122872.2285)
40000000
30000000
20000000
10000000
0
10
15
20
25
Elution Volume (mL) (0.0303076)

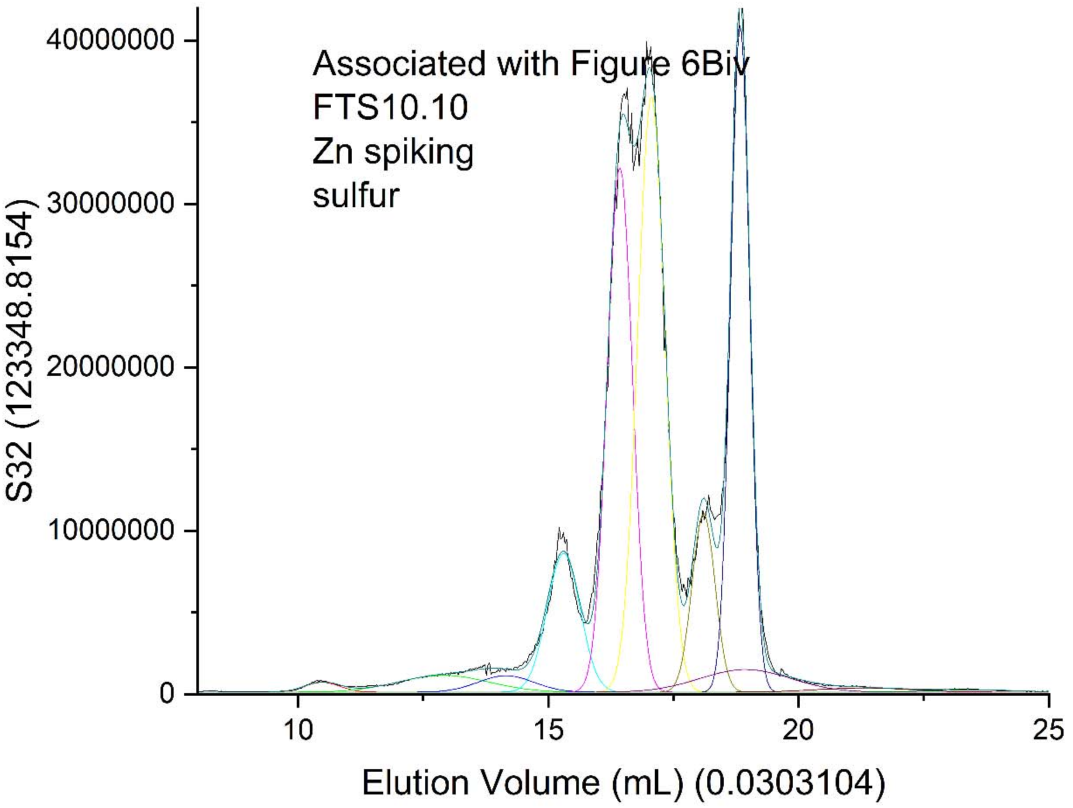
Associated with Figure 6Biv
FTS10.10
Zn spiking
sulfur
S32 (123348.8154)
40000000
30000000
20000000
10000000
0
10
15
20
25
Elution Volume (mL) (0.0303104)

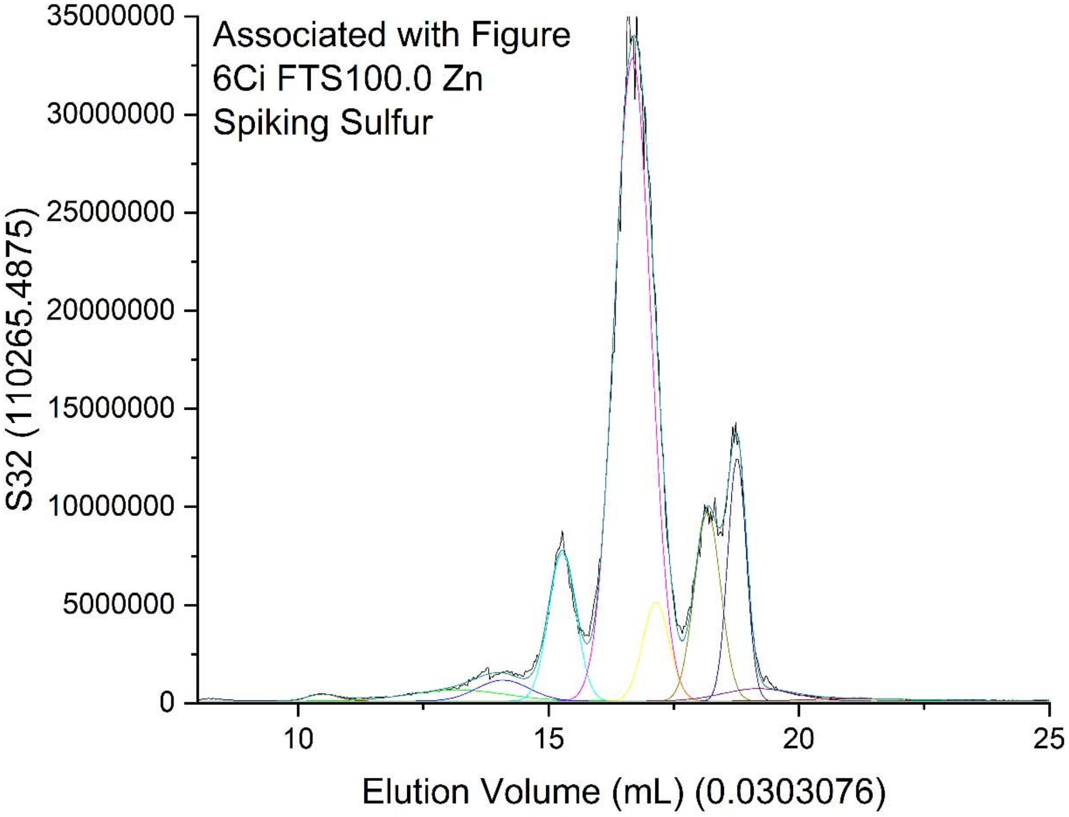
Associated with Figure
6Ci FTS100.0 Zn
Spiking Sulfur
S32 (110265.4875)
35000000
30000000
25000000
20000000
15000000
10000000
5000000
0
10
15
20
25
Elution Volume (mL) (0.0303076)

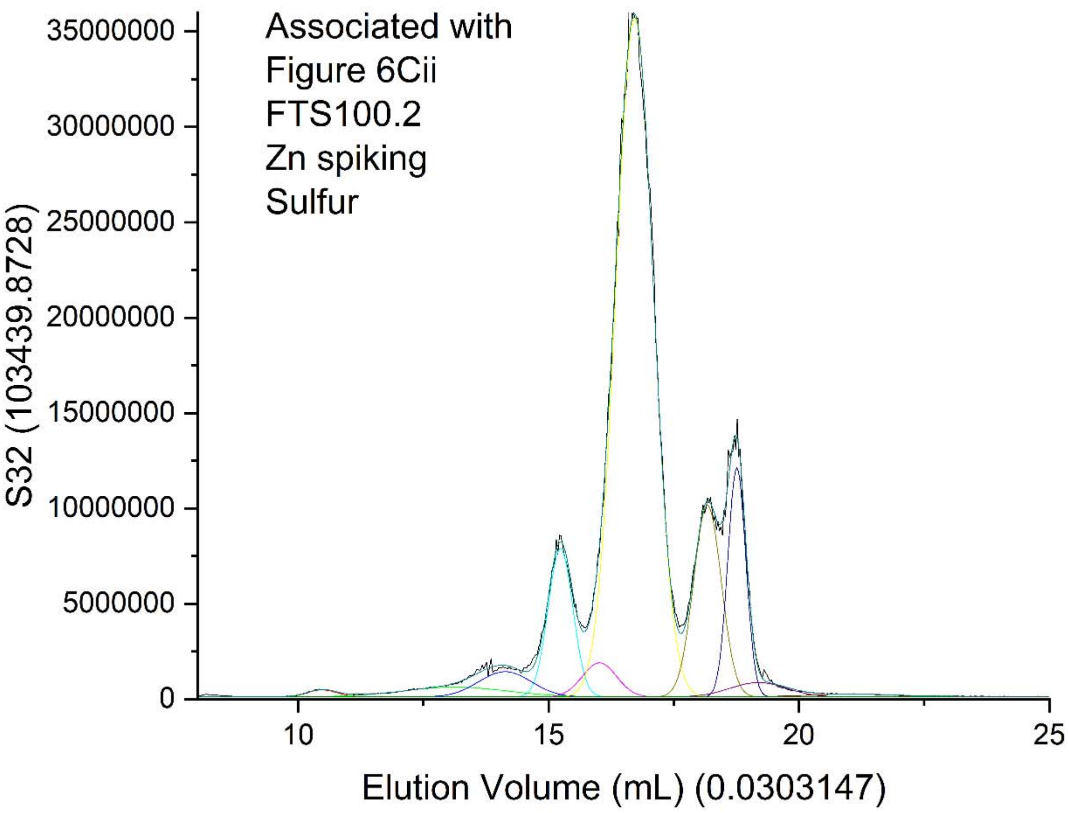
Associated with
Figure 6Cii
FTS100.2
Zn spiking
Sulfur
S32 (103439.8728)
35000000
30000000
25000000
20000000
15000000
10000000
5000000
0
10
15
20
25
Elution Volume (mL) (0.0303147)

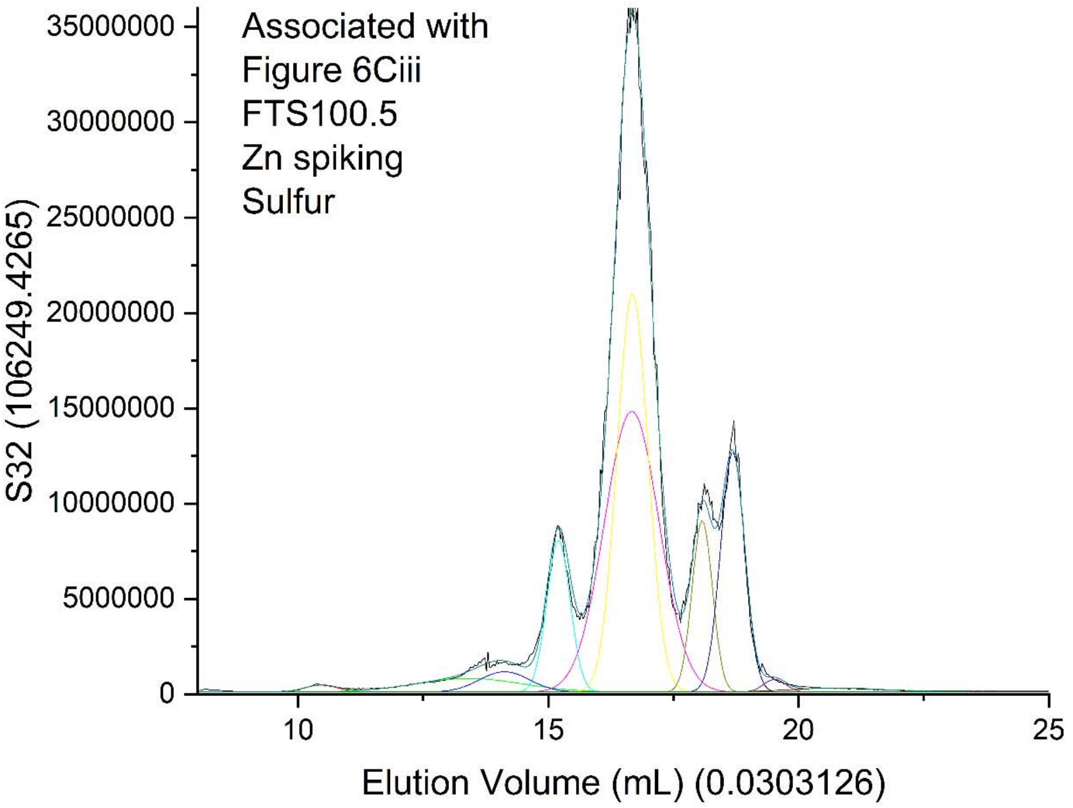
Associated with
Figure 6Ciii
FTS100.5
Zn spiking
Sulfur
S32 (106249.4265)
35000000
30000000
25000000
20000000
15000000
10000000
5000000
0
10
15
20
25
Elution Volume (mL) (0.0303126)

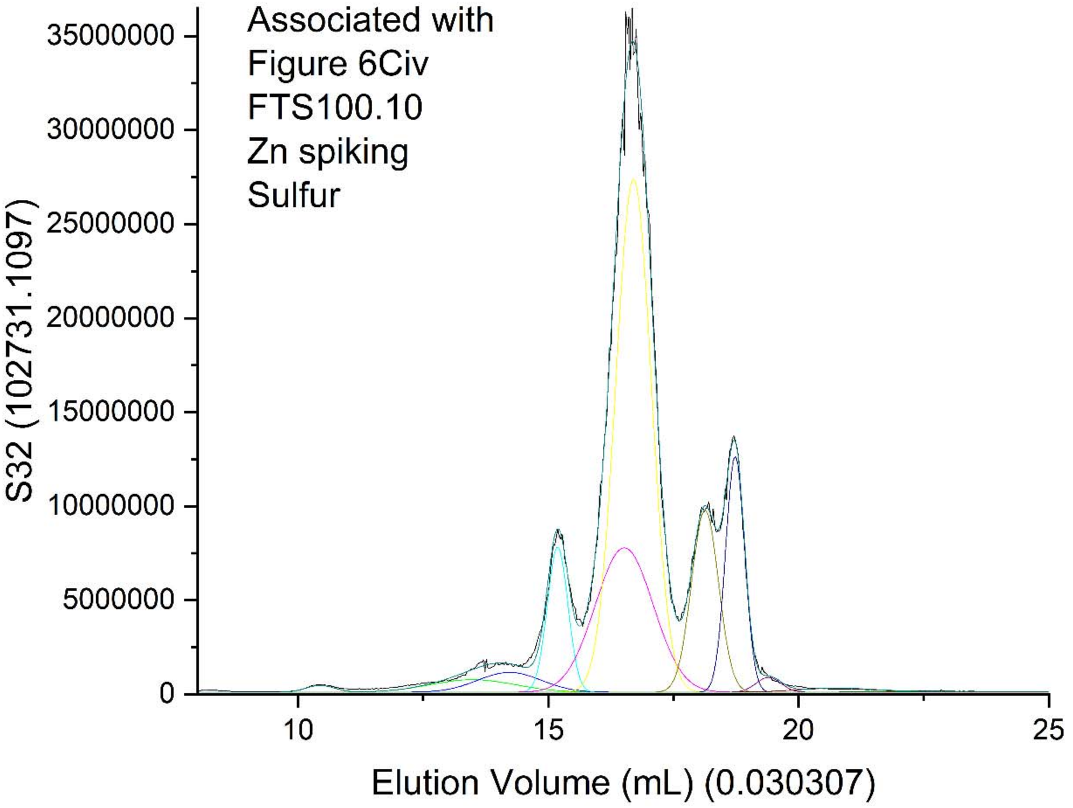
Associated with
Figure 6Civ
FTS100.10
Zn spiking
Sulfur
S32 (102731.1097)
35000000
30000000
25000000
20000000
15000000
10000000
5000000
0
10
15
20
25
Elution Volume (mL) (0.030307)

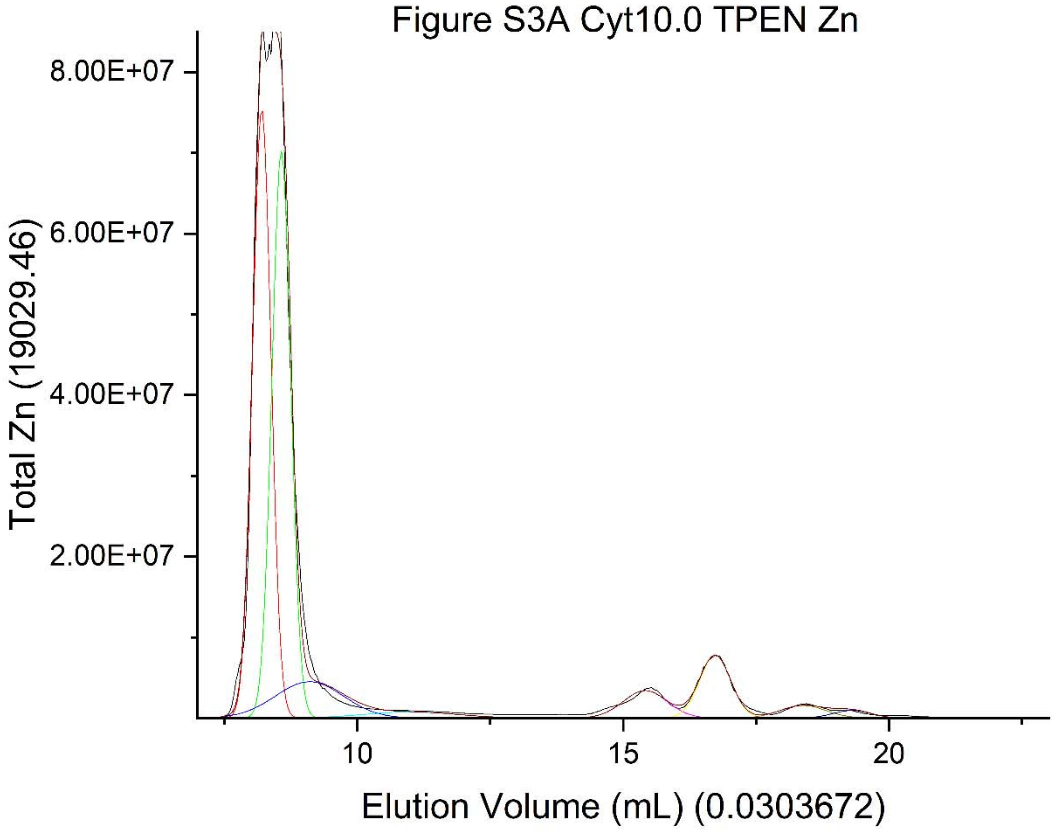

Figure S3A Cyt10.0 TPEN Zn
8.00E+07
6.00E+07
4.00E+07
2.00E+07
Total Zn (19029.46)
10
15
20
Elution Volume (mL) (0.0303672)

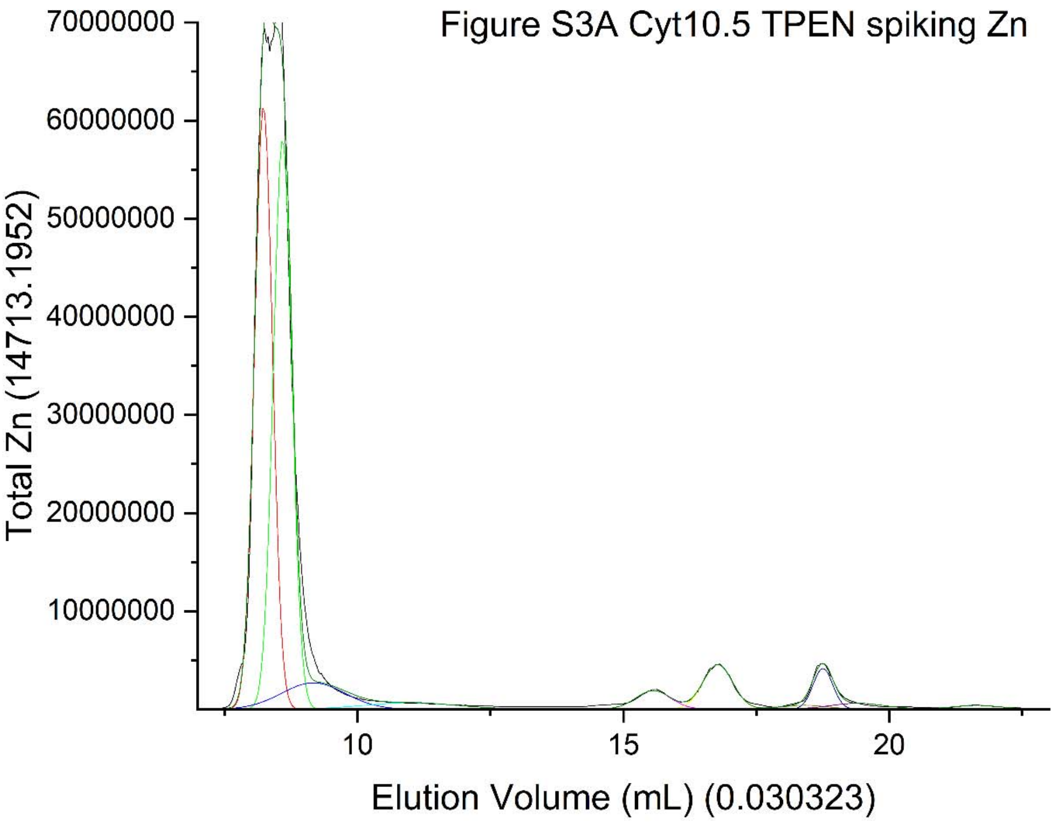
Figure S3A Cyt10.5 TPEN spiking Zn
Total Zn (14713.1952)
70000000
60000000
50000000
40000000
30000000
20000000
10000000
10
15
20
Elution Volume (mL) (0.030323)

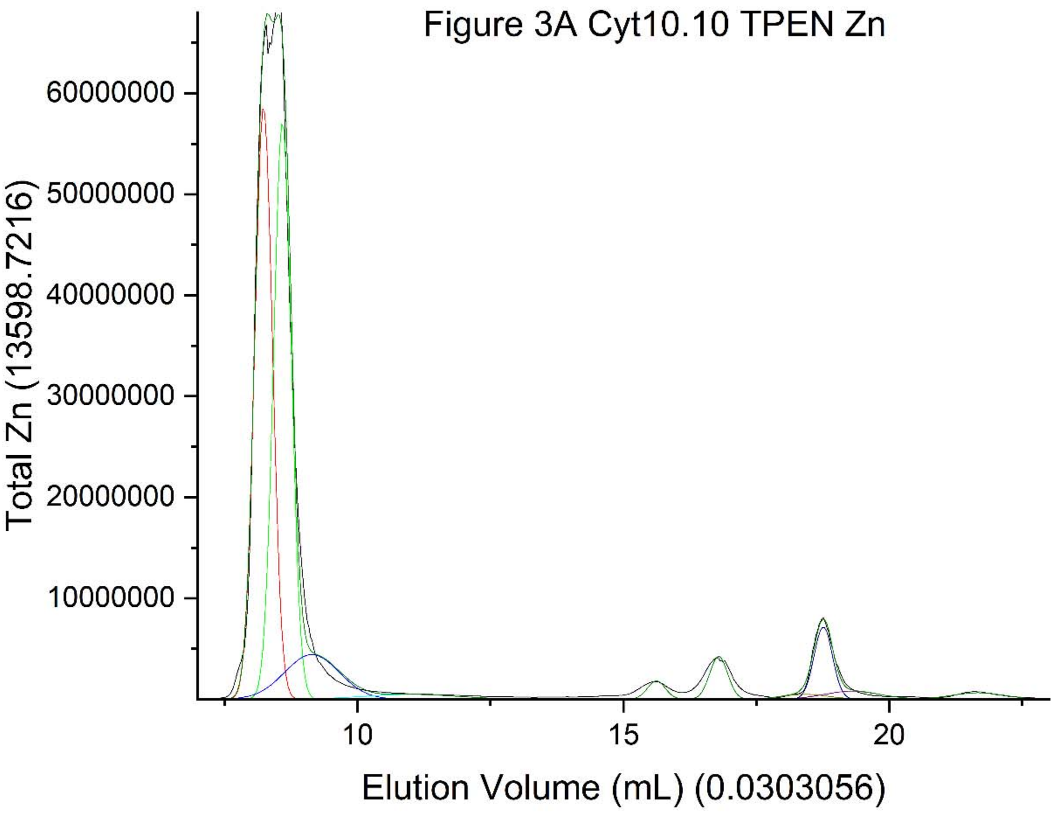

Figure 3A Cyt10.10 TPEN Zn
Total Zn (13598.7216)
60000000
50000000
40000000
30000000
20000000
10000000
10
15
20
Elution Volume (mL) (0.0303056)

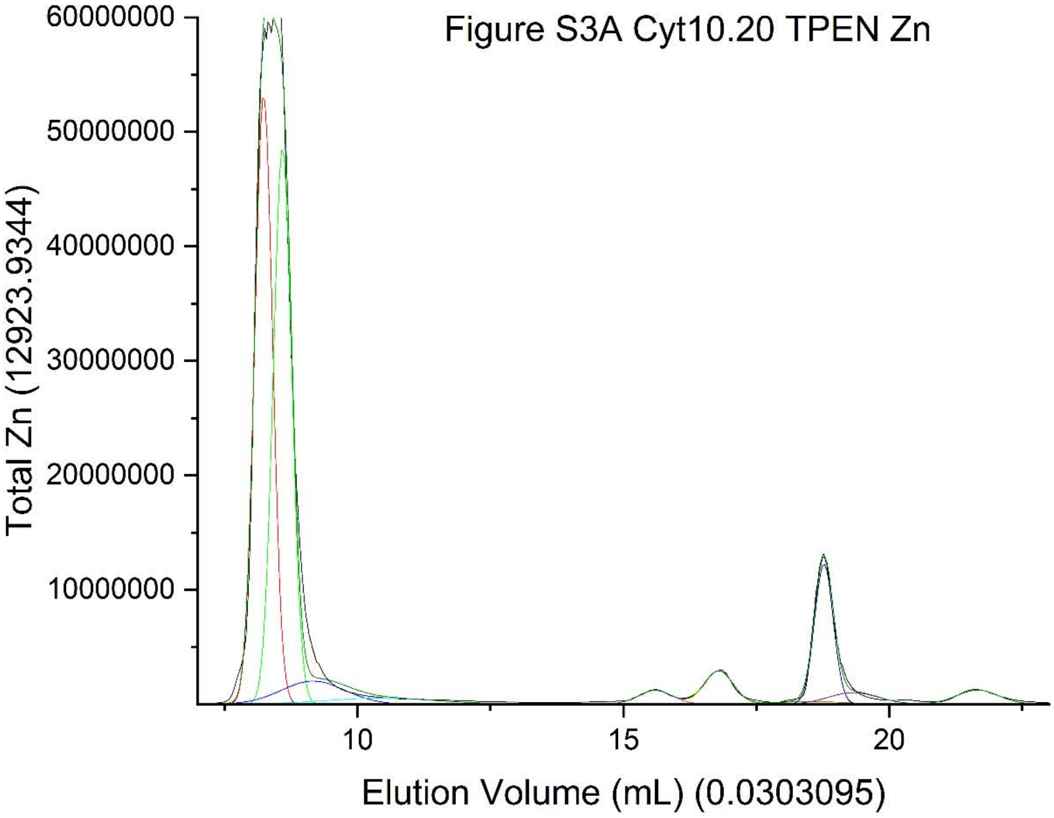
Figure S3A Cyt10.20 TPEN Zn
60000000
50000000
40000000
30000000
20000000
10000000
Total Zn (12923.9344)
10
15
20
Elution Volume (mL) (0.0303095)

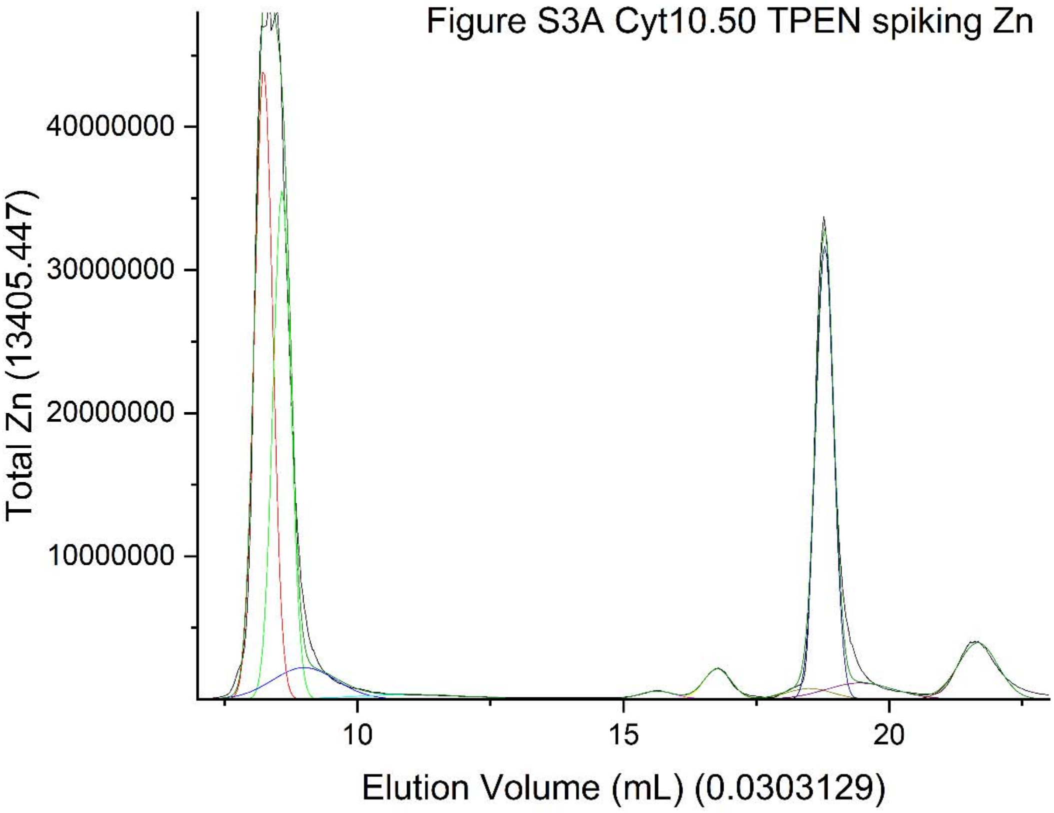
Figure S3A Cyt10.50 TPEN spiking Zn
Total Zn (13405.447)
40000000
30000000
20000000
10000000
10
15
20
Elution Volume (mL) (0.0303129)

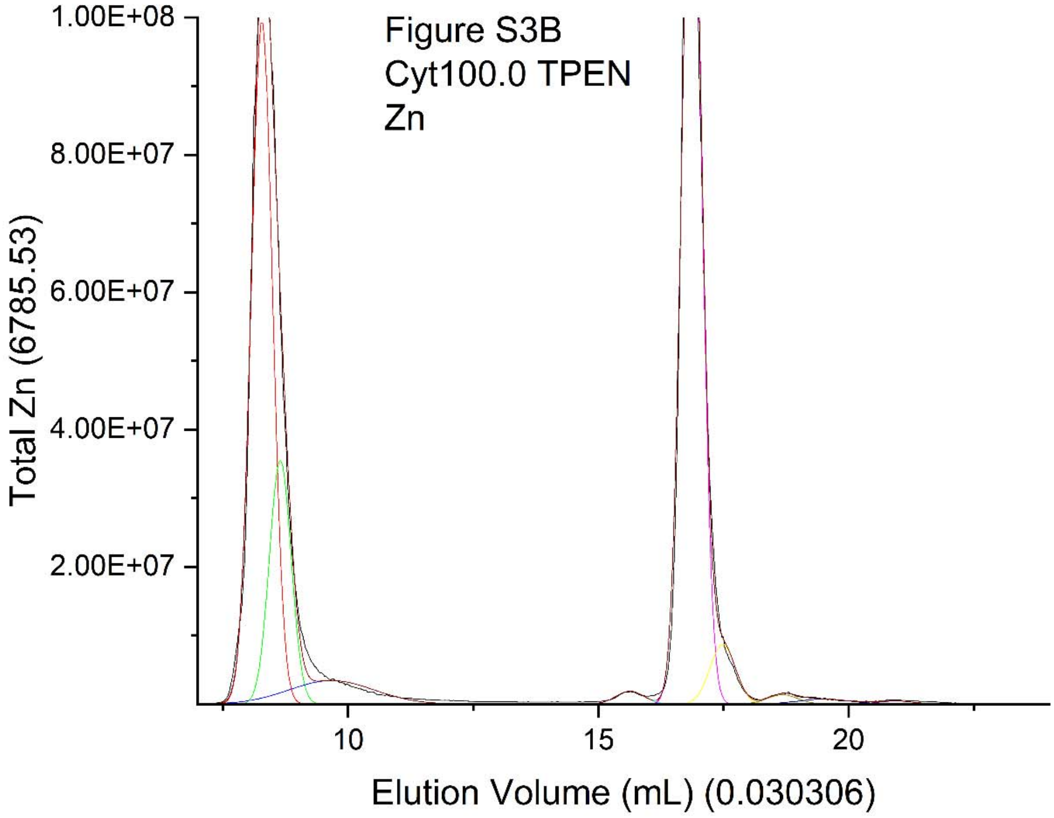
Figure S3B
Cyt100.0 TPEN
Zn
Total Zn (6785.53)
1.00E+08
8.00E+07
6.00E+07
4.00E+07
2.00E+07
10
15
20
Elution Volume (mL) (0.030306)

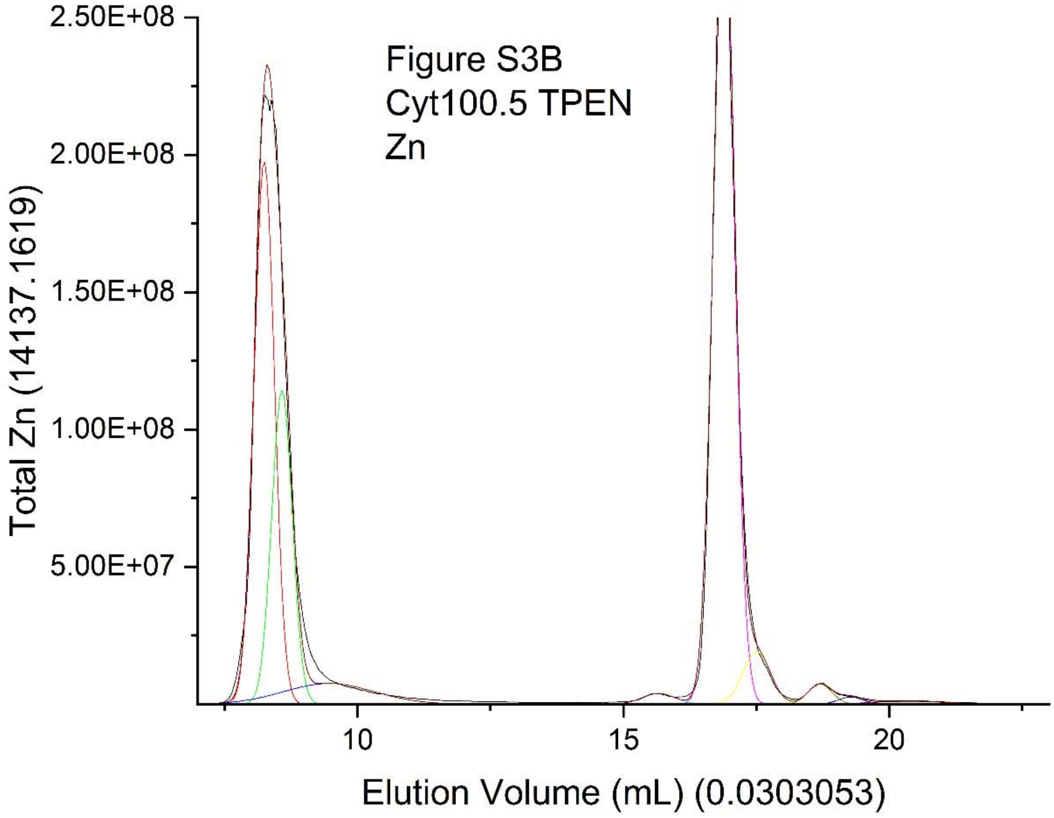
Figure S3B
Cyt100.5 TPEN
Zn
Total Zn (14137.1619)
2.50E+08
2.00E+08
1.50E+08
1.00E+08
5.00E+07
10
15
20
Elution Volume (mL) (0.0303053)

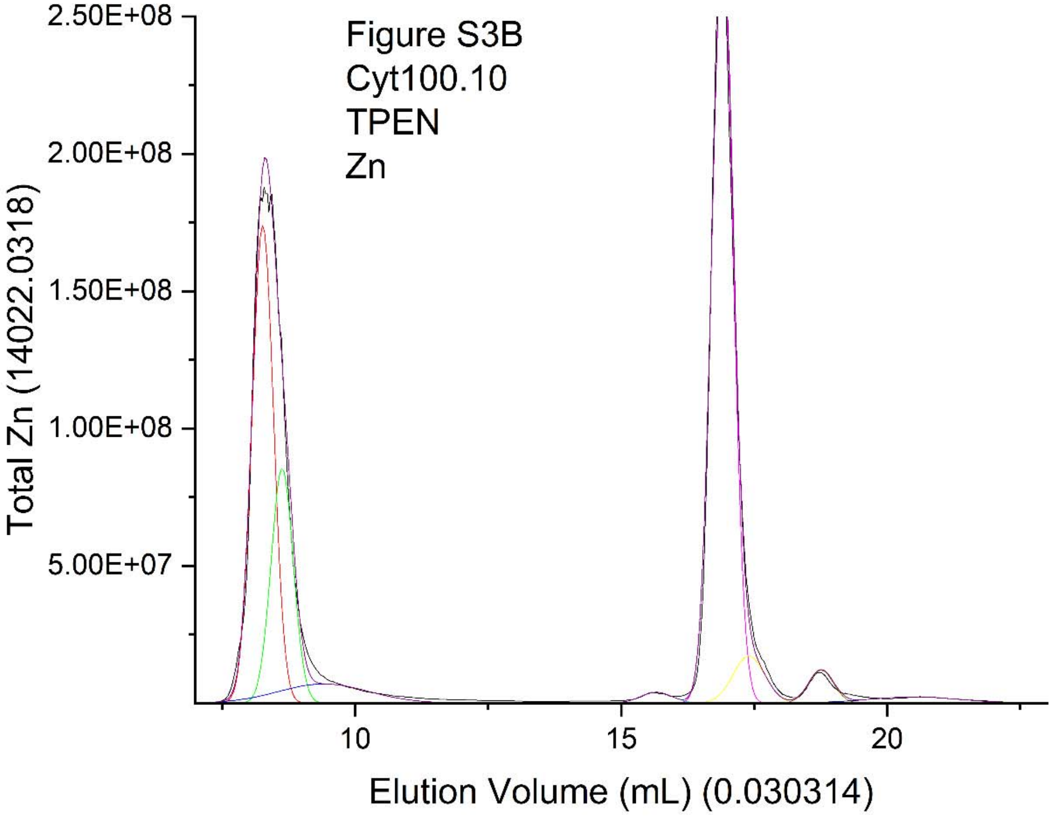
Figure S3B
Cyt100.10
TPEN
Zn
2.50E+08
2.00E+08
1.50E+08
1.00E+08
5.00E+07
Total Zn (14022.0318)
10
15
20
Elution Volume (mL) (0.030314)

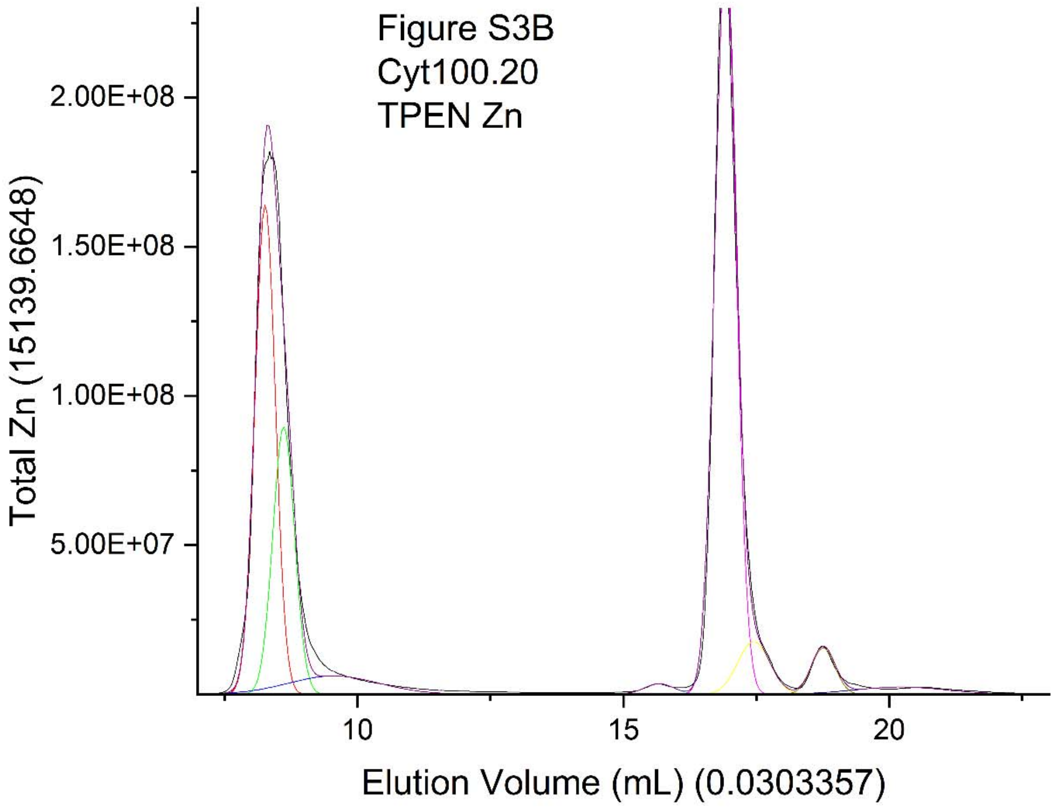
Figure S3B
Cyt100.20
TPEN Zn
Total Zn (15139.6648)
2.00E+08
1.50E+08
1.00E+08
5.00E+07
10
15
20
Elution Volume (mL) (0.0303357)

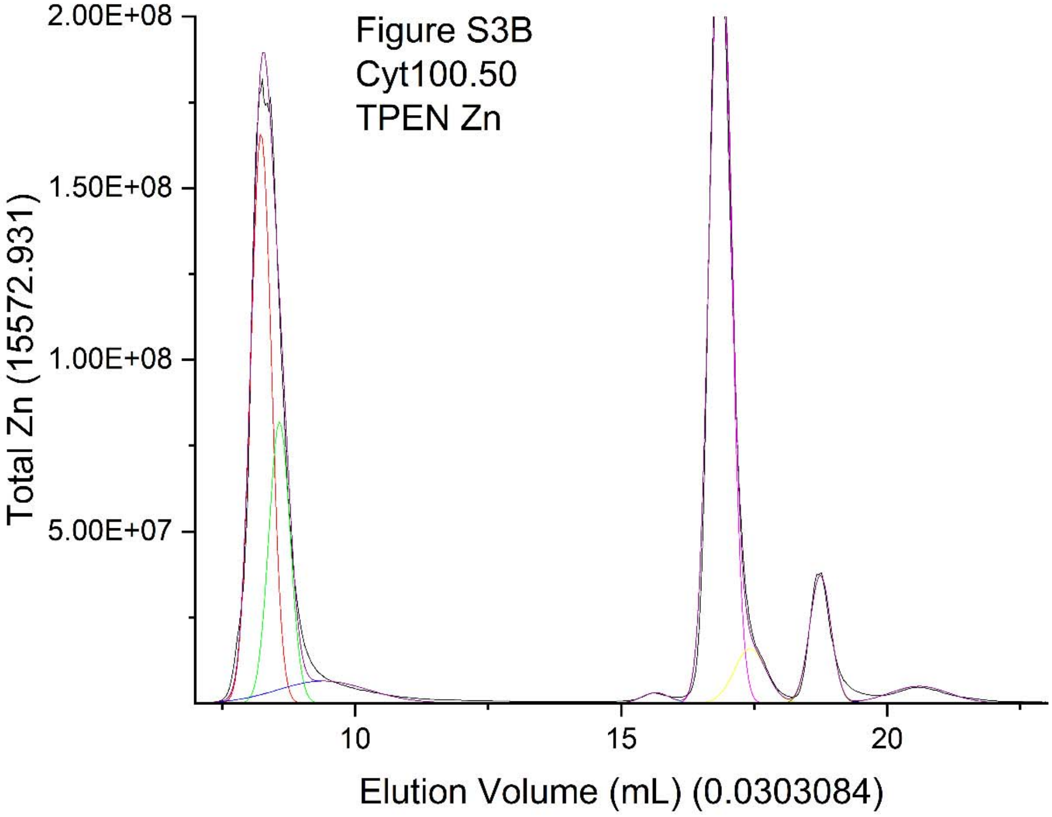
Figure S3B
Cyt100.50
TPEN Zn
2.00E+08
1.50E+08
1.00E+08
5.00E+07
Total Zn (15572.931)
10
15
20
Elution Volume (mL) (0.0303084)

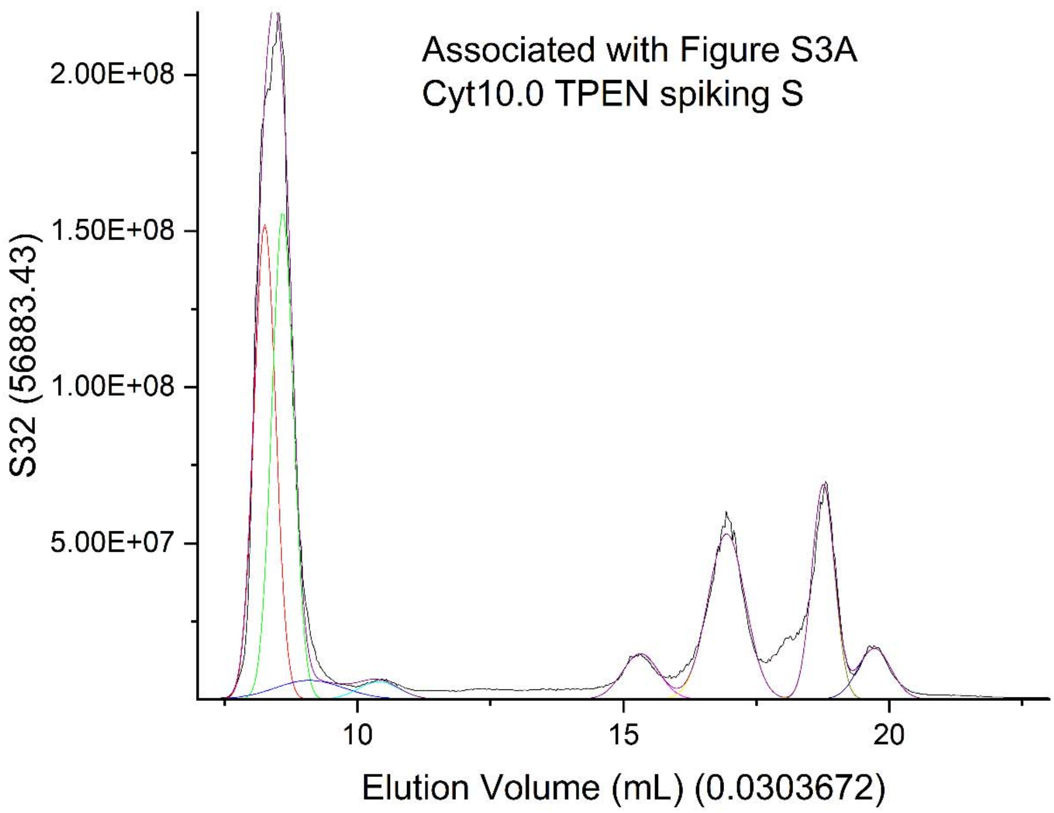
Associated with Figure S3A
Cyt10.0 TPEN spiking S
S32 (56883.43)
2.00E+08
1.50E+08
1.00E+08
5.00E+07
10
15
20
Elution Volume (mL) (0.0303672)

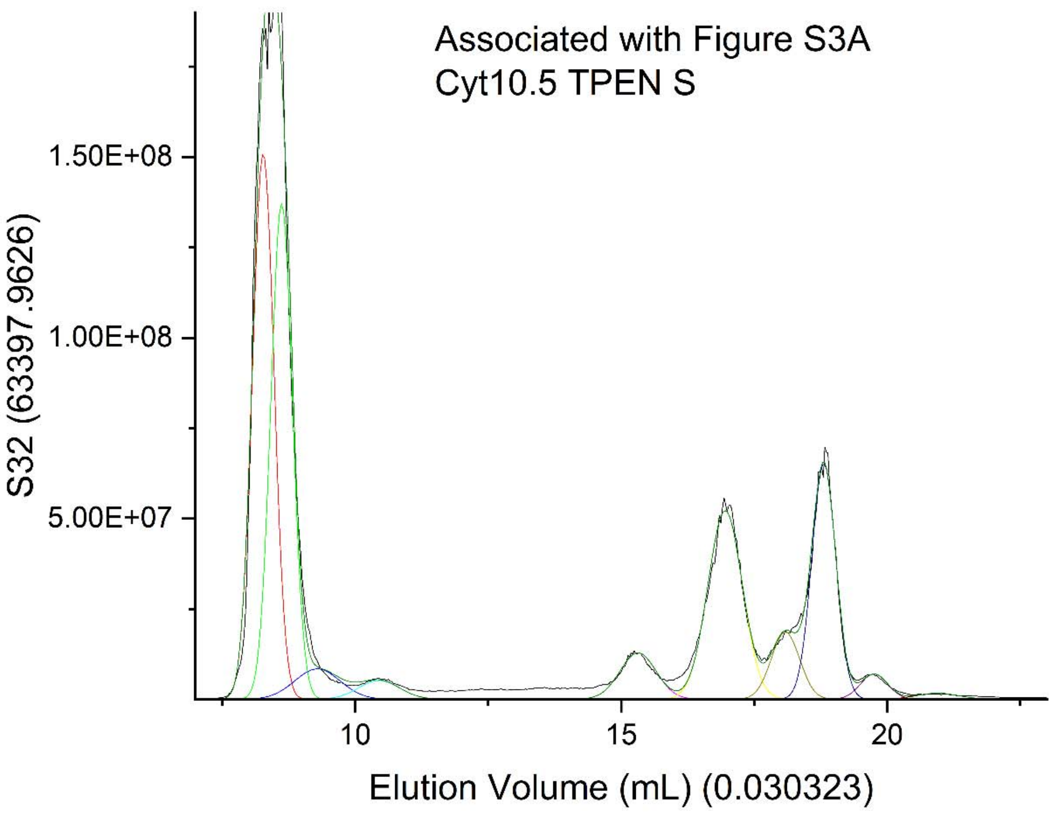
Associated with Figure S3A
Cyt10.5 TPEN S
S32 (63397.9626)
1.50E+08
1.00E+08
5.00E+07
10
15
20
Elution Volume (mL) (0.030323)

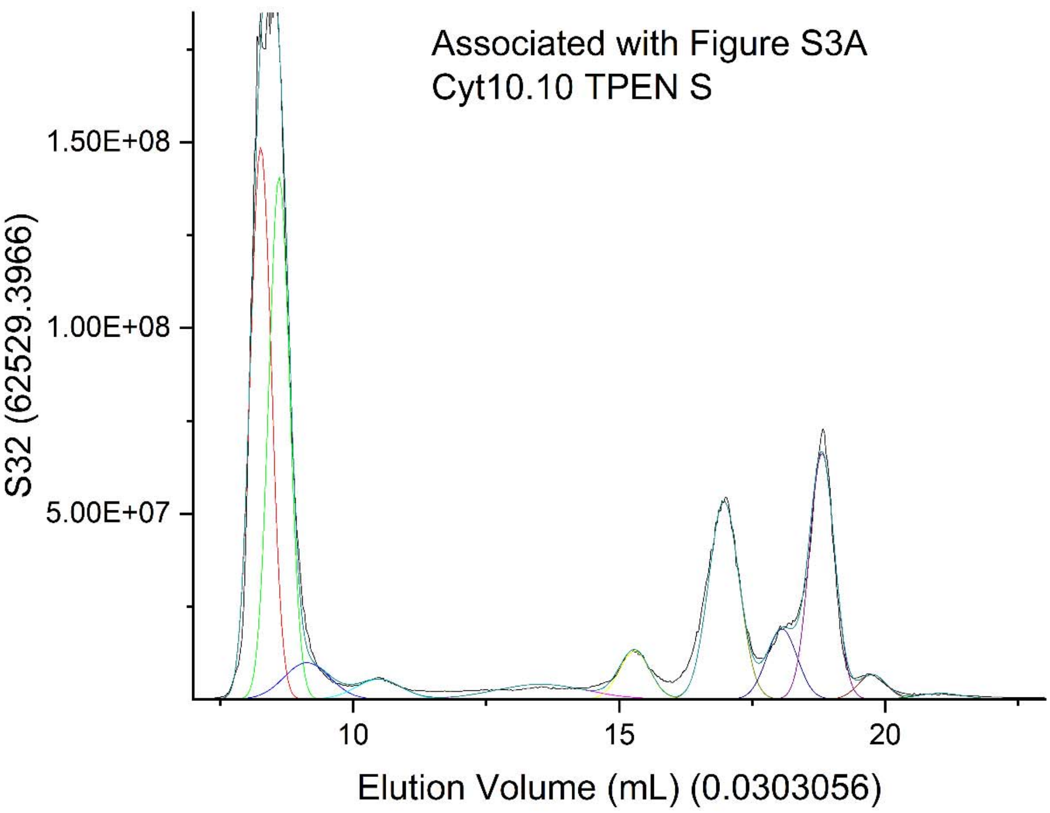
Associated with Figure S3A
Cyt10.10 TPEN S
1.50E+08
1.00E+08
5.00E+07
S32 (62529.3966)
10
15
20
Elution Volume (mL) (0.0303056)

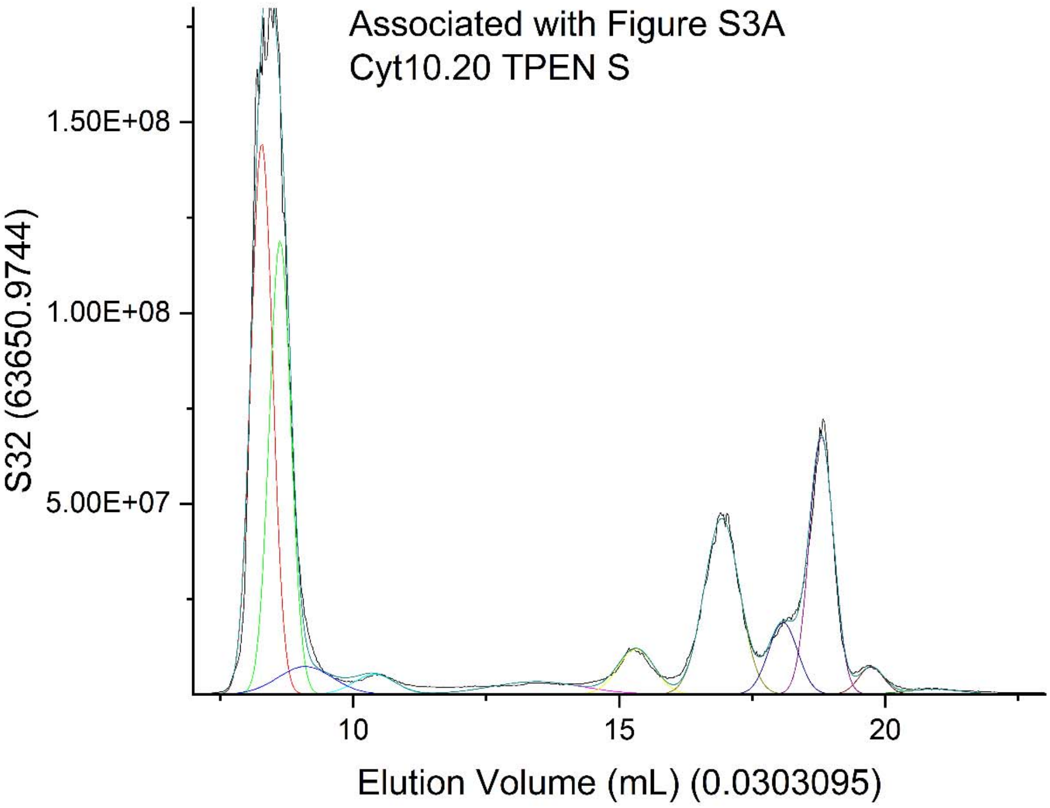
Associated with Figure S3A
Cyt10.20 TPEN S
1.50E+08
1.00E+08
5.00E+07
S32 (63650.9744)
10
15
20
Elution Volume (mL) (0.0303095)

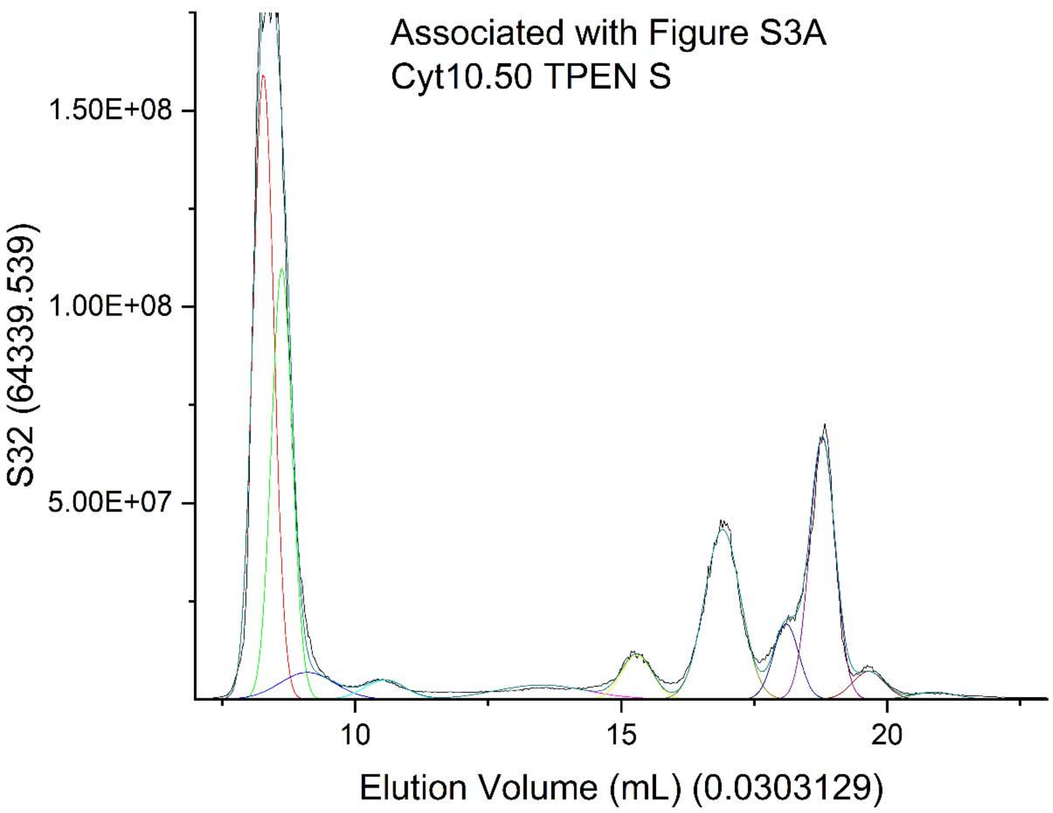
Associated with Figure S3A
Cyt10.50 TPEN S
1.50E+08
1.00E+08
5.00E+07
S32 (64339.539)
10
15
20
Elution Volume (mL) (0.0303129)

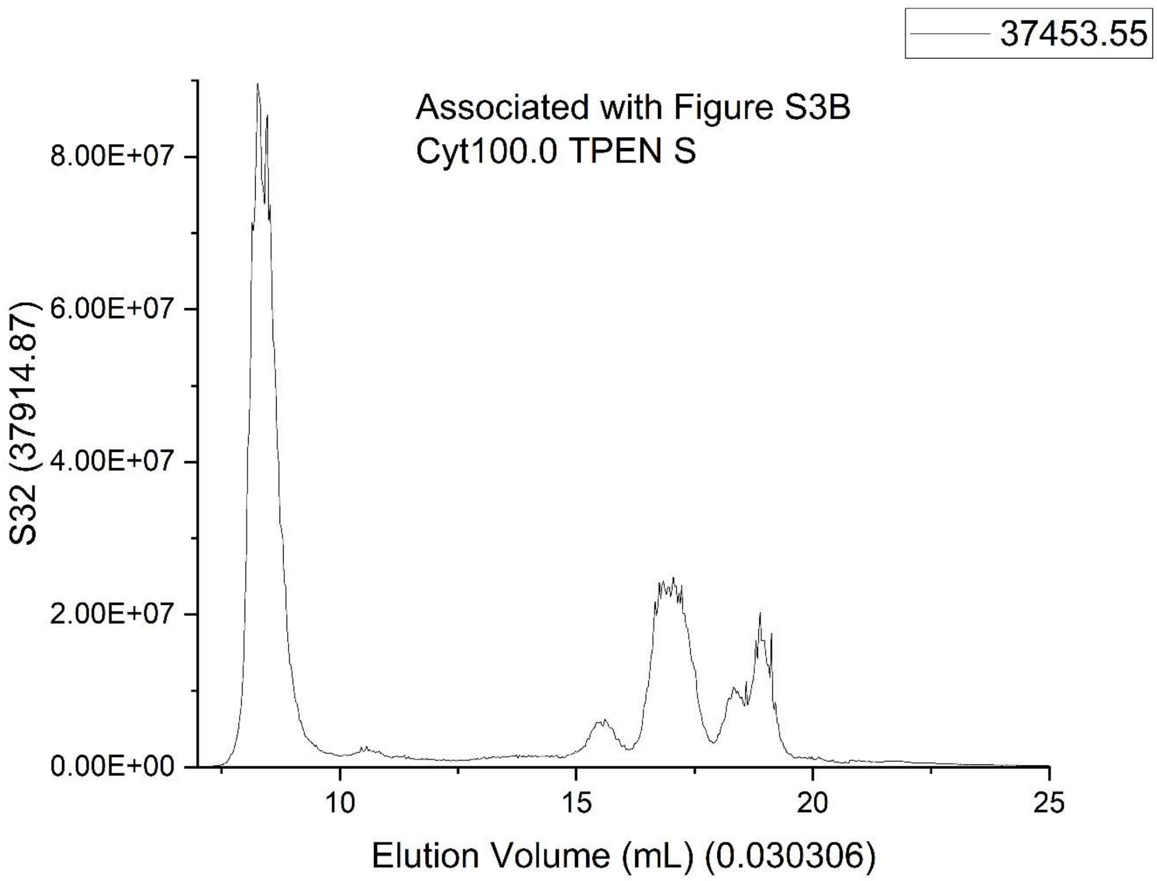

37453.55
Associated with Figure S3B
Cyt100.0 TPEN S
S32 (37914.87)
8.00E+07
6.00E+07
4.00E+07
2.00E+07
0.00E+00
10
15
20
25
Elution Volume (mL) (0.030306)

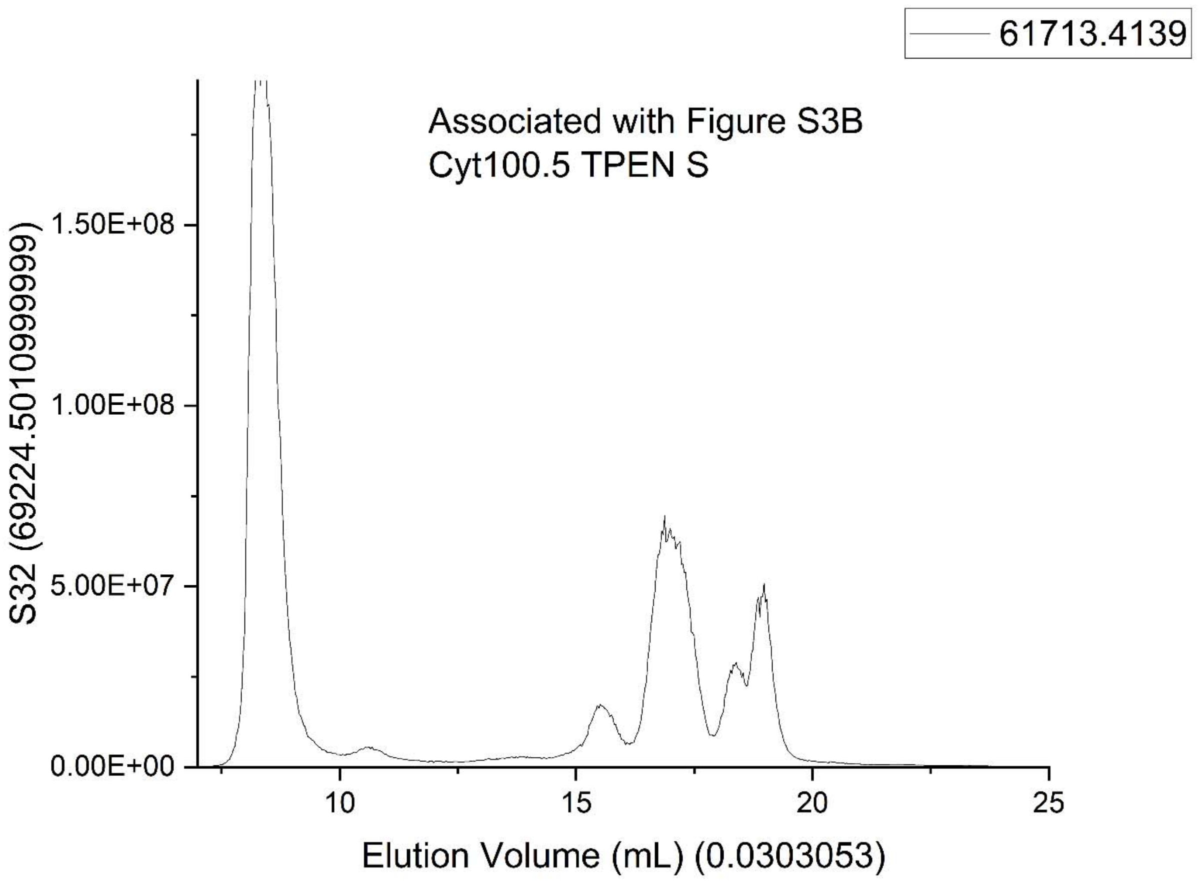
61713.4139
Associated with Figure S3B
Cyt100.5 TPEN S
S32 (69224.5010999999)
1.50E+08
1.00E+08
5.00E+07
0.00E+00
10
15
20
25
Elution Volume (mL) (0.0303053)

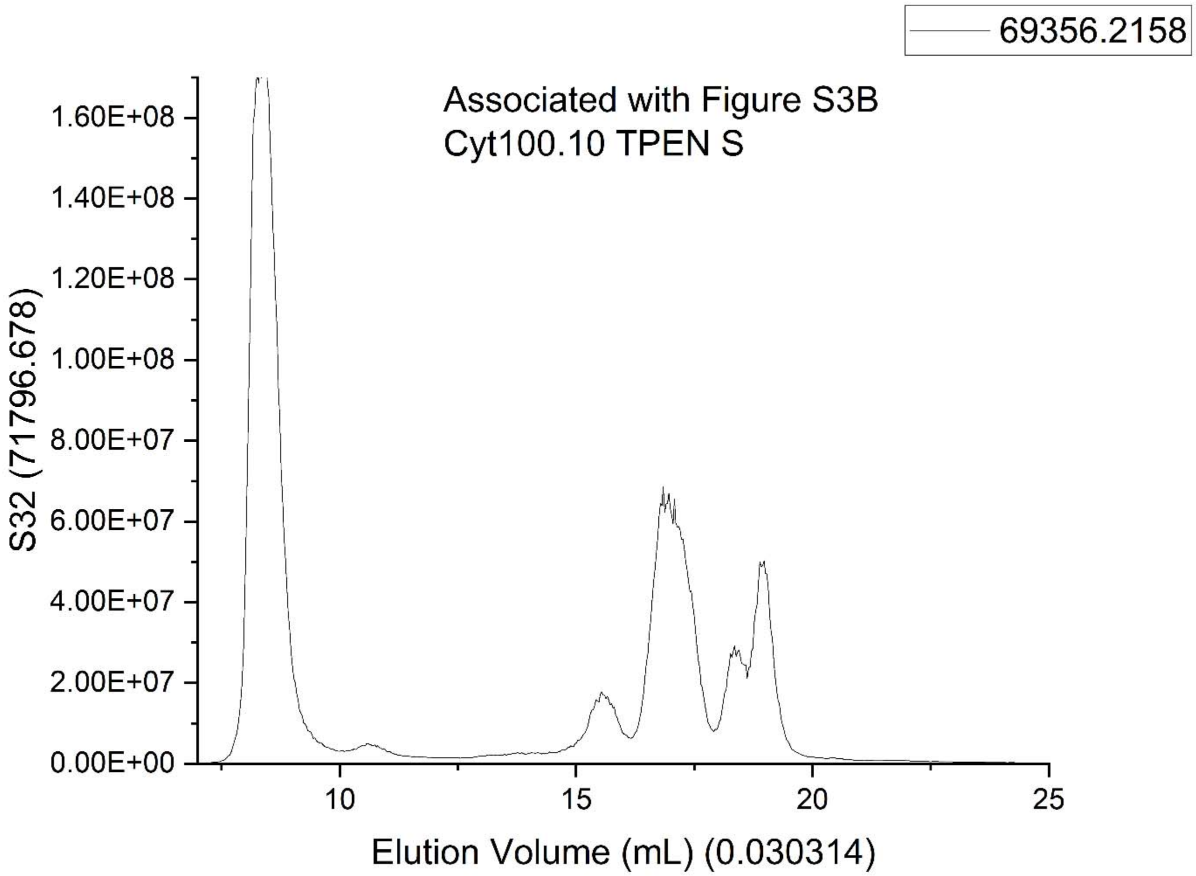

69356.2158
Associated with Figure S3B
Cyt100.10 TPEN S
S32 (71796.678)
1.60E+08
1.40E+08
1.20E+08
1.00E+08
8.00E+07
6.00E+07
4.00E+07
2.00E+07
0.00E+00
10
15
20
25
Elution Volume (mL) (0.030314)

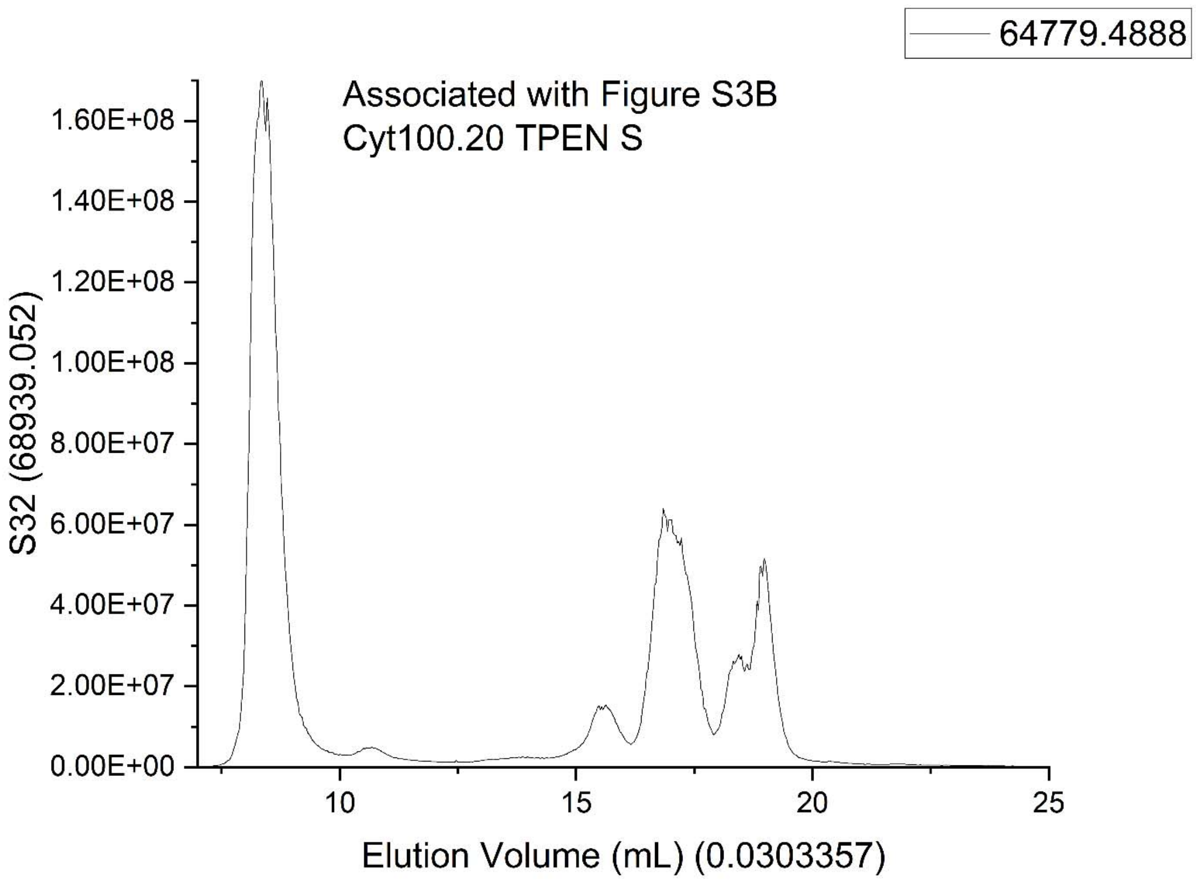

64779.4888
Associated with Figure S3B
Cyt100.20 TPEN S
S32 (68939.052)
1.60E+08
1.40E+08
1.20E+08
1.00E+08
8.00E+07
6.00E+07
4.00E+07
2.00E+07
0.00E+00
10
15
20
25
Elution Volume (mL) (0.0303357)

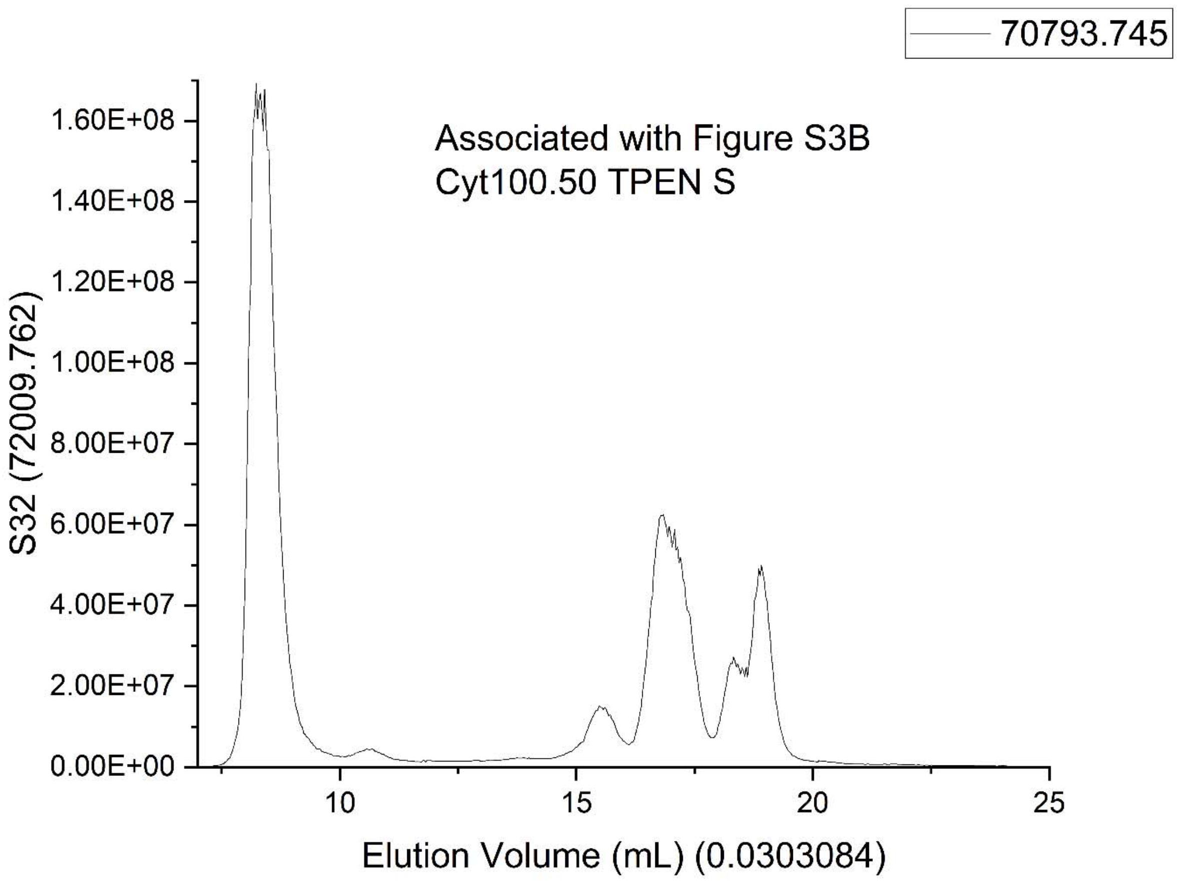
70793.745
Associated with Figure S3B
Cyt100.50 TPEN S
S32 (72009.762)
1.60E+08
1.40E+08
1.20E+08
1.00E+08
8.00E+07
6.00E+07
4.00E+07
2.00E+07
0.00E+00
10
15
20
25
Elution Volume (mL) (0.0303084)

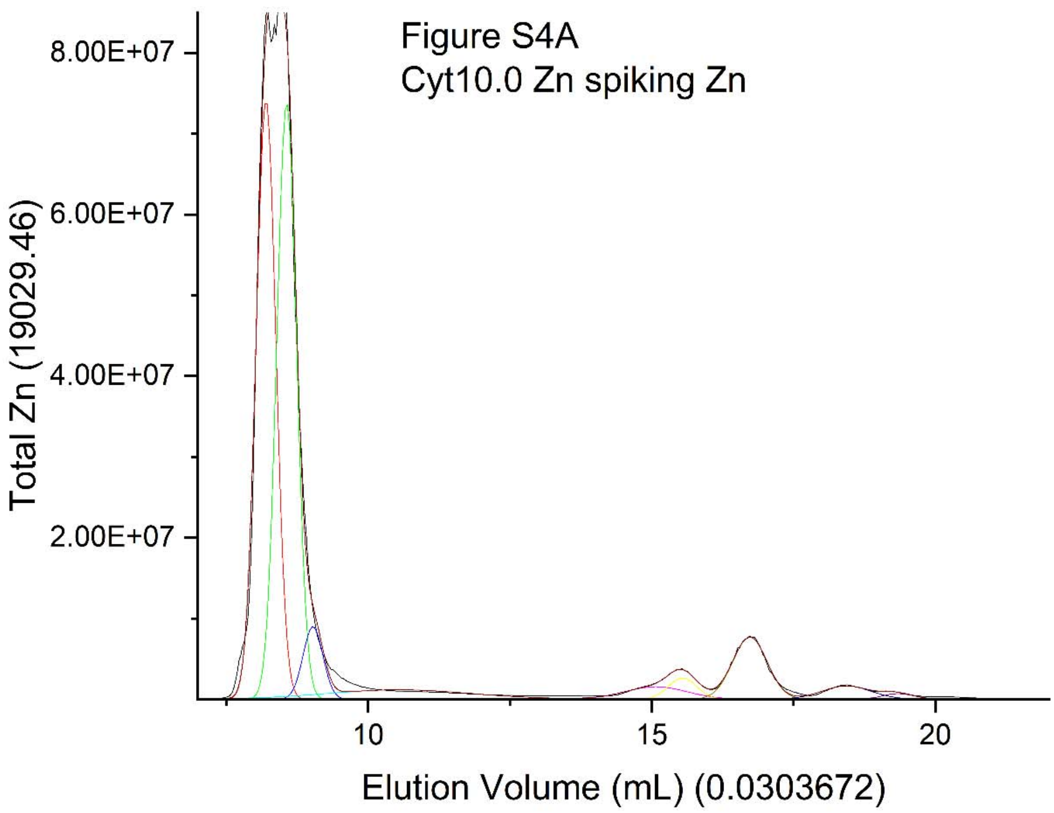
Figure S4A
Cyt10.0 Zn spiking Zn
Total Zn (19029.46)
8.00E+07
6.00E+07
4.00E+07
2.00E+07
10
15
20
Elution Volume (mL) (0.0303672)

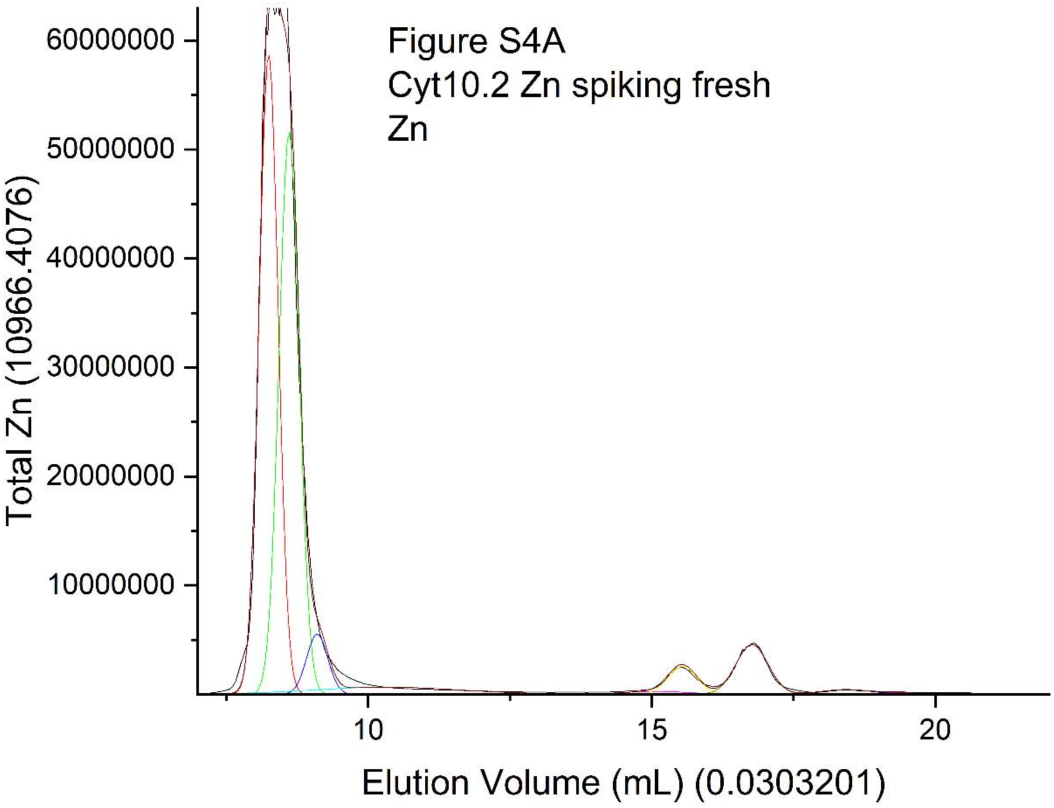
Figure S4A
Cyt10.2 Zn spiking fresh
Zn
60000000
50000000
40000000
30000000
20000000
10000000
Total Zn (10966.4076)
10
15
20
Elution Volume (mL) (0.0303201)

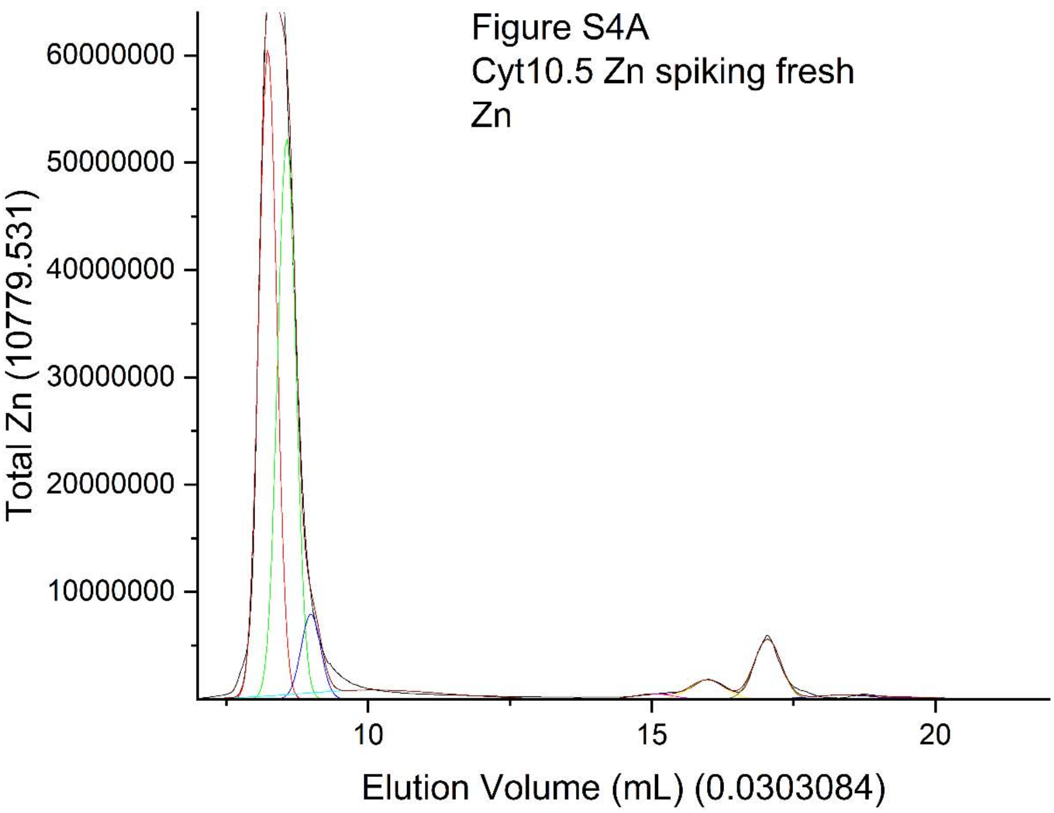

Figure S4A
Cyt10.5 Zn spiking fresh
Zn
Total Zn (10779.531)
60000000
50000000
40000000
30000000
20000000
10000000
10
15
20
Elution Volume (mL) (0.0303084)

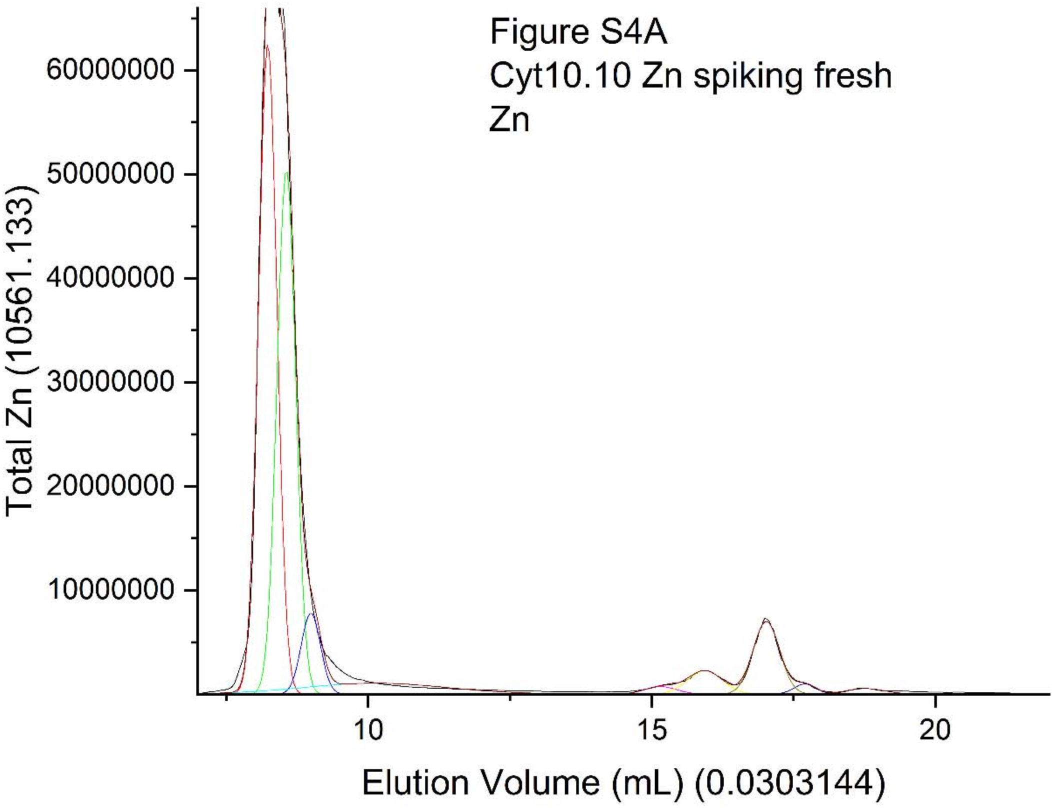

Figure S4A
Cyt10.10 Zn spiking fresh
Zn
Total Zn (10561.133)
60000000
50000000
40000000
30000000
20000000
10000000
10
15
20
Elution Volume (mL) (0.0303144)

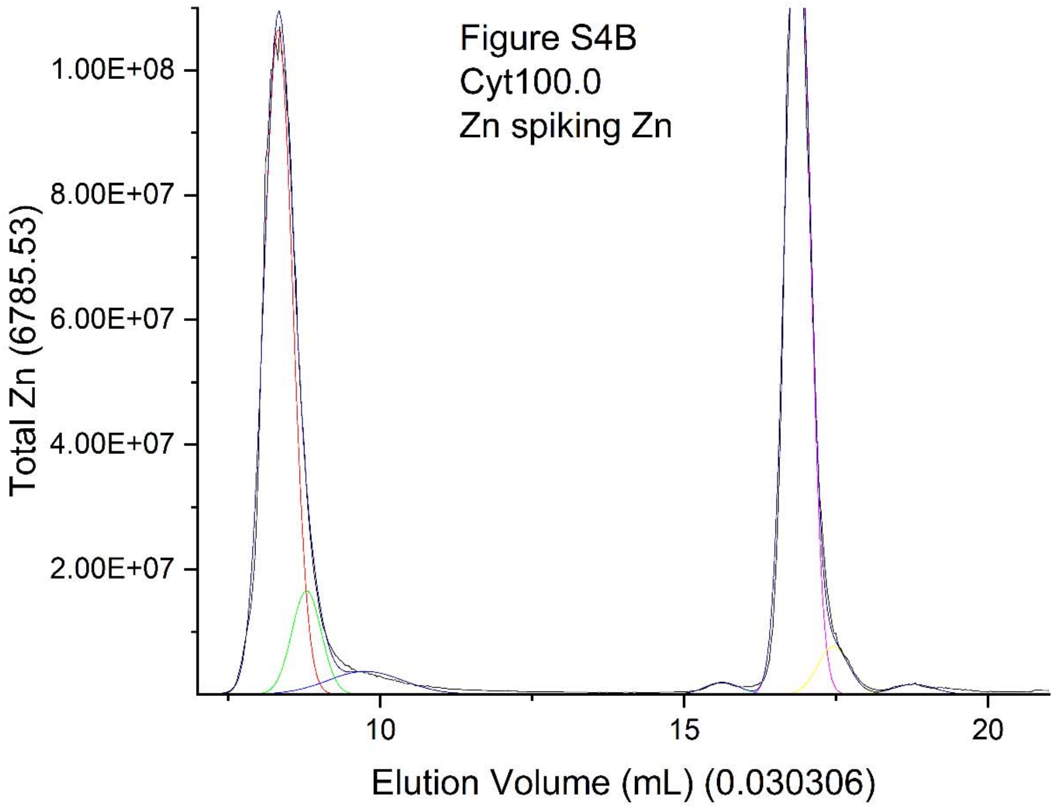
Figure S4B
Cyt100.0
Zn spiking Zn
Total Zn (6785.53)
1.00E+08
8.00E+07
6.00E+07
4.00E+07
2.00E+07
10
15
20
Elution Volume (mL) (0.030306)

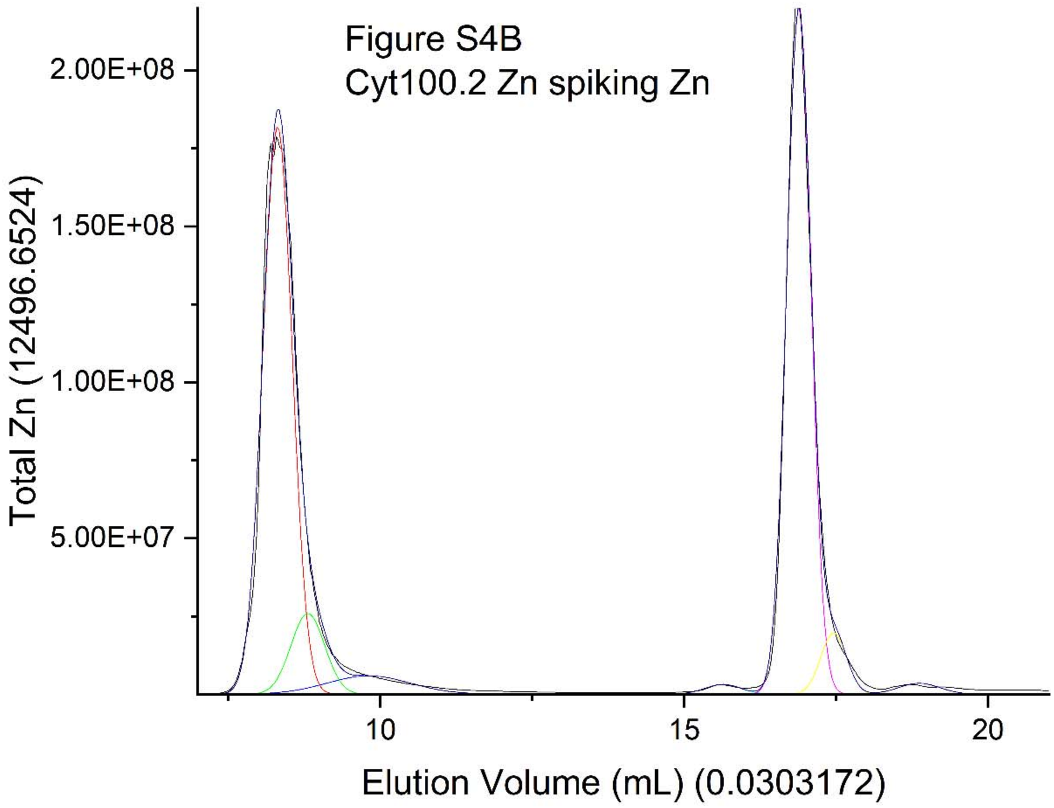
Figure S4B
Cyt100.2 Zn spiking Zn
Total Zn (12496.6524)
2.00E+08
1.50E+08
1.00E+08
5.00E+07
10
15
20
Elution Volume (mL) (0.0303172)

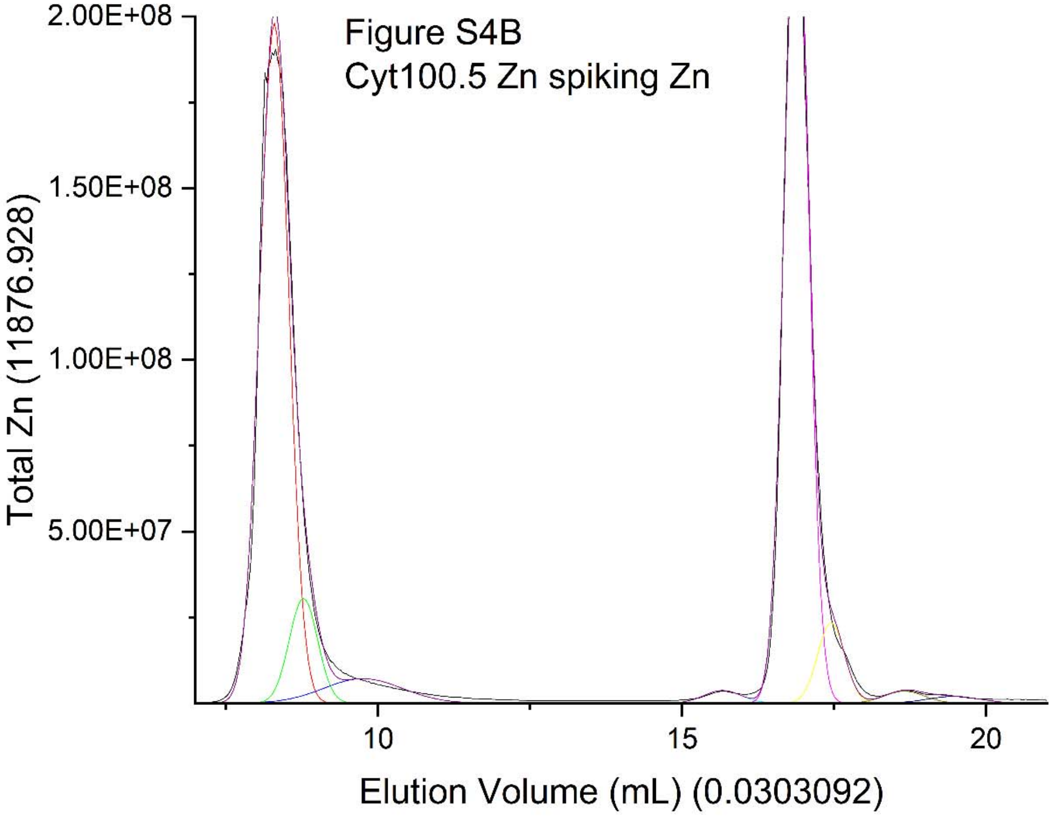
Figure S4B
Cyt100.5 Zn spiking Zn
2.00E+08
1.50E+08
1.00E+08
5.00E+07
Total Zn (11876.928)
10
15
20
Elution Volume (mL) (0.0303092)

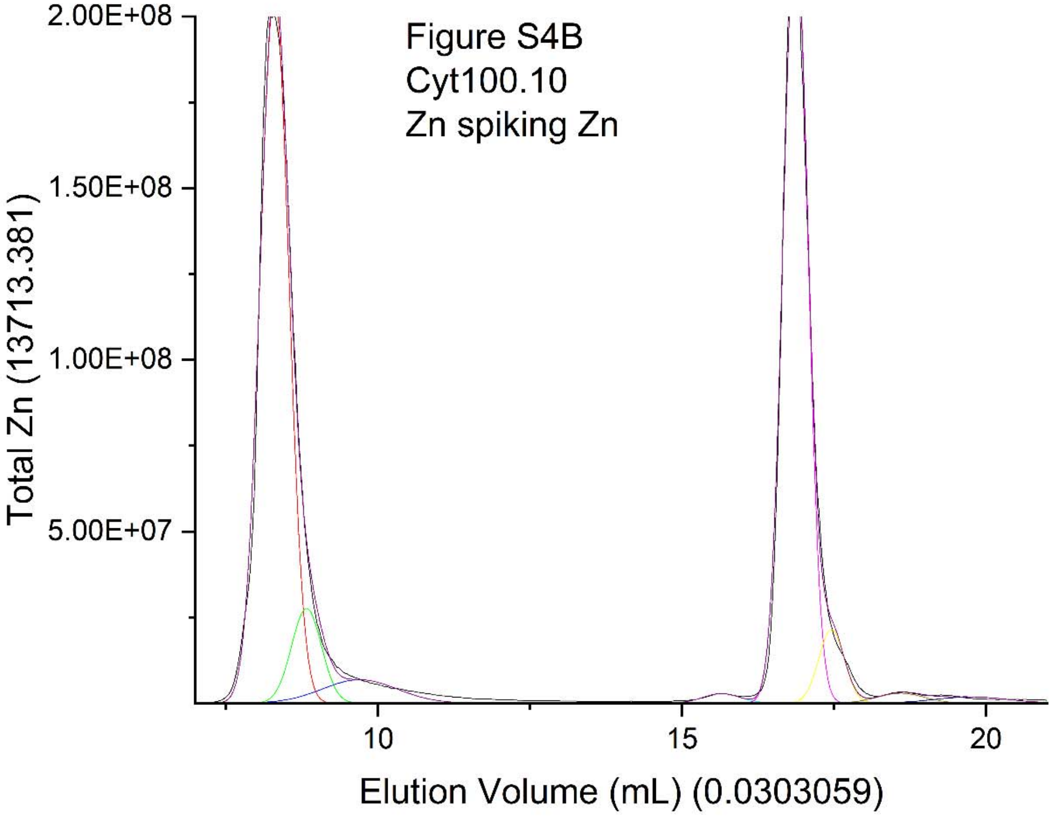

Figure S4B
Cyt100.10
Zn spiking Zn
Total Zn (13713.381)
2.00E+08
1.50E+08
1.00E+08
5.00E+07
10
15
20
Elution Volume (mL) (0.0303059)

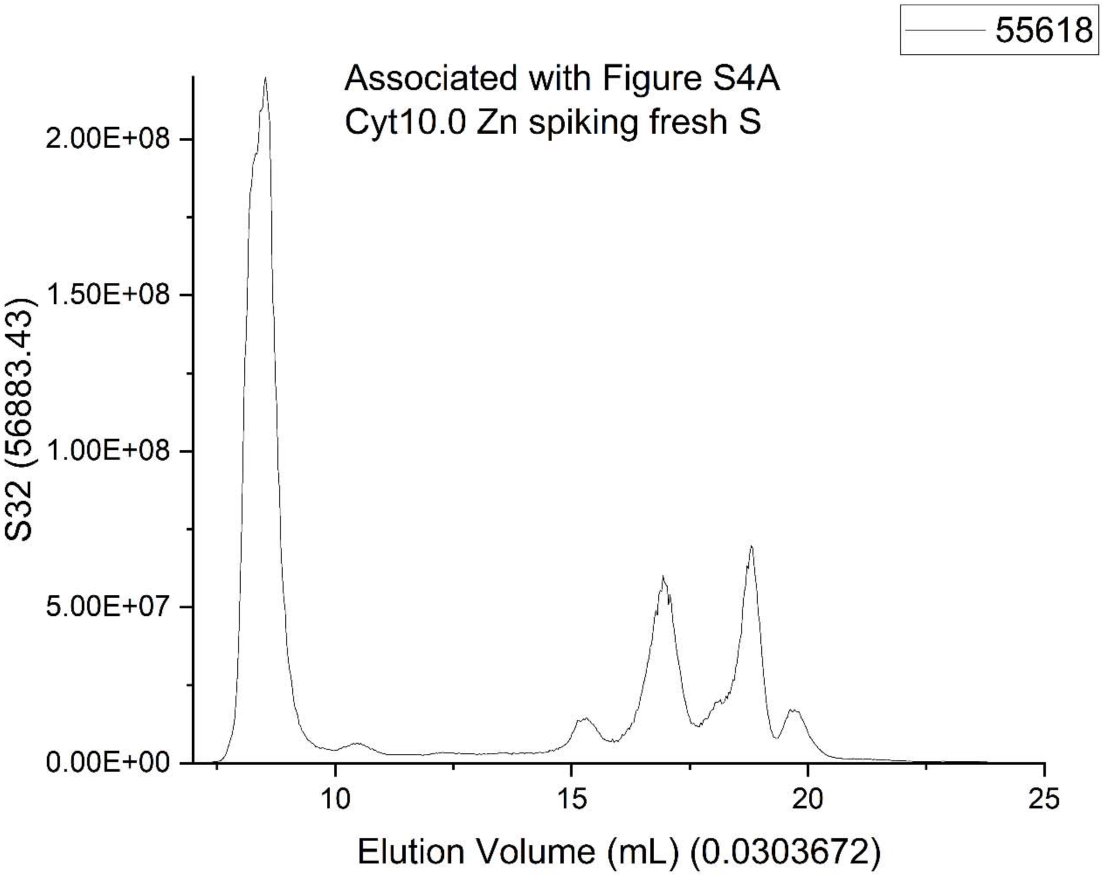

55618
Associated with Figure S4A
Cyt10.0 Zn spiking fresh S
S32 (56883.43)
2.00E+08
1.50E+08
1.00E+08
5.00E+07
0.00E+00
10
15
20
25
Elution Volume (mL) (0.0303672)

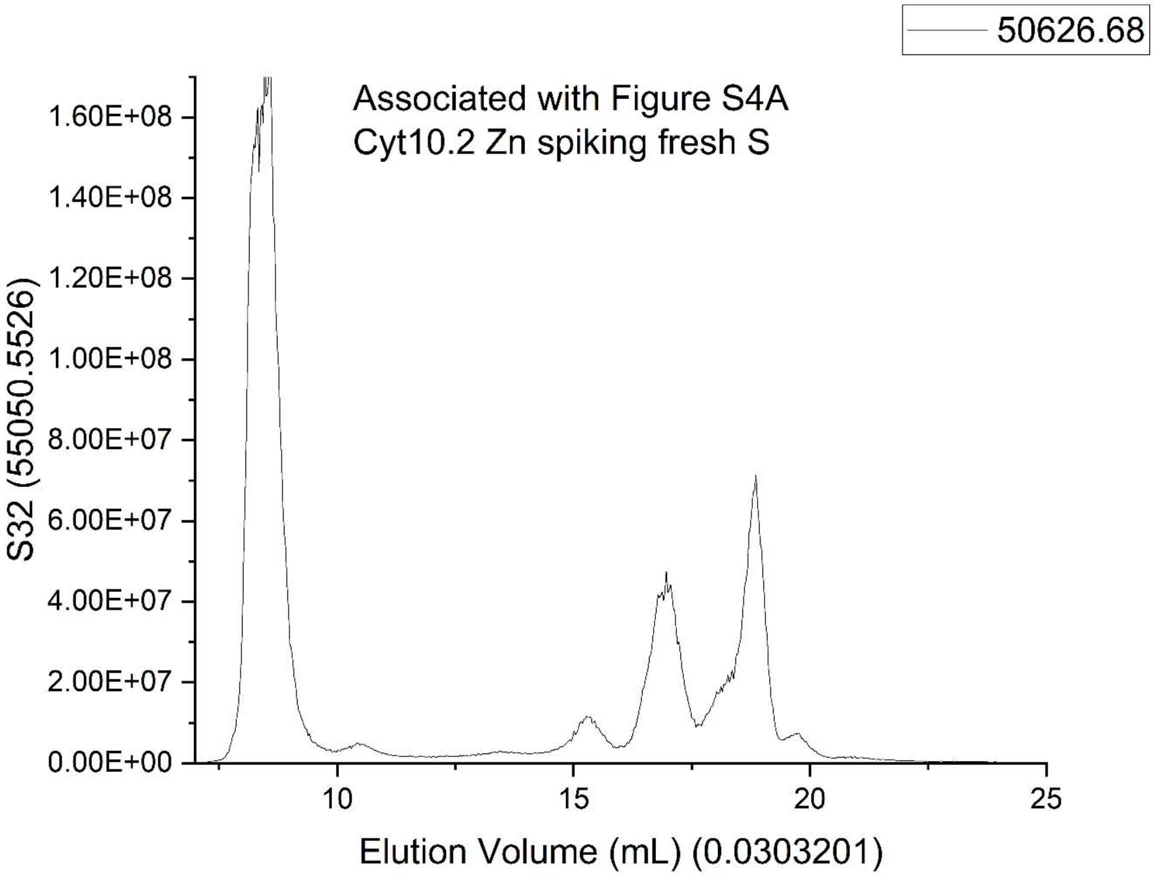
50626.68
Associated with Figure S4A
Cyt10.2 Zn spiking fresh S
S32 (55050.5526)
1.60E+08
1.40E+08
1.20E+08
1.00E+08
8.00E+07
6.00E+07
4.00E+07
2.00E+07
0.00E+00
10
15
20
25
Elution Volume (mL) (0.0303201)

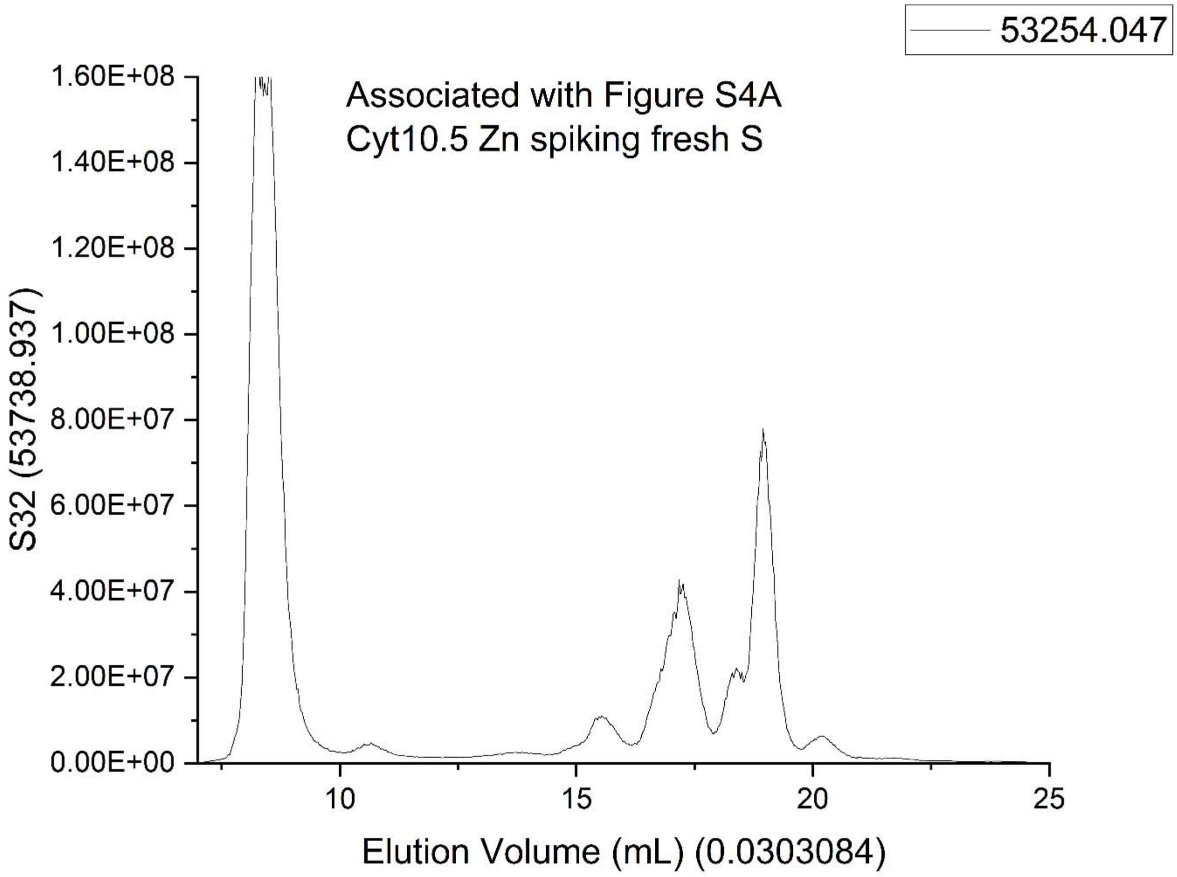

53254.047
Associated with Figure S4A
Cyt10.5 Zn spiking fresh S
S32 (53738.937)
1.60E+08
1.40E+08
1.20E+08
1.00E+08
8.00E+07
6.00E+07
4.00E+07
2.00E+07
0.00E+00
10
15
20
25
Elution Volume (mL) (0.0303084)

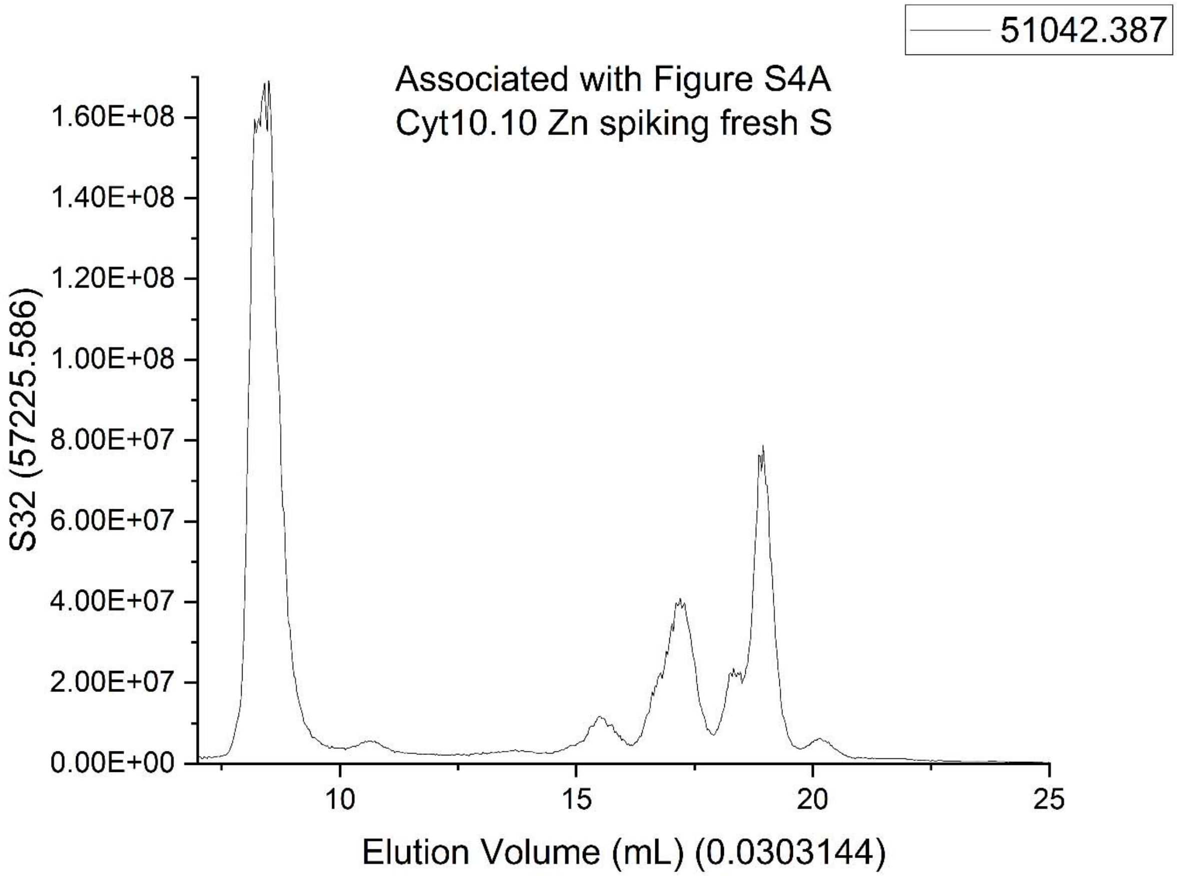
51042.387
Associated with Figure S4A
Cyt10.10 Zn spiking fresh S
S32 (57225.586)
1.60E+08
1.40E+08
1.20E+08
1.00E+08
8.00E+07
6.00E+07
4.00E+07
2.00E+07
0.00E+00
10
15
20
25
Elution Volume (mL) (0.0303144)

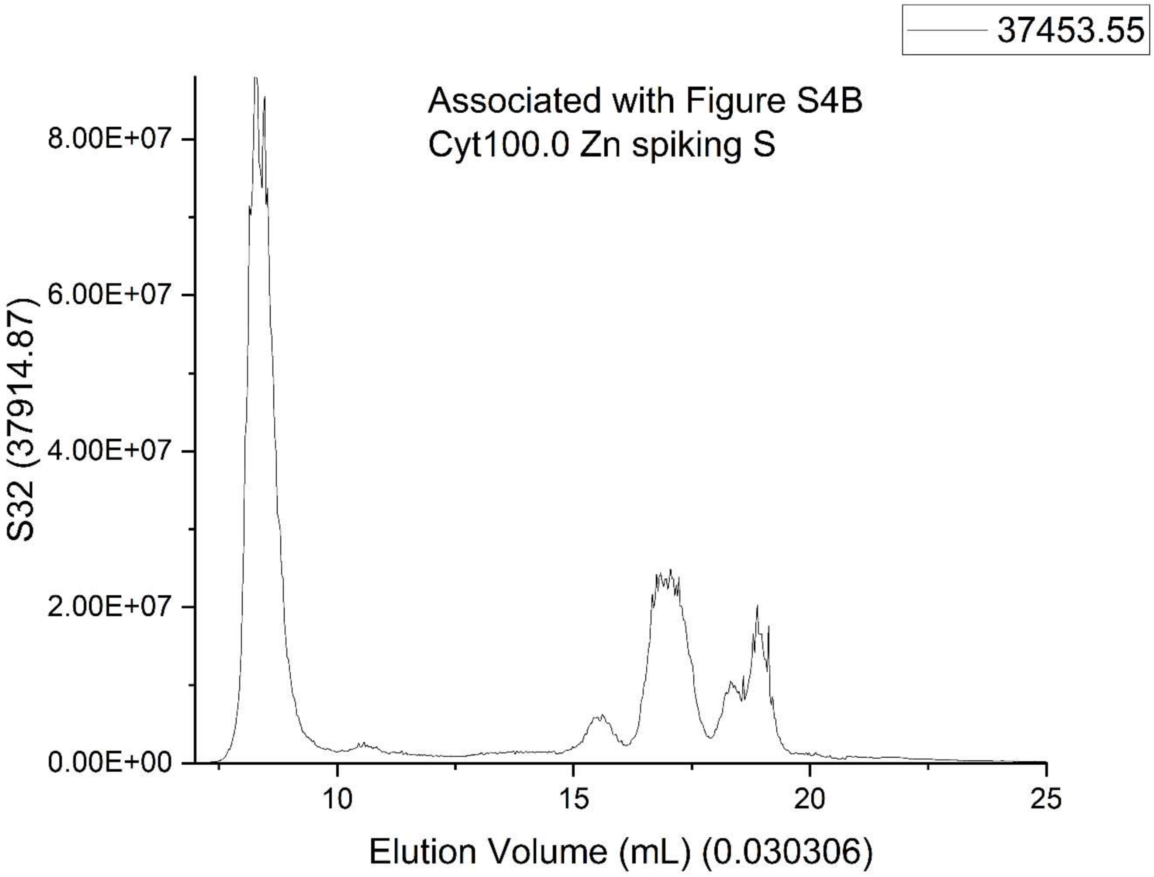
37453.55
Associated with Figure S4B
Cyt100.0 Zn spiking S
S32 (37914.87)
8.00E+07
6.00E+07
4.00E+07
2.00E+07
0.00E+00
10
15
20
25
Elution Volume (mL) (0.030306)

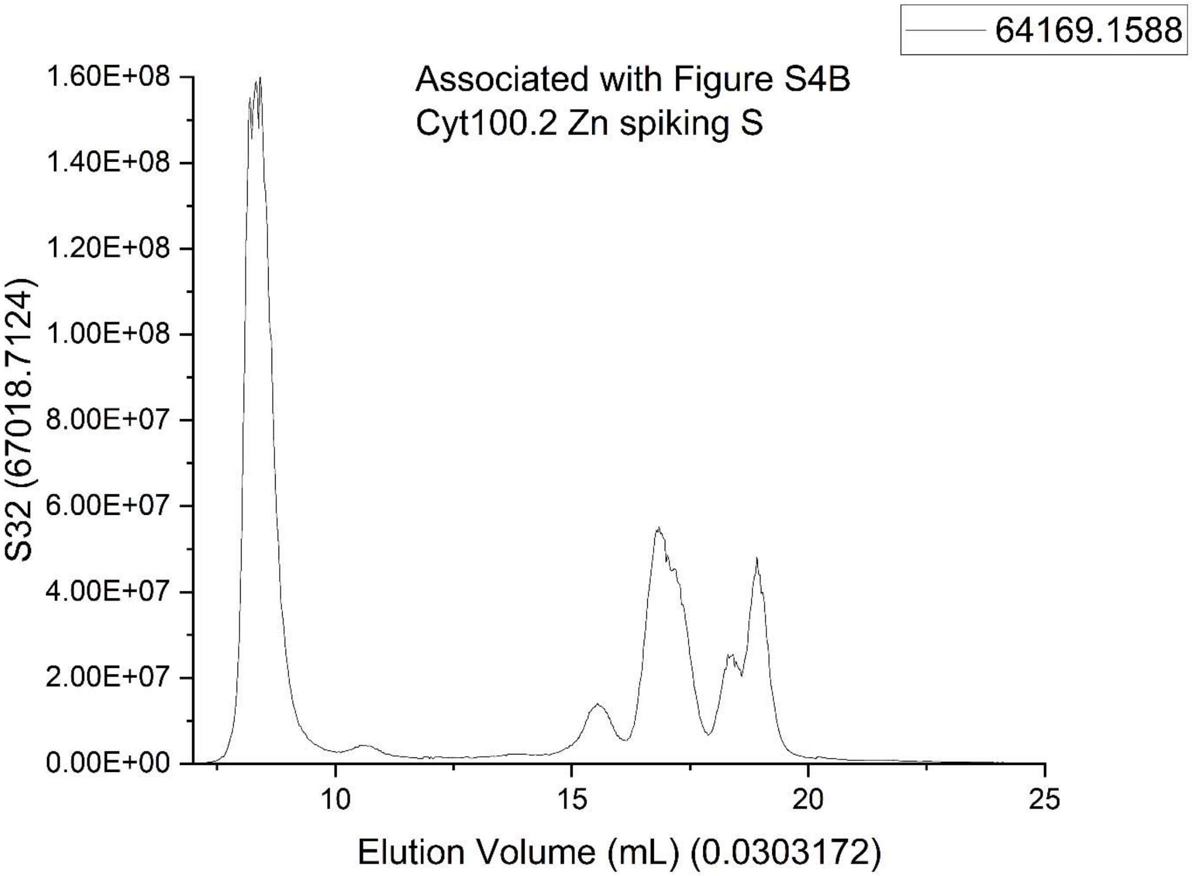

64169.1588
Associated with Figure S4B
Cyt100.2 Zn spiking S
S32 (67018.7124)
1.60E+08
1.40E+08
1.20E+08
1.00E+08
8.00E+07
6.00E+07
4.00E+07
2.00E+07
0.00E+00
10
15
20
25
Elution Volume (mL) (0.0303172)

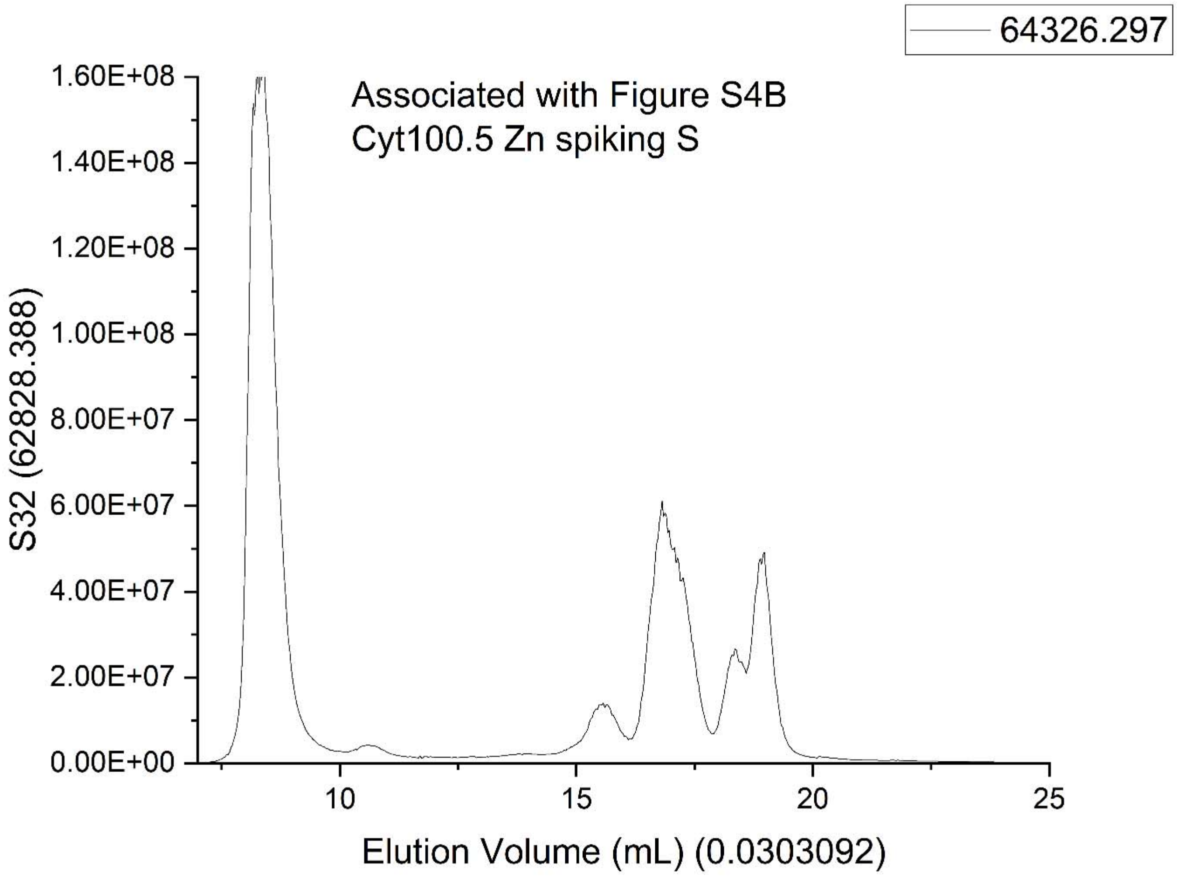

64326.297
Associated with Figure S4B
Cyt100.5 Zn spiking S
S32 (62828.388)
1.60E+08
1.40E+08
1.20E+08
1.00E+08
8.00E+07
6.00E+07
4.00E+07
2.00E+07
0.00E+00
10
15
20
25
Elution Volume (mL) (0.0303092)

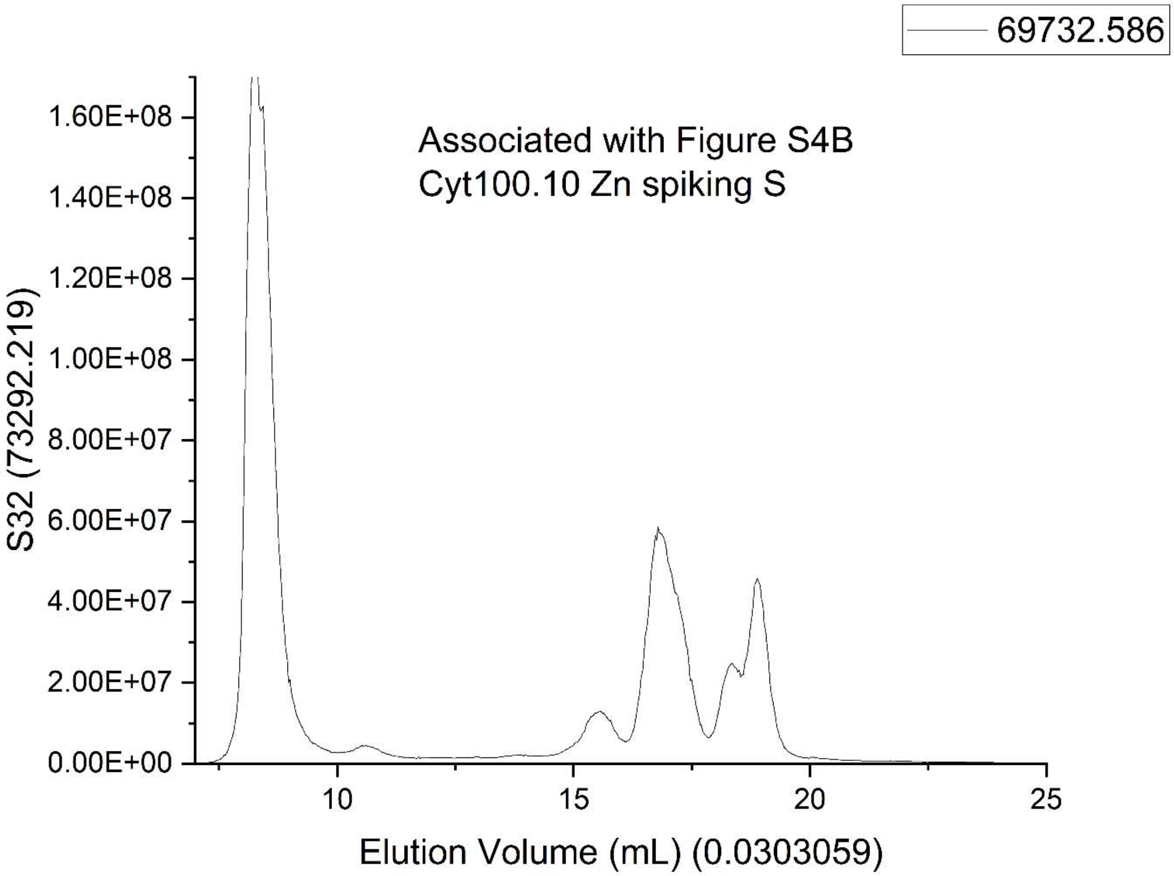
69732.586
Associated with Figure S4B
Cyt100.10 Zn spiking S
S32 (73292.219)
1.60E+08
1.40E+08
1.20E+08
1.00E+08
8.00E+07
6.00E+07
4.00E+07
2.00E+07
0.00E+00
10
15
20
25
Elution Volume (mL) (0.0303059)
